# Disrupting VE-cadherin Y685 phosphorylation inhibits development of experimental diabetic and prediabetic retinopathy

**DOI:** 10.1172/JCI195048

**Published:** 2026-05-15

**Authors:** Yixin Wang, Hongpeng Huang, Feng Shao, Rachana Eshwaran, Miao Qin, Noor Karim, Yonggang Ren, Gergana Dobreva, Hans-Peter Hammes, Thomas Wieland, Yuxi Feng

**Affiliations:** 1 Experimental Pharmacology Mannheim, European Center for Angioscience (ECAS);; 2 Department of Cardiovascular Genomics and Epigenomics, ECAS; and; 3 5th Medical Clinic, Medical Faculty Mannheim, Heidelberg University, Mannheim, Germany.

**Keywords:** Endocrinology, Metabolism, Ophthalmology, Diabetes, Endothelial cells, Retinopathy

## Abstract

Diabetic retinopathy involves early retinal vascular barrier breakdown and pericyte loss, yet the initiating molecular events remain poorly defined. Vascular endothelial cadherin (VE-cadherin), a key regulator of endothelial integrity, is notably reduced in diabetic and prediabetic nucleoside diphosphate kinase B–deficient (NDPKB-deficient) mouse retinas, particularly in the retinal deep capillary layer, and this decline precedes pericyte loss. In vitro, high glucose (HG) and NDPKB deficiency induced VE-cadherin Y685 phosphorylation, promoting its junctional internalization, activating the hexosamine biosynthesis pathway, and increasing angiopoietin 2 (Ang2), resulting in impaired endothelial barrier function and disrupting pericyte attachment. Preventing Y685 phosphorylation through VE-cadherin Y685F mutation blocked these HG- and NDPKB-driven pathological effects. Pharmacological intervention experiments identified protein O-linked β-N-acetyl glucosamine (O-GlcNAc) modification as a mediator of Y685-dependent Ang2 upregulation. In vivo, VE-cadherin Y685F-knockin mice were protected from diabetes- and prediabetes-induced vascular hyperpermeability, exhibited reduced protein O-GlcNAcylation and Ang2 induction, and maintained neuronal function. O-GlcNAc–enriched retinal proteomics further showed that the Y685F mutation restored balanced neurovascular and mitochondrial pathways. These findings highlight the potential of targeting VE-cadherin Y685 phosphorylation as a promising therapeutic approach to maintain retinal vascular integrity and attenuate the pathological progression of diabetic and prediabetic retinopathy.

## Abstract 2

Disrupting a harmful protein change protects retinal blood vessels in diabetes, reducing leakage and preventing loss of supportive cells that keep vessels stable and healthy.

## Introduction

Endothelial cells (ECs) in the retina are integral components of the blood-retinal barrier (BRB), forming blood vessels with pericytes to supply oxygen and nutrients to the metabolically active retina (1). Pericytes physiologically fully ensheathe ECs in the retina, providing structural support and regulating endothelial stability, permeability, and proliferation (2). The dropout of pericytes, the earliest vascular morphological change in diabetic retinopathy (DR), is attributed to an increase in retinal angiopoietin 2 (Ang2) (3–5). The lack of pericytes, followed by EC loss, leads to the formation of acellular capillaries (ACs), a distinctive pathological hallmark of retinal vasoregression.

Disruption of the pericyte-EC interaction is central to the pathogenesis of DR. ECs are essential for maintaining vascular homeostasis but are highly vulnerable to pathological stimuli such as hyperglycemia, contributing to the progression of the disease. Consequently, ECs are primary targets in the early stages of DR. ECs maintain the integrity of the BRB by regulating intercellular junction proteins, including vascular endothelial cadherin (VE-cadherin), a critical protein that supports vascular integrity (6, 7). The cytoplasmic domain of VE-cadherin contains tyrosine residues, such as Y685, Y658, and Y731, whose phosphorylation can trigger VE-cadherin internalization and increased endothelial permeability (8). Phosphorylation of VE-cadherin at Y685 functions as a molecular switch that selectively regulates vascular permeability to fluids while preserving leucocyte extravasation (9). This modification is triggered by permeability-promoting mediators such as vascular endothelial growth factor (VEGF) and plays a key role in fine-tuning endothelial barrier dynamics, particularly within the venous vasculature (10). A study by Wessel et al. has demonstrated that phosphorylation at Y685 specifically disrupts endothelial junctions and enhances vascular permeability (9). High glucose (HG) reduces the expression of VE-cadherin and impairs its mediated signaling, leading to the disruption of endothelial cell-cell adhesion (11, 12).

In the diabetic retina, hyperglycemia drives increased flux through the hexosamine biosynthesis pathway (HBP), a glycolytic branch that converts approximately 2%–5% of intracellular glucose into UDP-N-acetyl glucosamine (UDP-GlcNAc) via the rate-limiting enzyme glutamine:fructose-6-phosphate amidotransferase (GFAT) (13, 14). UDP-GlcNAc serves as a substrate for the O-GlcNAc cycle, including O-GlcNAc transferase (OGT) and O-GlcNAcase (OGA), enzymes that mediate protein modification through O-GlcNAcylation. OGT catalyzes the addition of GlcNAc to serine and threonine residues of proteins, while OGA facilitates its removal (15). This modification substantially affects protein function, and enhanced HBP activity is implicated in the pathogenesis of diabetic complications, including DR (16). Inhibition of GFAT and OGT, or induction of OGA, mitigates HG-induced retinal and endothelial damage (17, 18). Increased O-GlcNAcylation under HG conditions elevates Ang2 expression in ECs, initiating retinal abnormalities in DR (19).

Emerging evidence suggests that retinopathy precedes the diagnosis of diabetes, manifesting during the prediabetic stage (20–22). Similar pathogenic mechanisms underlying DR are presumed to contribute to prediabetic retinopathy (20). Nucleoside diphosphate kinases (NDPKs) are ubiquitous enzymes regulating intracellular nucleoside triphosphate pools. NDPKB-deficient (NDPKB^–/–^) mice exhibit impaired glucose tolerance, pericyte dropout, and increased ACs in the retina (23). Additionally, similar to DR, activation of the HBP, increased O-GlcNAc cycling, and elevated Ang2 expression are evident in NDPKB^–/–^ retinas and ECs, suggesting that NDPKB^–/–^ mice are an appropriate model of prediabetic retinopathy (24).

Despite substantial progress in understanding the role of endothelial HBP in diabetic and prediabetic retinopathy, and the main contribution of VE-cadherin phosphorylation to endothelial function, it remains unclear whether phosphorylation of VE-cadherin at 685 is a pivotal factor. This study aims to elucidate the link between VE-cadherin phosphorylation and HBP activation and retinal endothelial damage in diabetic and prediabetic retinopathy.

## Results

### VE-cadherin expression decreases not only in diabetic but also in prediabetic retinas.

**Figure 1:**
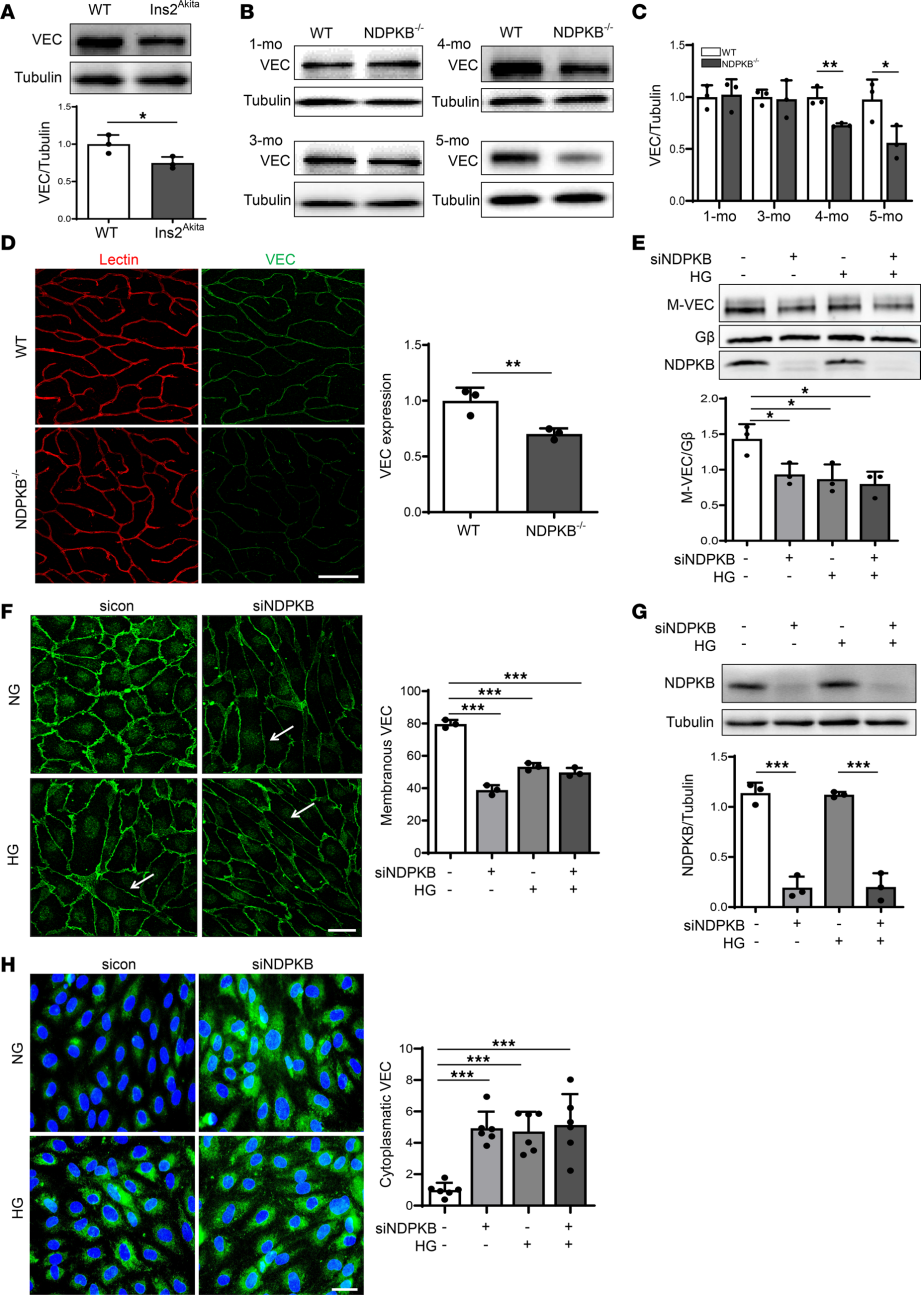
VE-cadherin expression is reduced in the diabetic and prediabetic retinas. (**A**) VE-cadherin expression was detected in the retinas of 6-week-old Ins2^Akita^ and control mice by immunoblotting. *n* = 3. (**B**) VE-cadherin expression in the retinas of NDPKB^–/–^ and WT mice of different ages (1, 3, 4, and 5 months) was determined using immunoblotting analysis. (**C**) Quantification of Western blot data. *n* = 3. (**D**) Retinal deep capillary layers were visualized with lectin (red) and VE-cadherin (green). *n* = 3. (**E**) HUVECs were transfected with either NDPKB siRNA or scrambled siRNA and stimulated with either NG (5.5 mM) or HG (30 mM). Gβ serves as a housekeeping marker on the cell membrane. *n* = 3. Overall *P* = 0.0119. (**F**) HUVECs were stained with VE-cadherin (green) to show cellular VE-cadherin distribution. Arrows show the linear changes, and membrane VE-cadherin expression was quantified. *n* = 3. Overall *P* < 0.001. (**G**) NDPKB knockdown efficiency was detected. *n* = 3. Overall *P* < 0.001. (**H**) Staining and quantification of internalized VE-cadherin in NDPKB-depleted HUVECs stimulated with HG. *n* = 6, repeated 3 times. Overall *P* = 0.0432. WT, wild-type mice; NDPKB^–/–^, NDPKB homozygous mice; NG, normal glucose; HG, high glucose; sicon, control siRNA; siNDPKB, NDPKB siRNA. *P < 0.05, **P < 0.01, ***P < 0.001. Statistical significance was determined using an unpaired 2-tailed *t* test (**A**, **C**, and **D**), or 1-way ANOVA with Tukey’s post hoc test for multiple comparisons (**E**–**H**). Scale bar: 50 μm.

Given the role of VE-cadherin in maintaining the integrity of the BRB, we first explored the impact of VE-cadherin on the progression of retinal vascular damage in 2 HBP-activated murine models: the Ins2 Akita mutation (Ins2^Akita^) mouse, a well-established model of DR, and the NDPKB^–/–^ mouse, a recently identified model of prediabetic retinopathy (23, 25, 26). In 6-week-old diabetic Ins2^Akita^ mice, a significant reduction in retinal VE-cadherin expression was detected in immunoblotting experiments (Figure 1A). NDPKB^–/–^ mice exhibited VE-cadherin expression levels comparable to controls at 1 and 3 months of age, but a significant decline was observed from 4 months onward, with a further reduction at 5 months (Figure 1, B and C). Whole-mount immunofluorescence staining of 4-month-old NDPKB^–/–^ and wild-type (WT) retinas confirmed the decrease in VE-cadherin expression, which was specifically localized to the deep capillary layer (Figure 1D), while retinal arteries and veins remained unaffected (Supplemental Figure 1, A–C; supplemental material available online with this article; https://doi.org/10.1172/JCI195048DS1). The NDPKB deficiency was confirmed via immunoblotting (Supplemental Figure 2).

Since membrane VE-cadherin is crucial for maintaining intercellular adhesion, we further examined the expression profile of membranous VE-cadherin in HG-treated and NDPKB-depleted ECs. We silenced NDPKB using siRNA in human umbilical vein endothelial cells (HUVECs) and exposed them to HG for 24 hours. Cellular fractionation followed by immunoblotting was conducted to detect VE-cadherin levels specifically in the membrane fraction. As shown in Figure 1E, with Gβ serving as a membrane marker, quantification revealed a significant downregulation of membranous VE-cadherin in both HG-treated and NDPKB-depleted ECs compared with control cells. However, the combination of these 2 conditions did not further alter the membranous VE-cadherin levels. To corroborate this finding, we performed immunofluorescence staining under the formerly mentioned conditions in ECs. In HG-treated and NDPKB-depleted ECs, VE-cadherin exhibited a disrupted, intermittent membrane pattern, in contrast with the continuous, zipper-like distribution seen in control cells (Figure 1F). A quantitative assessment of membrane immunofluorescence intensity by pixel density revealed an approximate 50% reduction in membranous VE-cadherin upon NDPKB depletion (Figure 1G) and a 40% reduction under HG treatment, with no further alteration observed in the combined condition, corroborating the immunoblotting results (Figure 1F). To exclude the effect of high osmotic stress on VE-cadherin expression levels, we treated ECs with l-glucose as an osmotic control. High osmotic stress induced by l-glucose did not affect VE-cadherin levels, whereas d-glucose markedly reduced them (Supplemental Figure 3, A–C).

Given the observed reduction in membranous VE-cadherin, we conducted immunofluorescence imaging to determine whether HG or NDPKB deficiency promotes VE-cadherin internalization. In non-acid-washed cells, VE-cadherin was localized at cell junctions. Following acid washing, which removes antibodies bound to membrane-located VE-cadherin, internalized VE-cadherin was detected in the cytosol, indicating its internalization (10). As shown in Figure 1H, enhanced VE-cadherin internalization was observed. ECs treated with VEGF served as a positive control, triggering rapid VE-cadherin internalization (data not shown). Both HG treatment and NDPKB deficiency significantly increased VE-cadherin internalization, comparable to the positive control. Notably, there was no further enhancement of VE-cadherin internalization in NDPKB-depleted ECs treated with HG (Figure 1H). The efficiency of NDPKB knockdown was verified by immunoblotting (data not shown).

These data suggest that VE-cadherin levels decline not only under diabetic but also in NDPKB deficiency–induced prediabetic retinopathy. HG exposure and NDPKB deficiency markedly reduce membranous VE-cadherin and promote its internalization, thereby compromising endothelial barrier function.

### The reduction of VE-cadherin in NDPKB^–/–^ mice precedes retinal vascular morphological changes.

**Figure 2:**
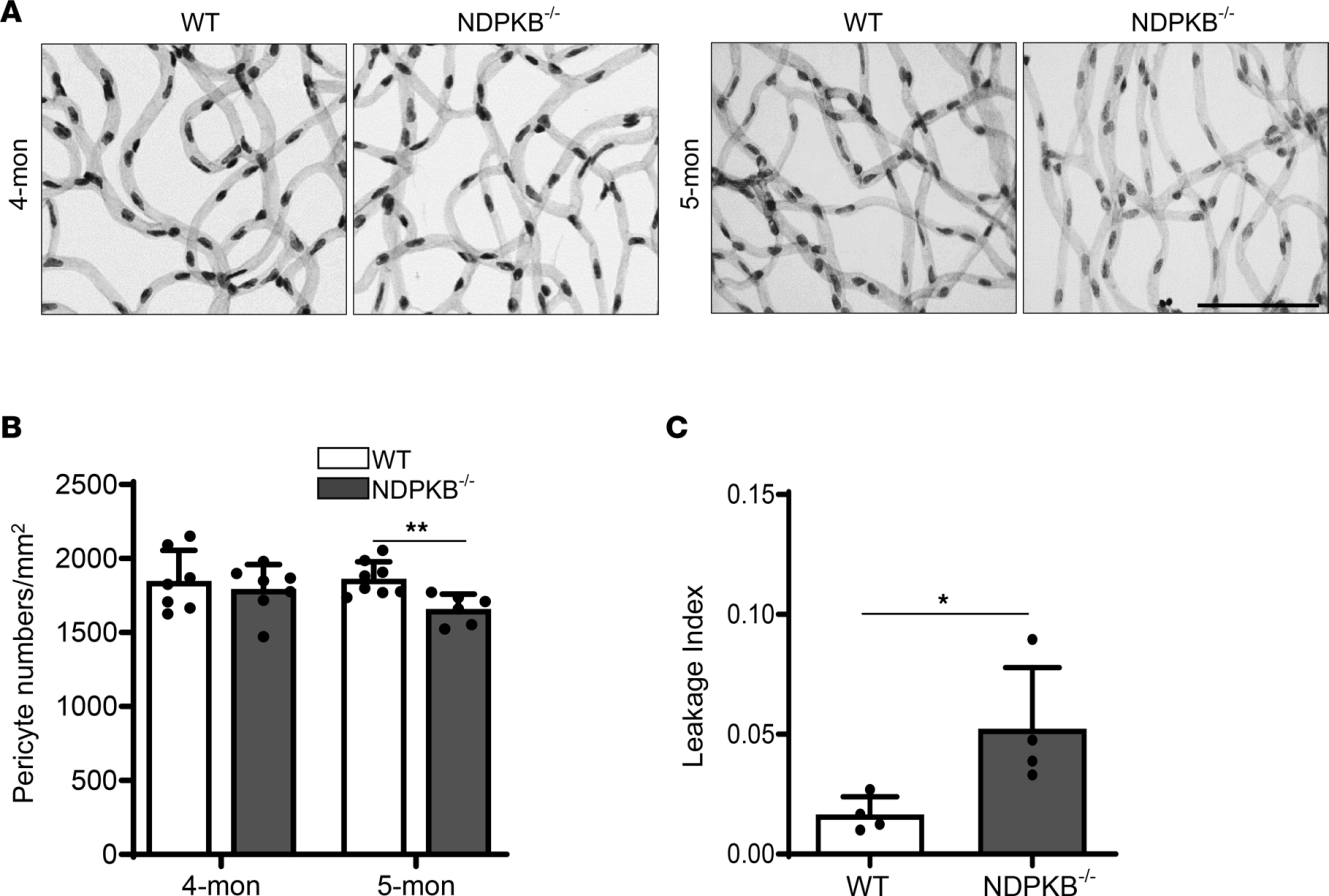
Vascular hyperpermeability and pericyte loss were observed in 5-month-old NDPKB^–/–^ retinas. (**A**) Representative images of retinal digestion and (**B**) quantification of retinal pericyte coverage using 4- and 5-month-old NDPKB^–/–^ retinas. *n* = 6–8. (**C**) Leakage index in 5-month-old NDPKB^–/–^ mice using intravenous injection of 70 kDa dextran-FITC. *n* = 4. WT, wild-type mice; NDPKB^–/–^, NDPKB homozygous mice. *P < 0.05, **P < 0.01. Statistical significance was determined by 2-tailed *t* test (**B**) and Mann-Whitney test (**C**). Scale bar: 50 μm.

To delineate whether VE-cadherin decline in NDPKB^–/–^ mice at 4 months occurs before or after morphological changes in the retinal vasculature, we conducted retina digestion on both 4- and 5-month-old NDPKB^–/–^ and WT mice, allowing visualization of the entire vasculature. This enabled us to pinpoint the onset of retinal morphological changes by analyzing pericyte loss in NDPKB^–/–^ retinas. Pericyte occupation in capillary areas, which mainly accrue from the deep retinal capillary layer, was estimated. At 4 months, despite a reduction in VE-cadherin levels, no significant difference in pericyte coverage was observed. However, by 5 months, NDPKB^–/–^ mice exhibited significant pericyte loss compared with WT mice (Figure 2, A and B). These findings suggest that VE-cadherin reduction in the retinal deep capillary layer might be an early indicator of retinopathy, preceding pericyte loss. To investigate whether increased retinal vascular permeability exists in prediabetic NDPKB^–/–^ mice, we evaluated the retinal endogenous albumin expression after saline perfusion in both NDPKB^–/–^ and WT mice at 5 months. Immunoblotting data demonstrated that retinal endogenous albumin expression in NDPKB^–/–^ retinas was substantially higher than in controls (Supplemental Figure 4). Since the albumin-based permeability assay has inherent limitations, we further performed an in vivo dextran-FITC leakage assay to validate our findings. Consistent with these findings, 5-month-old NDPKB-deficient mice exhibited a significantly elevated retinal leakage index compared with WT controls (Figure 2C). To visualize the leakage site, we analyzed 11-month-old NDPKB^–/–^ mice and found that dextran extravasation localized predominantly to perivenular/capillary regions (Supplemental Figure 5). The hyperpermeability of retinal vessels in NDPKB^–/–^ mice might be a result of the reduction in retinal VE-cadherin expression. The data suggest that the reduction of VE-cadherin is involved in vascular hyperpermeability and extravasation of albumin into the retinal tissue, reflecting a breakdown of the BRB.

### Phosphorylation of VE-cadherin at Y685 is triggered by HG and NDPKB deficiency, likely preceding protein O-GlcNAcylation and Ang2 upregulation.

**Figure 3:**
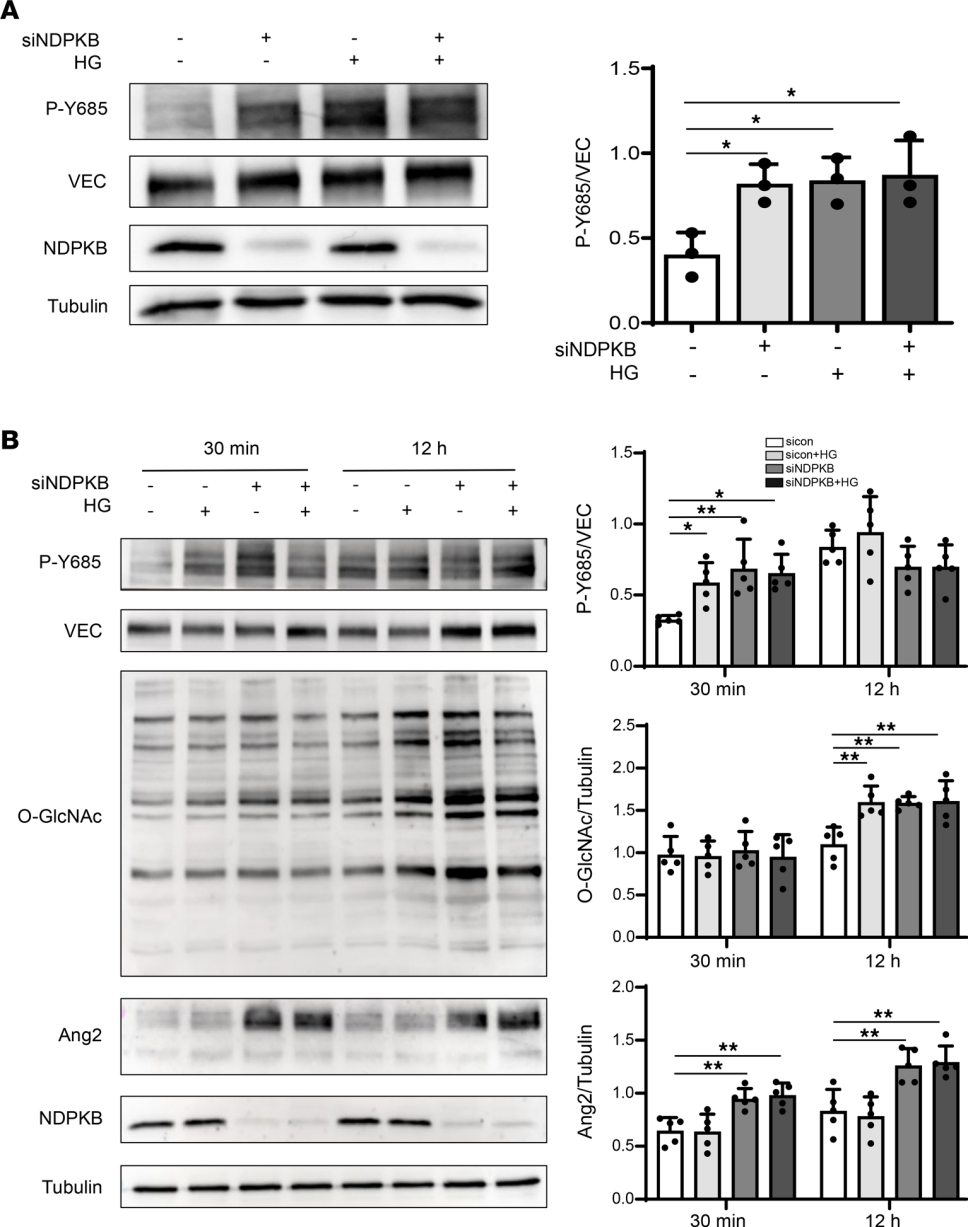
VE-cadherin Y685 phosphorylation is triggered by HG and NDPKB deficiency, prior to elevation of protein O-GlcNAcylation and Ang2. (**A**) Representative immunoblots of HUVECs under both HG and NDPKB-deficient conditions and quantitation of Y685 expression in NDPKB-depleted HUVECs with or without HG treatment for 24 hours. *n* = 3. Overall *P* = 0.0149. (**B**) Representative immunoblots of protein expression in NDPKB-depleted HUVECs treated with or without HG. Quantitation of Y685 (30 minutes overall *P* = 0.0042; 12 hours overall *P* = 0.1136), O-GlcNAc (30 minutes overall *P* = 0.947; 12 hours overall *P* = 0.0011), and Ang2 (30 minutes overall *P* < 0.001; 12 hours overall *P* < 0.001) expression in NDPKB-depleted HUVECs with or without HG treatment after 30 minutes and 12 hours. *n* = 5. HG, high glucose; VEC, VE-cadherin; siNDPKB, NDPKB siRNA. *P < 0.05, **P < 0.01. Statistical significance was determined by 1-way ANOVA with Tukey’s post hoc test for multiple comparisons.

Phosphorylation of VE-cadherin at key tyrosine residues, including Y731, Y658, and Y685, plays distinct roles in regulating endothelial function. To determine whether HG and NDPKB deficiency induce VE-cadherin phosphorylation, we performed immunoblotting analyses using an appropriate p-Y685 antibody (Supplemental Figure 6). While VEGF stimulation rapidly induced VE-cadherin phosphorylation at Y685 within 15 minutes (Supplemental Figure 7), both HG and NDPKB depletion markedly enhanced phosphorylation of VE-cadherin at Y685 after 30 minutes, and no additive effect was observed in combined conditions (Figure 3A). In contrast, no significant changes in phosphorylation were observed at the Y658 and Y731 sites under these conditions (Supplemental Figure 8). These results suggest that phosphorylation at Y685 may be a critical regulatory event for VE-cadherin function in response to HG and NDPKB deficiency.

Our previous studies have shown that HG and NDPKB deficiency increase protein O-GlcNAcylation and Ang2 expression in ECs (27). To determine whether Y685 phosphorylation occurs prior to protein O-GlcNAcylation and Ang2 regulation, we performed temporal analysis of HG exposure and NDPKB depletion. Immunoblotting results demonstrated that Y685 phosphorylation was considerably induced after 30 minutes of HG stimulation or NDPKB depletion, with similar effects observed under combined conditions, consistent with previous findings in Figure 3A. At this point for 30 minutes, no significant changes in protein O-GlcNAcylation or Ang2 expression were detected under HG condition compared with the corresponding time-matched controls (Figure 3B). Basal Y685 phosphorylation was elevated in the sham condition, which is likely attributable to increased cell density. Y685 phosphorylation was not altered compared with their respective 12-hour controls. In contrast, both NDPKB depletion and HG treatment resulted in increased protein O-GlcNAcylation, with sustained effects under combined conditions. Although Ang2 expression was elevated in NDPKB-depleted ECs compared with control ECs at 12-hour time point, HG treatment did not alter Ang2 expression (Figure 3B). No detectable changes in VE-cadherin Y685 phosphorylation, Ang2 expression, or global O-GlcNAcylation were observed at the additional time points examined (2 and 6 hours) in ECs stimulated with HG, consistent with the findings at 30 minutes and 12 hours (Supplemental Figure 9). In summary, phosphorylation of VE-cadherin at Y685 likely precedes O-GlcNAcylation and contributes to the subsequent upregulation of Ang2 under HG and NDPKB-deficient conditions.

### Y685F mutation of VE-cadherin impairs protein O-GlcNAcylation and Ang2 upregulation, VE-cadherin internalization, and pericyte loss in vitro.

We hypothesized that the observed alteration in vascular cells under HG and NDPKB-deficient conditions may be mediated by the phosphorylation of VE-cadherin at Y685. To test this hypothesis, ECs exposed to HG or NDPKB-deficient conditions were infected with either Y685-Mut or Y685-WT adenovirus. The green fluorescent protein (GFP) expression served as a marker of adenovirus transfection efficiency, and cells exhibiting over 60% green fluorescence were selected for further experimentation. As expected, Y685-Mut infection led to lower Y685 phosphorylation compared with Y685-WT (Supplemental Figure 10).

**Figure 4:**
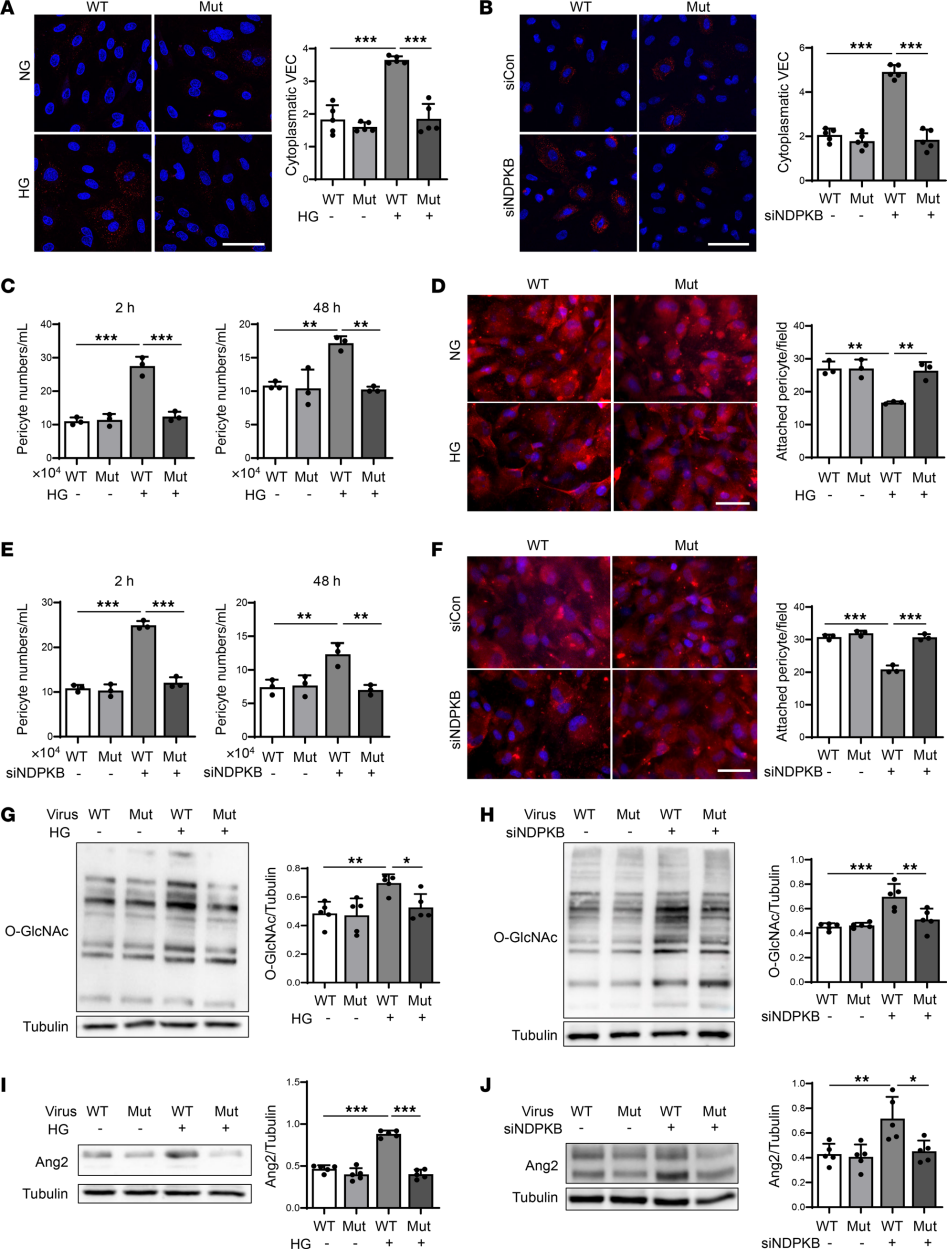
VE-cadherin Y685 phosphorylation impairs protein O-GlcNAcylation, Ang2 upregulation, VE-cadherin internalization, and pericyte loss in vitro. (**A**) Immunofluorescence images and quantification of internalized VE-cadherin in HUVECs infected with adenovirus WT or Mut under HG conditions. *n* = 5, repeated 3 times. Overall *P* < 0.001. (**B**) Immunofluorescence images and quantification of internalized VE-cadherin in NDPKB-depleted HUVECs infected with adenovirus WT or Mut. *n* = 5, repeated 3 times. Overall *P* < 0.001. Analysis of the nonattached or detached pericytes labeled with Tracker in Transwell contacting coculture with HUVECs infected with adenovirus WT or Mut for 2 hours or 48 hours under HG conditions (2 hours overall *P* < 0.001; 48 hours overall *P* = 0.0015) (**C**) and NDPKB deficiency (2 hours overall *P* < 0.001; 48 hours overall *P* = 0.0034) (**E**). *n* = 3. Images and quantification of attached pericytes labeled with Tracker on the HUVEC monolayer with WT or Mut adenovirus infection after 48 hours’ Transwell contacting coculture under HG conditions (overall *P* < 0.001) (**D**) and NDPKB deficiency (overall *P* < 0.001) (**F**). *n* = 3. Representative immunoblotting analysis and quantification of O-GlcNAc (overall *P* = 0.0045) (**G**) and Ang2 (overall *P* < 0.001) (**I**) in HG-treated HUVECs infected with adenovirus WT or Mut. *n* = 5. Representative immunoblotting analysis and quantification of O-GlcNAc (overall *P* < 0.001) (**H**) and Ang2 (overall *P* = 0.0023) (**J**) in NDPKB-depleted HUVECs infected with WT or Mut adenovirus. *n* = 5. WT, VE-cadherin Y685; Mut, VE-cadherin Y685F mutation; NG, normal glucose; HG, high glucose; sicon, control siRNA; siNDPKB, NDPKB siRNA. *P < 0.05, **P < 0.01, ***P < 0.001. Statistical significance was determined by 1-way ANOVA with Tukey’s post hoc test for multiple comparisons. Scale bar: 50 μm.

We first investigated the effect of Y685 phosphorylation on VE-cadherin internalization. As shown in Figure 4A, internalized VE-cadherin increased in Y685-WT infected ECs under HG treatment compared with normal glucose conditions. In contrast, Y685-Mut infection mitigated the internalized VE-cadherin induced by HG (Figure 4A). Likewise, NDPKB depletion resulted in VE-cadherin internalization in Y685-WT infected ECs, while Y685-Mut infection inhibited VE-cadherin internalization into the cytoplasm (Figure 4B). These data indicate that Y685 phosphorylation contributes to VE-cadherin dysfunction at the cell membrane, promoting its internalization under both HG and NDPKB-deficient conditions.

To assess EC-pericyte functional interactions, we performed EC-pericyte coculture assays. First, in the direct-contact coculture, under either HG or NDPKB knockdown, Y685-WT ECs exhibited increased pericyte detachment at 2 hours, as reflected by higher pericyte numbers in the supernatant, whereas Y685-Mut ECs maintained early adhesion. The differences diminished from 4 to 10 hours, but by 24 hours, Y685-WT monolayers exhibited greater pericyte loss and Y685-Mut preserved pericyte adhesion to ECs. Quantification of adherent pericytes on the endothelial layer paralleled the supernatant counts (Supplemental Figure 11). To more closely approximate the physiological juxtaposition of ECs and pericytes on the vascular basement membrane, we conducted a Transwell HUVEC-pericyte contacting coculture system using inverted chamber inserts. Endothelial monolayers were grown on the abluminal surface of the porous membrane, while Tracker-labeled pericytes were seeded on the luminal side. At 2 hours, HG increased the number of nonadherent pericytes in cocultures with Y685-WT ECs, whereas Y685-Mut prevented this defect, maintaining levels comparable to controls (Figure 4C). No differences were detected at 24 hours (Supplemental Figure 12A). However, by 48 hours, HG elevated detached pericytes in the Y685-WT condition, while Y685-Mut continued to preserve pericyte attachment (Figure 4C). Imaging the labeled pericytes on the membranes at 48 hours confirmed the finding. HG reduced the number of attached pericytes on Y685-WT endothelial monolayers, whereas Y685-Mut maintained pericyte coverage (Figure 4D). Under NDPKB depletion, a similar pattern emerged, with Y685-Mut preserving pericyte attachment despite loss of NDPKB (Figure 4, E and F, and Supplemental Figure 12B). To sum up, these findings demonstrate that HG and NDPKB deficiency impair EC-pericyte interactions by reducing adhesion and promoting detachment, whereas the Y685 mutant consistently sustains pericyte attachment.

To assess whether HBP activation and Ang2 elevation are downstream effectors regulated by Y685, we measured protein O-GlcNAcylation and Ang2 levels. Y685-WT infection exacerbated HG-induced HBP activation and Ang2 production, whereas Y685-Mut infection mitigated these effects (Figure 4, G and I). To explore whether NDPKB deficiency similarly triggers these changes via Y685 phosphorylation, we performed immunoblotting, and the results demonstrated that NDPKB depletion markedly increased protein O-GlcNAcylation and Ang2 levels in Y685-WT ECs, while these effects were reduced under the Y685-Mut condition, as observed under HG treatment (Figure 4, H and J).

Together, these in vitro results provide compelling evidence that Y685 phosphorylation plays a crucial role in HG- and NDPKB deficiency–induced EC dysfunction by activating the HBP and increasing Ang2 expression. This process triggers VE-cadherin internalization and leads to impaired pericyte coverage. Further in vivo experiments with Y685 phosphorylation mutants are necessary to validate this hypothesis.

### O-GlcNAcylation mediates VE-cadherin Y685-dependent Ang2 upregulation under HG and NDPKB-deficient conditions.

**Figure 5:**
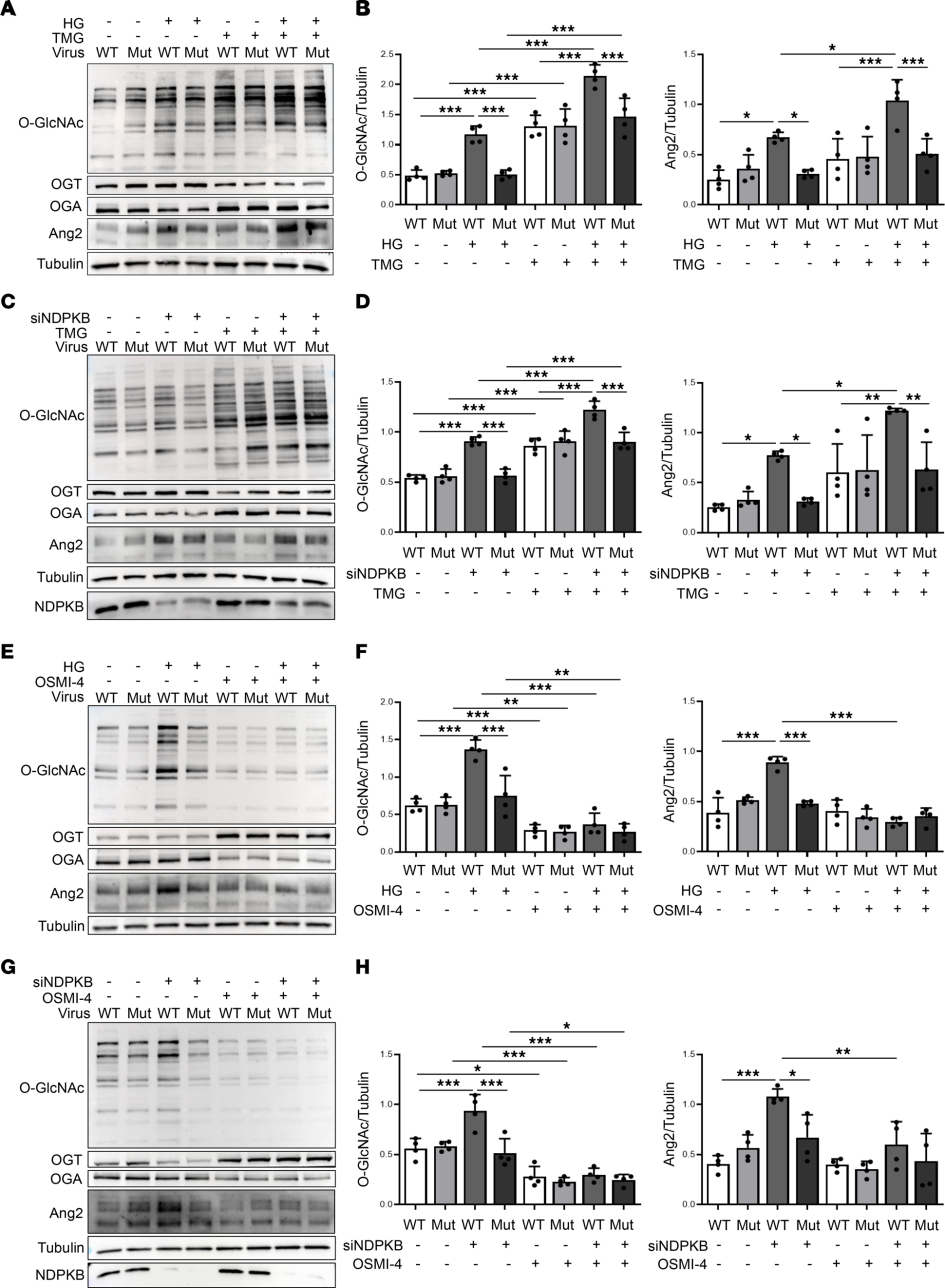
O-GlcNAcylation mediates VE-cadherin Y685-dependent Ang2 upregulation under HG and NDPKB-deficient conditions. Representative immunoblotting of O-GlcNAc, OGT, OGA, and Ang2 in HG-treated HUVECs (**A**) and NDPKB-depleted HUVECs (**C**) infected with adenovirus WT or Mut, combined with TMG treatment. Quantification of O-GlcNAc and Ang2 expression in HG-treated HUVECs (**B**) and NDPKB-depleted HUVECs (**D**). Representative immunoblotting analysis of O-GlcNAc, OGT, OGA, and Ang2 in HG-treated HUVECs (**E**) and NDPKB-depleted HUVECs (**G**) infected with adenovirus WT or Mut, combined with OSMI-4 treatment. Quantification of O-GlcNAc and Ang2 expression in HG-treated HUVECs (**F**) and NDPKB-depleted HUVECs (**H**). *n* = 4. WT, VE-cadherin Y685; Mut, VE-cadherin Y685F mutation; HG, high glucose; siNDPKB, NDPKB siRNA; TMG, Thiamet G; OSMI-4, O-GlcNAc transferase inhibitor 4. *P < 0.05, **P < 0.01, ***P < 0.001. Statistical significance was determined by 1-way ANOVA with Tukey’s post hoc test for multiple comparisons. All overall *P* < 0.001.

Building on our observation that HG activates the HBP and raises Ang2 levels, we next investigated the contribution of O-GlcNAc to VE-cadherin Y685-dependent Ang2 upregulation. ECs expressing either Y685-WT or Y685-Mut were exposed to pharmacological modulators of O-GlcNAc cycling. Treatment with the OGA inhibitor, Thiamet G (TMG), increased O-GlcNAcylation in both genotypes and produced a modest compensatory shift in the cycling enzymes, with lower OGT and higher OGA protein levels (Supplemental Figure 13, A and B). TMG augmented HG-induced Ang2 in Y685-WT cells but not in Y685-Mut cells (Figure 5, A and B). Knockdown of NDPKB increased global O-GlcNAcylation and Ang2 in Y685-WT cells, but not in Y685-Mut cells, and TMG further enhanced O-GlcNAcylation in both but selectively increased Ang2 in Y685-WT cells. Y685-Mut cells maintained Ang2 at low levels (Figure 5, C and D). At the same time, pharmacological inhibition of OGT using OGT inhibitor 4 (OSMI-4) reduced global O-GlcNAcylation, with compensative higher OGT and lower OGA protein levels (Supplemental Figure 13, C and D). OSMI-4 abolished the HG-induced increase in Ang2 in Y685-WT cells, returning Ang2 to baseline levels indistinguishable from those in Y685-Mut cells (Figure 5, E and F). Similarly, OSMI-4 suppressed the increase in Ang2 elicited by NDPKB knockdown in Y685-WT cells, reducing Ang2 to baseline levels (Figure 5, G and H).

Enhancement of O-GlcNAcylation with TMG or its inhibition with OSMI-4, together with HG or NDPKB deficiency, showed that Ang2 upregulation requires both VE-cadherin Y685 phosphorylation and elevated O-GlcNAcylation. Thus, O-GlcNAcylation mediates VE-cadherin Y685-dependent Ang2 upregulation under HG and NDPKB-deficient conditions.

### Suppression of VE-cadherin Y685 phosphorylation protects retinal pathology.

**Figure 6:**
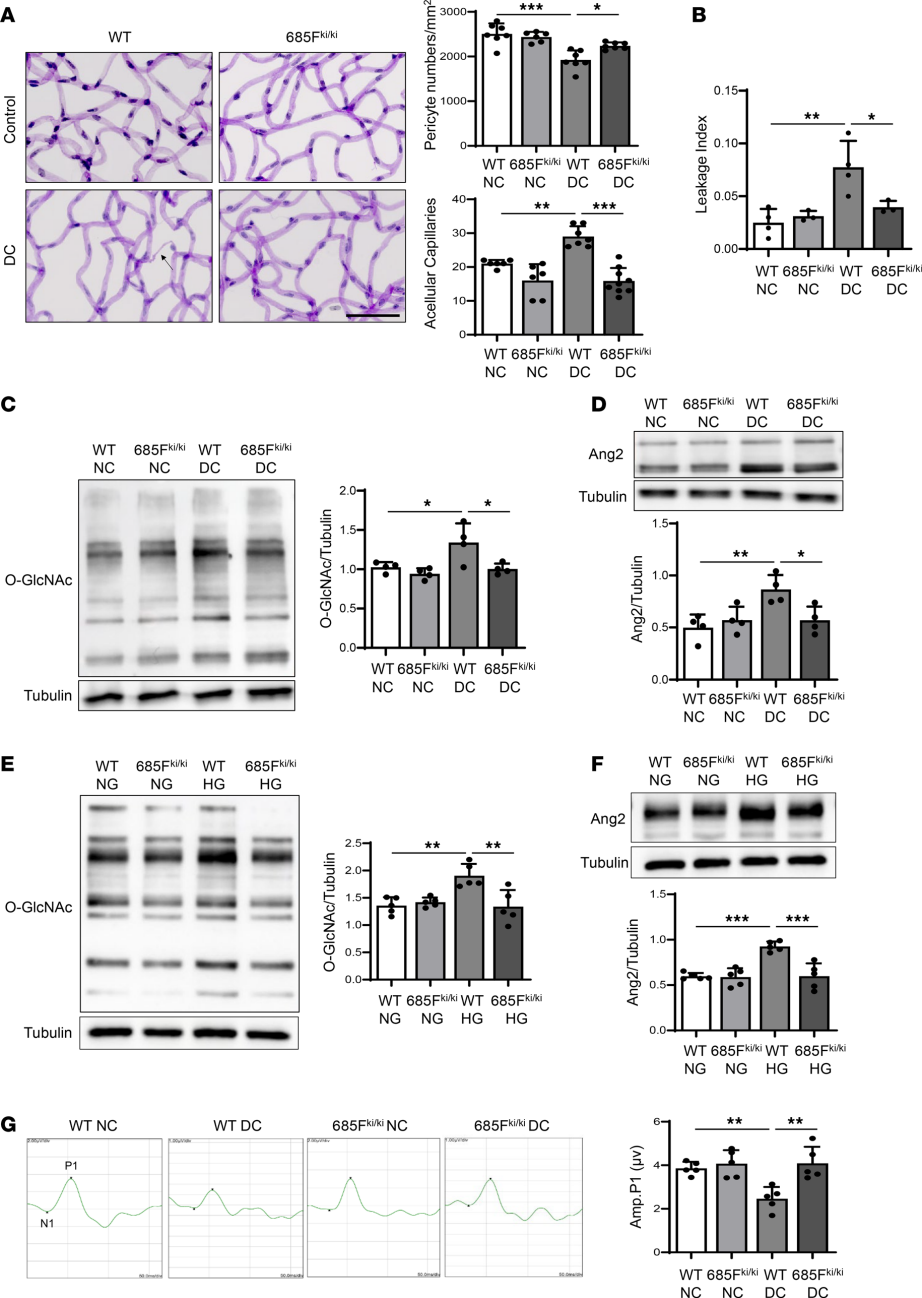
Suppressing VE-cadherin Y685 phosphorylation reverses HBP activation and retinal damage in DR. (**A**) Assessment of retinal vascular damage by retinal digestion and the quantification of pericyte coverage (overall *P* < 0.001) and ACs (overall *P* < 0.001) in 6-month diabetic Y685F^ki/ki^ mice. Arrow shows an AC. *n* = 6–9. (**B**) Leakage index using dextran-FITC in 3-month diabetic WT and diabetic VEC Y685F^ki/ki^ retinas. *n* = 3–4. Overall *P* = 0.0039. Immunoblots and quantitation of retinal O-GlcNAc (overall *P* = 0.0055) (**C**) and Ang2 (overall *P* = 0.0088) (**D**) in diabetic VEC Y685F^ki/ki^ retinas. *n* = 4. Representative immunoblots of O-GlcNAc (overall *P* = 0.0013) (**E**) and Ang2 (overall *P* < 0.001) (**F**) in HG-treated MBMECs isolated from VEC Y685F^ki/ki^ mice. *n* = 5. (**G**) Tracing and quantification of P1 wave in multifocal electroretinography (ERG) of 3-month diabetic Y685F^ki/ki^ retinas. *n* = 5. Overall *P* = 0.001. NC, nondiabetic; DC, diabetic; WT, wild-type; 685F^ki/ki^, VE-cadherin Y685F knockin; NG, normal glucose; HG, high glucose. *P < 0.05, **P < 0.01, ***P < 0.001. Statistical significance was determined by 1-way ANOVA with Tukey’s post hoc test for multiple comparisons. Scale bar: 50 μm.

Since Y685 phosphorylation has been identified as a critical factor driving EC dysfunction in hyperglycemia-induced retinopathy, as demonstrated in vitro, we further explored whether targeting this phosphorylation site could offer a potential interventional strategy for retinopathy associated with hyperglycemic conditions. To investigate this, we utilized a VE-cadherin Y685F-knockin (VEC Y685F^ki/ki^) mouse model and induced DR by administering streptozotocin (STZ) to both VEC Y685F^ki/ki^ and WT mice. We evaluated pericyte coverage and AC numbers to assess the impact of Y685F mutation on retinal vasculature. As shown in Figure 6A, pericyte coverage was markedly reduced in diabetic (DC) WT mice compared with nondiabetic (NC) WT mice. No difference in pericyte coverage was observed between NC VEC Y685F^ki/ki^ and WT retinas. Notably, DC VEC Y685F^ki/ki^ retinas exhibited higher pericyte coverage compared with DC WT mice, suggesting that the Y685F mutation mitigates vascular damage induced by hyperglycemia. Additionally, no significant differences were observed between NC and DC VEC Y685F^ki/ki^ mice. Similarly, AC numbers inversely followed changes in pericyte coverage, being elevated in DC WT mice relative to NC WT mice, indicating a greater loss of pericytes and ECs under hyperglycemia. As pericyte coverage, AC numbers were comparable between NC VEC Y685F^ki/ki^ and WT mice. In contrast, DC VEC Y685F^ki/ki^ mice exhibited fewer ACs than DC WT mice, further supporting the hypothesis that the Y685F mutation protects retinal vessels against vascular damage in hyperglycemic conditions (Figure 6A).

Considering that vascular permeability is compromised in DR (28) and the integrity of the BRB depends on the maintenance of cell-to-cell junctions in the retinal vascular endothelium, we evaluated retinal vascular permeability in DC VEC Y685F^ki/ki^ mice. DC WT mice showed a 3-fold increase in albumin levels in the retina compared with NC WT mice. No significant difference was observed between NC VEC Y685F^ki/ki^ and WT mice. Importantly, VEC Y685F^ki/ki^ mice exhibited substantial protection from hyperpermeability, with no apparent difference in retinal albumin leakage between DC and NC VEC Y685F^ki/ki^ mice. Similarly, DC VEC Y685F^ki/ki^ mice showed reduced albumin leakage compared with DC WT mice (Supplemental Figure 14). Consistent with the albumin-based results, we observed comparable hyperpermeability changes. Quantification showed a marked increase in vascular leakage in DC WT retinas compared with NC WT. In contrast, DC VEC Y685F^ki/ki^ retinas were indistinguishable from NC VEC Y685F^ki/ki^, exhibiting substantially lower dextran-FITC leakage than DC WT (Figure 6B). These data suggest that the vascular protection observed in the VEC Y685F^ki/ki^ mice is likely due to the inhibition of vascular hyperpermeability.

To elucidate the underlying mechanisms responsible for the protective effect in VEC Y685F^ki/ki^ mice, we assessed retinal protein O-GlcNAcylation and Ang2 expression. Protein O-GlcNAcylation was nearly doubled in DC WT retinas compared with NC controls, while no significant differences were observed between NC VEC Y685F^ki/ki^ and WT retinas. Remarkably, protein O-GlcNAcylation levels were markedly reduced in DC VEC Y685F^ki/ki^ mice compared with DC WT mice, effectively abolishing the hyperglycemia-induced increase. No difference was observed between DC and NC VEC Y685F^ki/ki^ mice. These results highlight the critical role of Y685 phosphorylation in hyperglycemia-induced HBP activation (Figure 6C). We further examined Ang2 expression using an appropriate Ang2 antibody for semiquantitative analysis (Supplemental Figure 15). In line with our previous findings, the Ang2 levels were elevated in DC WT mice in comparison with NC WT mice. Ang2 expression exhibited a pattern that closely paralleled the changes observed in protein O-GlcNAcylation across the 4 experimental groups. Notably, the Y685F mutation suppressed Ang2 upregulation in DC VEC Y685F^ki/ki^ mice (Figure 6D).

To further delineate the endothelium-specific contribution to the protective effects observed in VEC Y685F^ki/ki^ mice, we isolated murine brain microvascular endothelial cells (MBMECs) from VEC Y685F^ki/ki^ mice and exposed them to HG. Consistent with the retinal findings in VEC Y685F^ki/ki^ mice, HG treatment substantially increased protein O-GlcNAcylation and Ang2 expression in WT MBMECs. No differences were observed between Y685F^ki/ki^ and WT MBMECs under normal glucose conditions. HG-induced increase in protein O-GlcNAcylation and Ang2 expression were abolished in MBMECs isolated from VEC Y685F^ki/ki^ mice, further reinforcing the central role of Y685 phosphorylation in mediating retinal vascular damage (Figure 6, E and F).

In addition to vascular damage, DR is characterized by the early onset of neuronal dysfunction (29). Therefore, we assessed neuronal function in VEC Y685F^ki/ki^ retinas using electroretinography (ERG). As shown in Figure 6G, alterations in P1 wave amplitude were observed. Specifically, DC WT mice exhibited a nearly 50% reduction in P1 wave amplitude compared with NC WT mice, indicating impaired inner retinal function. NC VEC Y685F^ki/ki^ mice exhibited similar wave patterns and amplitudes as NC WT mice. However, DC VEC Y685F^ki/ki^ mice showed considerably higher P1 wave amplitudes than DC WT mice, suggesting that the Y685F mutation effectively prevents hyperglycemia-induced neuronal damage. No significant differences in P1 wave amplitudes were observed between NC and DC VEC Y685F^ki/ki^ mice (Figure 6G). In addition, N1 wave amplitudes remained unchanged across all 4 groups, indicating unaffected photoreceptor function (Supplemental Figure 16). Additionally, the Müller cell activation marker, glial fibrillary acidic protein, was elevated in DC WT retinas compared with control retinas but normalized in DC VEC Y685^ki/ki^ retinas (Supplemental Figure 17). These findings suggest that improved vascular function and glial activation resulting from suppression of Y685 phosphorylation protects retinal neuronal function in diabetic retinas.

**Figure 7:**
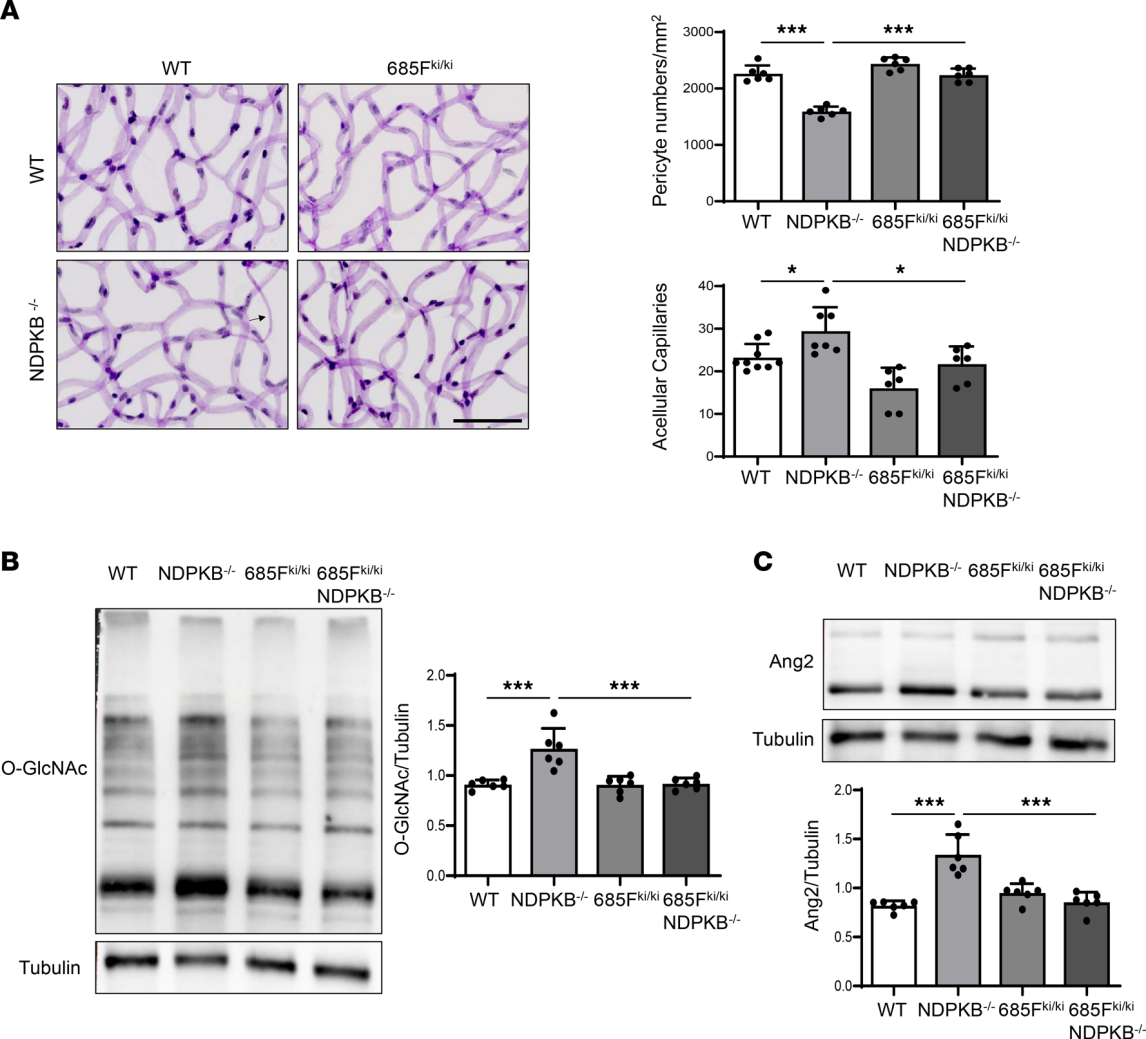
Suppressing VE-cadherin Y685 phosphorylation reverses HBP activation and retinal damage in prediabetic retinopathy. (**A**) Estimation and quantification of pericyte coverage and ACs in VEC Y685F^ki/ki^ NDPKB^–/–^ double-transgenic retinas. The arrow shows an AC. *n* = 6–9. Immunoblots of retinal O-GlcNAc (**B**) and Ang2 (**C**) in 8-month-old VEC Y685F^ki/ki^ NDPKB^–/–^ double-transgenic mice. *n* = 6. WT, wild-type; 685F^ki/ki^, VE-cadherin Y685F knockin; NDPKB^–/–^, NDPKB homozygous. 685F^ki/ki^ NDPKB^–/–^, VEC Y685F^ki/ki^ NDPKB^–/–^ double transgenic mice. *P < 0.05, ***P < 0.001. Statistical significance was determined by 1-way ANOVA with Tukey’s post hoc test for multiple comparisons. All overall *P* < 0.001. Scale bar: 50 μm.

Given that a similar pathology of DR presents in the prediabetic NDPKB^–/–^ mice, we next evaluated the effects of the Y685F mutation under NDPKB-deficient conditions using VEC Y685F^ki/ki^ NDPKB^–/–^ mice. Retinal morphometry was assessed through retina digestion in WT, NDPKB^–/–^, VEC Y685F^ki/ki^, and Y685F^ki/ki^ NDPKB^–/–^ mice (Figure 7A). As expected, results consistent with those observed in diabetic mice were obtained: NDPKB^–/–^ mice exhibited appreciably reduced pericyte coverage and increased AC formation compared with WT controls. No differences were observed between VEC Y685F^ki/ki^ and WT mice. The Y685F mutation prevented the vascular damage observed in NDPKB^–/–^ mice, restoring normal pericyte coverage and AC numbers in Y685F^ki/ki^ NDPKB^–/–^ mice compared with NDPKB^–/–^ mice. NDPKB^–/–^ mice exhibited a 65% increase in albumin levels in the retina compared with WT mice. VEC Y685F displayed robust protection against hyperpermeability, as evidenced by the reduced albumin leakage in Y685F^ki/ki^ NDPKB^–/–^ mice when compared with NDPKB^–/–^ mice (Supplemental Figure 18). These findings suggest a vascular protective effect of VEC Y685F against hyperpermeability in prediabetic NDPKB^–/–^ mice.

Retinal protein expression analysis showed no significant difference in O-GlcNAcylation levels between VEC Y685F^ki/ki^ and WT mice. However, a 40% increase in protein O-GlcNAcylation was observed in NDPKB^–/–^ mice compared with WT controls. In contrast, protein O-GlcNAcylation levels were significantly reduced in VEC Y685F^ki/ki^ NDPKB^–/–^ mice compared with NDPKB^–/–^ mice, similar to those in VEC Y685F^ki/ki^ mice (Figure 7B). This pattern closely mirrored the results observed in DC VEC Y685F^ki/ki^ mice. Additionally, Ang2 levels exhibited a comparable pattern to protein O-GlcNAcylation, with increased expression in NDPKB^–/–^ mice relative to WT controls and an attenuation in VEC Y685F^ki/ki^ NDPKB^–/–^ mice (Figure 7C).

In summary, the overall protein changes observed align with our in vitro data, supporting the concept that hyperglycemia or NDPKB deficiency–induced VE-cadherin Y685 phosphorylation contributes to vascular damage in diabetic and prediabetic retinopathy. The VE-cadherin Y685F mutation mitigates vascular and neuronal damage in diabetic and prediabetic retinopathy.

### VE-cadherin Y685F-mediated mitigation of retinal pathology engages neurovascular and mitochondrial protein O-GlcNAcylation.

Principal component analysis (PCA) of the O-GlcNAc–enriched retinal proteome showed a clear diabetes-driven separation in WT samples. DC WT replicates diverged from NC WT along PC1, whereas NC and DC VEC Y685F^ki/ki^ clustered with NC WT (Supplemental Figure 19A). Volcano plots revealed extensive diabetes-induced remodeling in WT retinas (185 increased, 231 decreased in DC WT vs. NC WT). In contrast, NC VEC Y685F^ki/ki^ mice showed only modest baseline differences from NC WT (50 increased, 80 decreased). Under diabetic condition, however, the Y685F mutation differed strongly from WT, as DC VEC Y685F^ki/ki^ retinas exhibited 232 increased and 252 decreased proteins compared with DC WT. Diabetes still altered the VEC Y685F^ki/ki^ proteomes (109 increased, 142 decreased), though to a lesser extent than in WT. Overall, diabetes robustly remodeled O-GlcNAc landscape in WT retinas, while the Y685F mutation dampened these shifts within the Y685F^ki/ki^ cohort but resulted in strong Y685-specific divergence under diabetes (Supplemental Figure 19B and Supplemental Table 2).

**Figure 8:**
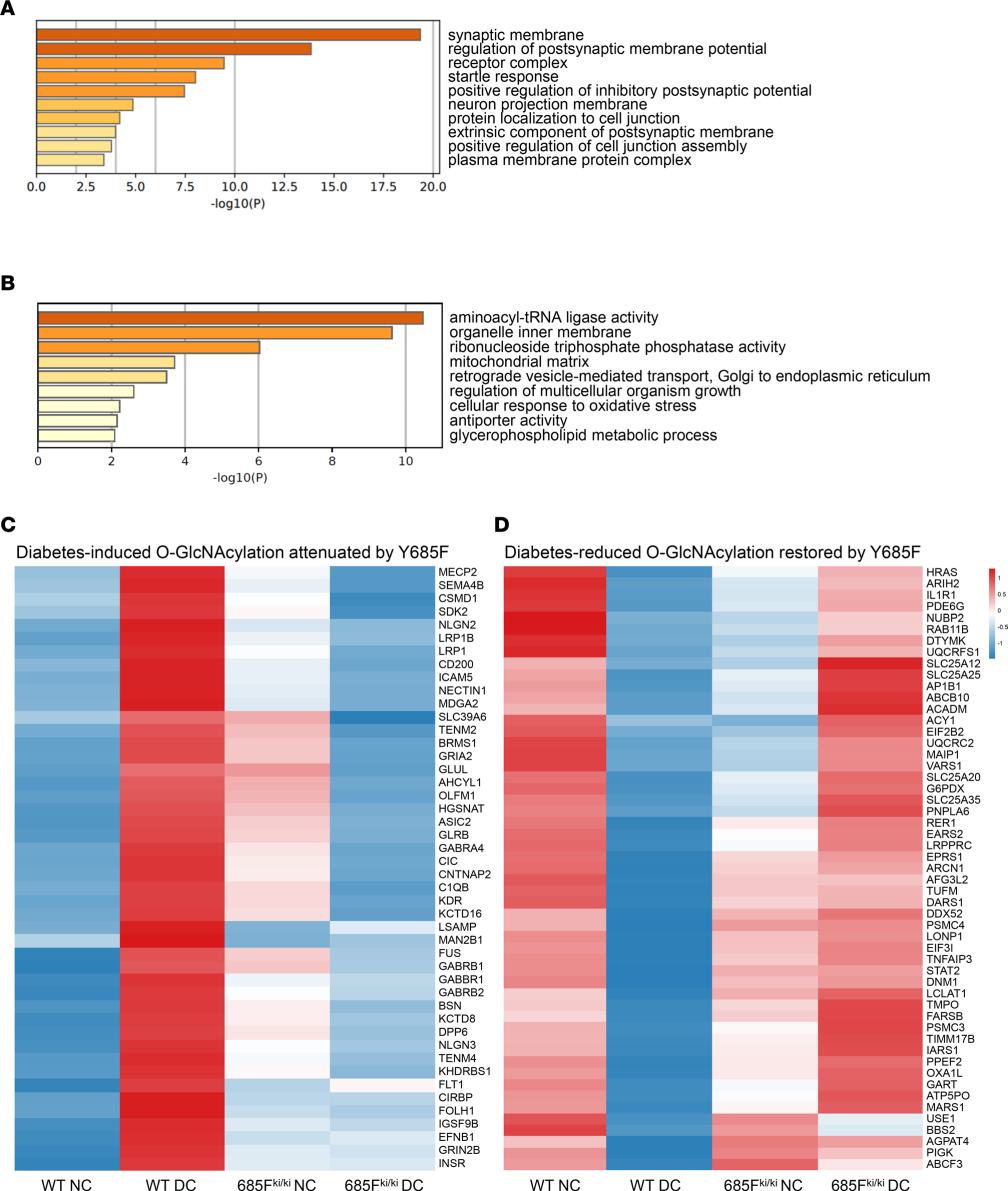
VE-cadherin Y685F-mediated reversal of retinal pathology engages neurovascular and mitochondrial protein O-GlcNAcylation. GO terms enriched among proteins that increased (**A**) or decreased (**B**) in 3-month DC WT vs. NC WT but showed opposite direction in DC VEC Y685F^ki/ki^ vs. DC WT. Heatmap of group mean *Z*-scores for proteins with increased (**C**) or decreased (**D**) O-GlcNAcylation in 3-month DC WT vs. NC WT but opposite direction in DC VEC Y685F^ki/ki^ vs. DC WT. Colors indicate relative O-GlcNAc abundance, blue (low) to red (high). NC, nondiabetic; DC, diabetic; WT, wild-type; 685F^ki/ki^, VE-cadherin Y685F knockin.

Gene ontology (GO) analysis revealed that proteins with increased O-GlcNAcylation in DC WT retinas but reduced in DC VEC Y685F^ki/ki^ retinas were strongly enriched in neuronal and receptor-related pathways, as well as at cell junctions (Figure 8A). In contrast, proteins with reduced O-GlcNAcylation in DC WT retinas but increased O-GlcNAcylation in DC VEC Y685F^ki/ki^ retinas were predominantly associated with mitochondrial matrix and inner membrane structures. Interestingly, pathways involved in protein synthesis and RNA metabolism were prominently altered as well (Figure 8B). Overall, these GO patterns suggest that diabetes elevates O-GlcNAcylation in neuronal and junction-associated pathways while suppressing mitochondrial processes in WT retinas, which are prevented by the Y685F mutation.

The *Z*-score heatmaps delineate 2 complementary, pathway-rich protein complexes. In DC WT retinas, O-GlcNAcylation increased coordinately in neurovascular and junction-associated proteins, including adhesion and membrane organizers (ICAM5, CD200, NECTIN1, LRP1/LRP1B, CSMD1, and SDK2), synaptic membrane proteins (CNTNAP2, NLGN2, NLGN3, TENM2, and TENM4), as well as VEGF receptors (KDR and FLT1) (Figure 8C and Supplemental Table 2). At the same time, O-GlcNAcylation decreased in mitochondrial programs, including proteostasis and biogenesis factors (LONP1, LRPPRC, and AFG3L2), import and assembly proteins (TIMM17B and OXA1L), metabolic and redox components (ACADM, SLC25A20 and G6PDX), respiratory chain components (UQCRC2 and UQCRFS1), transport and dynamics factors (ABCB10, SLC25A12, SLC25A25, and DNM1), and mitochondrial translation-related proteins (TUFM and EARS2) (Figure 8D and Supplemental Table 2). Notably, immune response-related proteins, including C1QB and STAT2, also showed pronounced alterations. These diabetes-associated changes in O-GlcNAcylation were largely reversed in DC VEC Y685F^ki/ki^ retinas, indicating that VE-cadherin Y685F-mediated protection of retinal pathology engages neurovascular and mitochondrial protein O-GlcNAcylation.

## Discussion

In this study, we demonstrate that alterations in VE-cadherin expression precede vascular pathological changes in diabetic and prediabetic retinopathy. Specifically, phosphorylation of VE-cadherin at Y685 mediates HBP activation, resulting in increased protein O-GlcNAcylation and enhanced Ang2 expression in ECs. Through pharmacological modulation of O-GlcNAcylation, we uncovered the essential role of O-GlcNAcylation in VE-cadherin Y685-dependent Ang2 upregulation. Notably, by using a knockin mouse model expressing a Y685F mutant of VE-cadherin, we show that interfering with VE-cadherin phosphorylation at this site mitigates the development of diabetic and prediabetic retinopathy. This is achieved by reducing VE-cadherin internalization, attenuating HBP activation, and decreasing Ang2 expression. Finally, O-GlcNAc–enriched retinal proteomic profiling indicates that the Y685F mutation restores imbalanced neurovascular and mitochondrial pathways.

The primary finding of this study is that phosphorylation of VE-cadherin at Y685 is essential for HBP activation and Ang2 regulation, driving vascular damage in diabetic and prediabetic retinopathy. Consistent with prior knockout and inhibitor-based studies, our results indicate that O-GlcNAc and Ang2 play critical roles in the retinas of both diabetic and NDPKB-deficient models (27, 30, 31). Tyrosine phosphorylation sites in VE-cadherin have been studied in the context of DR. Much of the existing literature has focused on VE-cadherin’s role in the angiogenic properties of retinal vessels during the late-stage DR, while less is known about the phosphorylation of these sites in the early stage (32, 33). Although HG-induced VE-cadherin phosphorylation at sites Y731 and Y658 has been described in in vitro and in vivo studies, contrasting findings have been reported. Limited evidence suggests that VE-cadherin phosphorylation at Y685 is induced in mouse models of DR (11, 34, 35). The lack of commercially available antibodies specific to mouse VE-cadherin at Y685 has hindered precise investigation of its role in transgenic mouse models. Our study aimed to identify targets linked to HBP activation, focusing on Y685, which is upregulated in both HG-treated and NDPKB-depleted ECs. The key findings from our study underscore the essential role of VE-cadherin Y685 phosphorylation, as demonstrated by viral overexpression and the Y685 mutant in ECs, as well as by in vivo experiments with VEC Y685F^ki/ki^ mice. The experimental results showed that suppression of its phosphorylation at Y685 reduces vascular damage, vessel hyperpermeability, and neuronal degeneration induced by HBP activation under hyperglycemia and NDPKB deficiency. Our findings support previous reports by Wessel et al., who found Y685 as a key regulator of endothelial permeability in pathological conditions (9). Ang2 is elevated in DR and destabilizes vessels by antagonizing the TEK receptor tyrosine kinase 2 (Tie2) pathway, promoting pericyte detachment and loss. Ang2 also disrupts endothelial junctional integrity and increases vascular permeability, in part through effects on VE-cadherin (4, 36). This study provides evidence for a reverse regulatory mechanism whereby VE-cadherin Y685 phosphorylation controls Ang2 upregulation under diabetic conditions. These findings suggest a possible vicious cycle between Ang2 and VEC Y685-mediated hyperpermeability. Notably, no compensatory increase in phosphorylation at Y731 and Y658 was observed following Y685 alteration, indicating that VE-cadherin exerts its effects on ECs undergoing HBP activation in a Y685-dependent manner. Disrupting the VE-cadherin Y685-mediated signaling pathway effectively blocks the pathological features of retinopathy, including pericyte and EC loss and retinal neuronal dysfunction.

Another important insight of this study is the link between Y685 phosphorylation and the regulation of protein O-GlcNAcylation and Ang2 expression. Studies have reported that Y685 phosphorylation under HG or HBP activation occurs after prolonged exposure, though this regulation typically occurs after extended exposure to HG (4). In contrast, our study provides clear evidence that VE-cadherin phosphorylation at Y685 begins as early as 30 minutes after HG stimulation, followed by increases in protein O-GlcNAcylation at 12 hours and upregulation of Ang2 expression at 24 hours in ECs. This suggests that phosphorylation of VE-cadherin at Y685 acts as an early mediator in the glucose-triggered elevation of O-GlcNAcylation, which in turn promotes Ang2 upregulation. Both HG and NDPKB deficiency increase VE-cadherin internalization. VE-cadherin Y685 overexpression and mutation experiments further support VE-cadherin Y685 phosphorylation as a key regulator of HBP activation-induced endothelial damage, likely via VE-cadherin internalization, highlighting the critical role of VE-cadherin internalization in glucose-associated endothelial damage. In this study, we focused specifically on the role of VEC Y685 in metabolic disorders. Further investigations will be required to clarify the underlying mechanisms related to HG and NDPKB deficiency–induced VEC pY685 activation and VE-cadherin internalization.

In our study, exposure to HG and depletion of NDPKB in ECs decreased VE-cadherin expression at the plasma membrane, which is consistent with previous reports showing reduced VE-cadherin on the cell surface under diabetic conditions (37). It is well established that phosphorylation of VE-cadherin at Y685 promotes its internalization, contributing to vascular hyperpermeability in pathological conditions (10). Ting et al. demonstrated that maintaining cellular VE-cadherin levels protects against HG-induced DR (38). Our study suggests that preventing Y685 phosphorylation early on, before VE-cadherin levels change, is sufficient to prevent the development of diabetic and prediabetic retinopathy. The underlying mechanism by which phosphorylation of VE-cadherin at Y685 activates the HBP in vascular ECs remains unclear. Hart et al. proposed that the inhibition of protein phosphorylation could be a result of increased protein O-GlcNAcylation under HG, as phosphorylation and O-GlcNAcylation compete for the same serine and/or threonine residues on proteins (39). In our study, however, we observed the opposite effect: phosphorylation at Y685 determines protein GlcNAcylation in ECs.

Our proteomic analysis revealed a diabetes-induced module of O-GlcNAc-modified proteins enriched for neurovascular and mitochondrial pathways. These signatures were markedly counter-regulated in DC VEC Y685F^ki/ki^ retinas, indicating that endothelial junctional signaling influences global O-GlcNAcylation patterns within the neurovascular unit. Among the most prominent changes was a neuronal response involving synaptic membrane components, which was normalized in the VEC Y685F^ki/ki^ mutant. In addition, we found that several immune response proteins displayed pronounced alterations in O-GlcNAcylation. These molecular signatures align with the growing consensus that DR is not solely a vascular pathology but a disorder of the neurovascular unit, wherein neuronal, immune, and vascular compartments exhibit reciprocal dysfunction (40). Furthermore, the diabetes-induced O-GlcNAc protein module we identified overlaps with ontology signatures reported in recent proteomic analyses of STZ-induced diabetic retinas, which documented extensive O-GlcNAc–dependent remodeling of metabolic and synaptic protein networks (41). The normalization of this neuronal pathological module in DC VEC Y685F^ki/ki^, together with restored ERG responses, further indicate that vascular dysfunction, specifically impaired endothelial barrier integrity and junctional signaling, modulates neuronal homeostasis through altered O-GlcNAcylation in adjacent neurons.

Our study further demonstrated that endothelial O-GlcNAcylation of proteins involved in insulin and VEGF receptor signaling as well as membrane regulation of surface turnover and trafficking is associated with the VEC Y685F mutation-promoted normalization of retinal pathology. These findings support previous reports indicating that VEGFR2 Y949-mediated regulation of VE-cadherin Y685 phosphorylation represents a potential therapeutic target for suppressing angiogenic pathology in late-stage DR (33). Although membrane proteins are rarely O-GlcNAcylated (42), it remains to be determined whether intracellular domains of such membrane-associated proteins are subject to regulation by O-GlcNAcylation. These data suggest that interfering with VE-cadherin Y685 phosphorylation restores vascular function by normalizing endothelial protein O-GlcNAc remodeling.

Notably, retinas carrying the VEC Y685F mutation maintained normal O-GlcNAcylation of mitochondrial proteins that support the bidirectional regulation of mitochondrial homeostasis under diabetic conditions. These findings align with reports in STZ-induced diabetic rat hearts, where both decreases and increases in O-GlcNAc modifications were observed, reflecting bidirectional O-GlcNAc remodeling (43). Together, these observations support a critical role for VEC Y685-mediated mitochondrial protein O-GlcNAcylation in preserving mitochondrial integrity during diabetes.

In addition to HBP activation, our data show that suppressing VE-cadherin Y685 phosphorylation normalizes elevated Ang2 levels, potentially improving retinal morphology and mitigating pericyte coverage. These results support previous studies on the role of Ang2 in the development of diabetic and prediabetic retinopathy (27, 44, 45). Ang2 levels are low under normal physiological conditions but increase in pathological conditions like DR. Our prior work demonstrated that both diabetic Ang2LacZ^+/–^ mice and NDPKB^–/–^ Ang2LacZ^+/–^ mice, with partial Ang2 loss, exhibit reduced pericyte loss compared with diabetic and NDPKB^–/–^ littermates, which phenocopies key features of the protection conferred by VE-cadherin Y685F, supporting that the Y685F benefit in the retina is ultimately mediated by attenuation of Ang2 (27, 30). The levels of Ang2 are controlled by multiple factors, including its receptor tyrosine kinase with Tie2, transcription factors, and O-GlcNAcylation (19, 24, 27, 46–48). Ang2 binds to Tie2 and inhibits its phosphorylation, thus interrupting Tie2-mediated signaling pathways. Our previous study also suggests that modulation of Tie2 expression by siRNA and Tie2 phosphorylation by soluble Tie2 decreases Ang2 levels in NDPKB-deficient ECs (49). As discussed above, whether Tie2 expression or its phosphorylation mediates Ang2 downregulation under inhibition of Y685 phosphorylation requires further investigations. Ang2 is markedly O-GlcNAcylated (24), and if Y685F mutation-induced Ang2 downregulation is mediated by altered Ang2 GlcNAcylation should be further explored. Our experiments using pharmacological modulation of protein O-GlcNAcylation highlight the crucial role of O-GlcNAc cycling in regulating Ang2 levels in ECs under HG and NDPKB-deficient conditions. Notably, the ability of O-GlcNAcylation to control Ang2 expression depending on VE-cadherin Y685 phosphorylation serves as a key regulatory mechanism. When Y685 phosphorylation was blocked, HG- or NDPKB-driven increase in O-GlcNAcylation no longer upregulates Ang2, despite persistently elevated global O-GlcNAc levels. Protein O-GlcNAcylation and VE-cadherin Y685 phosphorylation are essential for Ang2 upregulation, resulting in endothelial damage.

The vascular endothelial protein tyrosine phosphatase (VE-PTP) regulates endothelial stability by dephosphorylating Tie2 (50). Inhibiting VE-PTP activates Tie2, which stabilizes endothelial junctions via phosphorylation signaling (51). VE-PTP also dephosphorylates VE-cadherin at Y685, reinforcing junction stability in active endothelium (52). Interestingly, Frye et al. showed that the effects of VE-PTP inhibition depend on Tie2 activity and it stabilizes endothelial junctions in its presence but leads to destabilization if Tie2 is absent (53). It has been reported that VE-PTP regulates the actions of Ang2 in a context-dependent manner (54). Despite growing insight into Tie2 signaling in this context, the role of VE-cadherin, in particular, the relevance of its internalization to junction stabilization, remains poorly understood. Further studies are needed to determine whether and how VE-PTP regulates Y685 phosphorylation, protein O-GlcNAcylation, and Ang2 in metabolic disorders.

**Figure 9:**
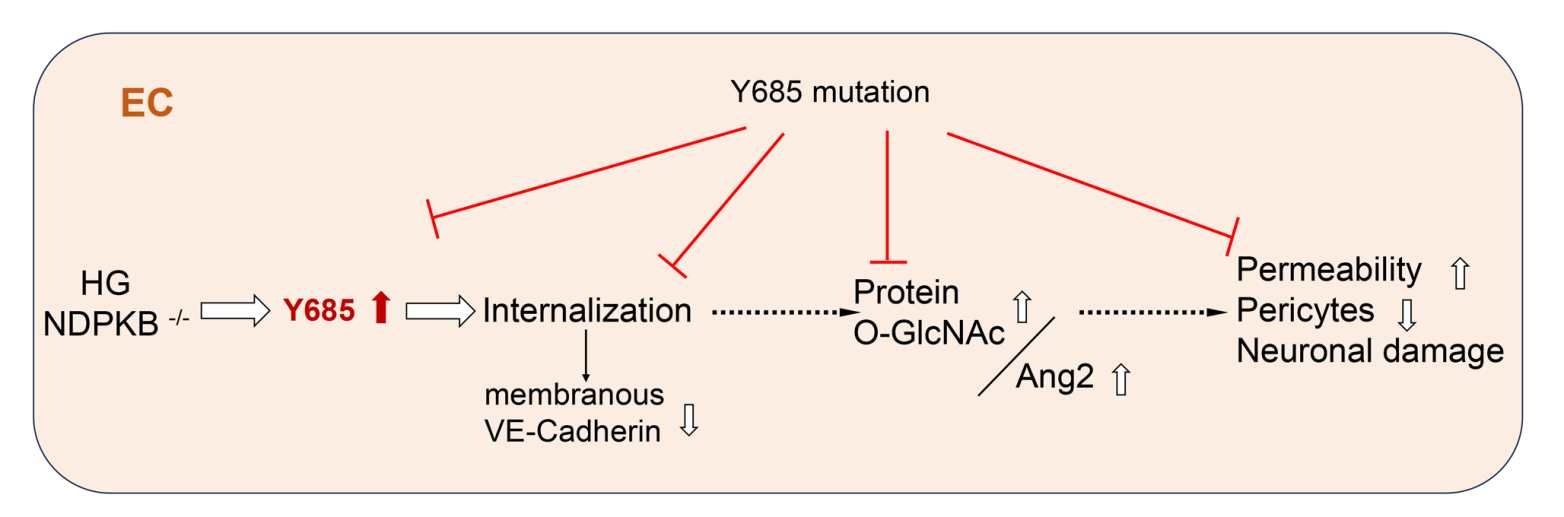
Suppressing VE-cadherin Y685 phosphorylation inhibits development of diabetic and prediabetic retinopathy. Both hyperglycemia and NDPKB deficiency activate VE-cadherin Y685 phosphorylation. This phosphorylation is necessary for the constitutive internalization of VE-cadherin, which compromises the integrity of ECs in the retinal vasculature. Under these pathological conditions, the HBP is activated, and Ang2 is upregulated, leading to vascular and neuronal damage in diabetic and prediabetic conditions. In contrast, suppressing VE-cadherin Y685 phosphorylation reverses the impairments caused by HBP activation, including vascular and neuronal damage.

In conclusion, this study has shown that phosphorylation of VE-cadherin at Y685 precedes the endothelial elevation of protein O-GlcNAc and Ang2, leading to diabetic and prediabetic retinopathy. Inhibition of this phosphorylation hinders endothelial damage and the development of HBP activation–induced retinopathy (Figure 9). Intervention approaches to inhibit the VE-cadherin phosphorylation at Y685 in ECs might be instrumental in identifying organ-specific therapeutic opportunities to prevent the development of diabetic and prediabetic retinopathy.

## Methods

Further information can be found in Supplemental Methods and Supplemental Table 1.

### Sex as a biological variable.

The in vivo part of the study was conducted in male mice and identified VE-cadherin Y685 phosphorylation as a key mediator of prediabetic and diabetic retinal vascular damage. Given that sex hormones can influence the magnitude and kinetics of endothelial responses, it is likely that this molecular pathway also operates in females. However, direct comparative studies will be required to determine whether sex differences affect the modulation or severity of Y685-mediated retinal pathology.

### Reagent.

All reagents are listed in Supplemental Table 1.

### Animals.

Ins2^Akita^ mice were purchased from The Jackson Laboratory, VEC Y685F^ki/ki^ mouse line was provided by Dietmar Vestweber (Max-Planck-Institute for Molecular Biomedicine, Münster, Germany), and NDPKB^–/–^ mouse line was generated as previously described (9, 55). Double-transgenic VEC Y685F^ki/ki^ and NDPKB^–/–^ mice were obtained by crossbreeding VEC Y685F^ki/ki^ and NDPKB^–/–^ mice at the Medical Faculty Mannheim, University of Heidelberg. To maintain a uniform genetic background, WT littermates produced from heterozygous VE-cadherin Y685F^ki/+^ crossbreeding were used as control animals. Diabetic mice were induced by administering a single intraperitoneal injection of 145 mg/kg STZ after a 4-hour fasting period when the mice weighed approximately 25 g. Mice were classified as diabetic if their blood glucose levels exceeded 250 mg/dL in tests conducted at 2 and 4 days postinjection. At the end of the experiment, the mice were killed at 3 and 6 months after diabetes induction. HbA1c levels, blood glucose levels, and body weight were recorded, and the eyes were collected.

### Retinal whole-mount immunofluorescence.

Eyes were fixed in 4% paraformaldehyde (PFA) for 2 hours on ice, followed by the isolation of the retinas. After rinsing with phosphate-buffered saline (PBS), the retinas were treated with a mixture of 1% bovine serum albumin (BSA) and 0.5% Triton X-100 for permeabilization and blocking at room temperature for 1 hour. Subsequently, the retinas were incubated with goat anti–VE-cadherin antibody overnight at 4°C (dilution 1:200). To visualize retinal vessels, lectin conjugated with fluorescein isothiocyanate (Lectin-TRITC) (dilution 1:50) was used for costaining. Following this, retinas were incubated with the secondary antibody rabbit anti-goat Alexa Fluor 488 (dilution 1:200) at room temperature for 1 hour. Finally, the retinas were washed and flat-mounted with 4-leaf incisions to obtain optimal whole-mount preparations. Images of the retinal vasculature were captured using Leica DMRE microscopy (Leica).

### Retinal digestion and quantitative retinal morphometry.

To determine the numbers of retinal pericytes and ACs, retinal digestion and quantification method as detailed in a prior study were followed (56). In brief, eyes were fixed in 4% formalin at room temperature for 24 hours. Subsequently, isolated retinas were incubated in water at 37°C for 1 hour, followed by a 3-hour incubation in 3% trypsin dissolved in Tris-HCl buffer (pH 7.0) at 37°C. The pure retinal vasculature was obtained by washing the retinas with distilled water, drying them on microscope slides, and then staining with periodic acid–Schiff and hematoxylin. Pericytes were quantified in 10 randomly selected capillary fields at 40× original magnification using a microscope system (AnalysisPro; Olympus Opticals). The numbers of pericytes were standardized by capillary areas. ACs were counted in 10 randomly selected fields using an established method, as previously described (30).

### Dextran-FITC permeability assay.

Mice were anesthetized with isoflurane and intravenously injected via the tail vein with 70 kDa dextran-FITC (25 mg/kg). After a 30-minute circulation period, animals were sacrificed and perfused with PBS. Blood samples were collected immediately before perfusion. Eyes were enucleated following perfusion and processed for further analyses. For quantitative assessment of retinal FITC content, the retinas from both eyes of each mouse were carefully isolated, weighed, and homogenized in 250 μL of H_2_O. Each sample was measured in duplicate. FITC fluorescence was measured in black, 96-well plates using a fluorescence microplate reader (excitation: 485 nm, emission: 520 nm). A leakage index was calculated using the following formula: retinal FITC load (μg/mg tissue)/plasma FITC (μg/mL) × circulation time (h) (57, 58). To visualize the location of leakage sites, whole-mount retinas were stained with Lectin-TRITC (1:100) to delineate the vascular network and then imaged by Olympus microscopy.

### RNA interference.

HUVECs were seeded on 1% gelatin-coated, 6-well plates in endothelial cell basal medium (ECBM). Upon reaching 70%–80% confluence, the cells were transfected with 250 pmol of an NDPKB siRNA (5′-AGGUAGUGUAAUCGCCUUG-3′) (Eurofins, MWG) or scrambled siRNA 5′-AACUGGUUGACUACAAGUCUU-3′ (Eurofins MWG), using Lipofectamine RNAiMAX in a serum-free OptiPRO medium for 4 hours. After transfection, cells were recultured in the complete ECBM. Confirmation of NDPKB knockdown was achieved through immunoblotting analysis.

### Membrane protein extraction.

After transfection and HG stimulation as described before, HUVECs were harvested with trypsin-EDTA and centrifuged at 500*g* for 5 minutes. The cell pellets were then washed and suspended in ice-cold PBS. Membrane proteins were extracted using the Subcellular Protein Fractionation Kit. After adding the membrane extraction buffer and incubating at 4°C for 10 minutes, the membrane extract was obtained by centrifugation at 3,000*g* for 5 minutes. The samples were heated for protein denaturation at 95°C and subjected to immunoblotting.

### Adenovirus-mediated VE-cadherin Y685F mutant.

HUVECs were initially seeded in 1% gelatin-coated, 6 cm dishes and cultured in ECBM supplemented with 10% FCS at 37°C overnight until reaching 70%–80% confluence. The cells were then infected with recombinant adenovirus (Ad) encoding GFP, such as VE-cadherin Y685 wt (Ad-human VEC-WT-GFP), or VE-cadherin Y685F mutant (Ad-human VEC-Y685F mutant-GFP), in which Y685 was replaced, and the expression was mutated in ECBM containing 10% FCS for 24 hours. Transducing additional mutant VE-cadherin can suppress downstream signaling due to competitive incorporation and dominant-negative effects, despite the presence of endogenous VE-cadherin. In all comparisons, groups were equivalently transduced with VE-cadherin Ad to match total exogenous VE-cadherin. We compared Y685F-infected ECs directly with WT-infected ECs rather than empty-vector controls. GFP expression was monitored to confirm successful infection. After infection, cells were washed by PBS for further procedures. For transfection, NDPKB siRNA and control siRNA were utilized to knock down NDPKB or serve as a control. The following day, HUVECs were seeded in 1% gelatin-coated, 6-well plates and subjected to starvation. For HG stimulation, cells were seeded in 1% gelatin-coated 6-well plates, starved in ECBM with 0.5% FCS overnight, and then treated with HG for either 30 minutes or 24 hours. After treatment, cells were processed for immunoblotting, HUVEC-pericyte coculture, or VE-cadherin internalization assay.

### VE-cadherin internalization assay.

HUVECs were cultured on coverslips coated with cross-linked gelatin and glutaraldehyde. Cells were starved overnight with 0.5% FCS, followed by incubation with mouse anti–extracellular domain of human mouse anti–VE-cadherin (dilution 1:10) at 4°C for 1 hour in MCDB 131 medium containing 1% BSA. After washing with ice-cold MCDB 131 medium to remove unbound antibodies, cells were treated with HG for 30 minutes or 50 ng/mL VEGF for 15 minutes at 37°C. To assess internalized VE-cadherin, cells were washed with ice-cold washing buffer (Hank’s buffer, pH 2.7, containing 25 mM glycine and 1% BSA), fixed with 4% PFA for 15 minutes on ice, and blocked and permeabilized overnight at 4°C with 1% BSA containing 0.2% Triton X-100 in PBS. Following PBS washing, cells were incubated with corresponding secondary antibodies: goat anti-mouse Alexa Fluor 488 (dilution 1:200) or goat anti-mouse Alexa Fluor 555 (dilution 1:200) at room temperature for 1 hour to detect the internalized VE-cadherin. Nuclei were stained with DAPI for 10 minutes. After covering coverslips on microscope slides, images were captured by using the confocal microscope, and quantification was performed with ImageJ (NIH) by measuring the fluorescence intensity inside cells.

### Pericyte culture.

Human brain vascular pericytes were purchased from ScienCell Research Laboratories and later cultured on 1% gelatin-coated dishes in DMEM/F12 medium supplemented with 5% FCS, 1% penicillin-streptomycin, insulin (5 μg/mL), and basic fibroblast growth factor (2 ng/mL).

### Transwell HUVEC-pericyte contacting coculture.

Transwell inserts (24-well, 0.4 μm pore size, Corning) were used to seed HUVECs on the abluminal side of the membrane. Inserts were placed across a sterile, 12-well plate, and HUVEC suspension was carefully dispensed onto the underside of the membrane. After attachment, inserts were turned upright into companion wells containing medium and cultured overnight to obtain a continuous HUVEC monolayer. The next day, pericytes were labeled with CellTracker Red dye (2 μM) and seeded onto the luminal side of the membrane at a pericyte-to-HUVEC ratio of 1:1 in a mixed medium consisting of 50% HUVEC medium and 50% pericyte medium (59, 60). To quantify nonattached pericytes, the supernatants from the insert chamber were gently collected at specific time points, and CellTracker-positive nonattached cells in supernatants were counted. For attached pericyte quantification, at 48 hours of coculture, cells were fixed with 4% PFA and stained with DAPI. The number of CellTracker-positive pericytes on the membrane was quantified in 10 randomly selected microscope fields using ImageJ.

### ERG.

To assess neuronal function in the retina, multifocal ERG was conducted on mice. Mice were adapted to darkness ≥4 hours and subsequently performed the test under dim red light. The animals were anesthetized via intraperitoneal injection with a mixture of ketamine (75 mg/kg) and xylazine (3.75 mg/kg) and maintained on a heating pad at 37°C. Pupillary dilation was achieved by applying a topical drop of 0.5% tropicamide before the procedure. Mice were positioned in front of a scanning laser ophthalmoscope device (RETImap, Roland Consult), with a Dawson-Trick-Litzkow electrode placed on the cornea. The eyes of the mice were fitted with a +100-diopter contact lens (Roland Consult). An array of 6 equally sized hexagons was chosen for stimulation using 150 cd/m^2^ and 1 cd/m^2^ for the m-sequence. An average of 12 cycles per hexagon was utilized for final recording, processed with a built-in 50 Hz band-pass filter to reduce background noise. For each animal, the average amplitude of the 6 hexagons surrounding the optic nerve head was used for the final analysis.

### LC-MS/MS proteomic analysis.

Retinal lysates from NC WT, DC WT, NC VEC Y685F^ki/ki^, and DC VEC Y685F^ki/ki^ mice were subjected to O-GlcNAc–specific immunoprecipitation via succinylated wheat germ agglutinin (sWGA) followed by MS. O-GlcNAc–modified proteins were enriched from 2 retinas of each mouse homogenized immediately after dissection in 240 μL ice-cold RIPA lysis buffer. Protein concentration was determined by BCA assay. For each sample containing 0.5 mg total retinal proteins, 30 μL sWGA agarose beads were added. The mixtures were incubated with gentle rotation for 24 hours at 4°C. Beads were then washed to remove unbound material, and the bead-bound proteins were collected for subsequent LC-MS/MS analysis. sWGA-enriched proteins were eluted in 100 μL urea-HEPES lysis buffer, heated at 56°C for 20 minutes, cooled, and centrifuged at 25,000*g* for 15 minutes at 4°C. Proteins were reduced with 10 mM DTT (30 minutes, 37°C), alkylated with 55 mM iodoacetamide (45 minutes, dark), and digested with sequencing-grade trypsin. Peptides were desalted and analyzed by nanoLC-MS/MS in data-independent acquisition (DIA) mode on an Astral mass spectrometer coupled to a Vanquish Neo nanoLC system (Thermo Fisher Scientific) according to the manufacturer’s recommendations. The sample data were further processed by bioinformatic analysis with the R software (Version 4.4.3). Proteins with fewer than 3 quantified data points were omitted from subsequent analyses. PCA was performed on log2-transformed, normalized label-free intensities from the O-GlcNAc immunoenriched retinal proteomes of 4 groups. Volcano plots were generated for the indicated pairwise contrasts: DC WT to NC WT, DC VEC Y685F^ki/ki^ to NC VEC Y685F^ki/ki^, and DC VEC Y685F^ki/ki^ to DC WT. Fold-changes were calculated as the ratio of group means (log2 scale), and proteins were considered differentially enriched at nominal *P* < 0.05 and absolute fold-change |log2FC| ≥ 0.263. Proteins above threshold were classified as increased or decreased. Pathway annotations were created with Metascape, a 2-contrast intersection strategy. Proteins increased in DC WT relative to NC WT, and those increased in DC WT relative to DC VEC Y685F^ki/ki^, were subjected to GO overrepresentation analysis (BP, CC, MF). GO terms significant in both were intersected to define WT-diabetic–elevated pathways. The same was done for decreased proteins to identify reduced pathways in diabetic retinas. Top terms are shown as bar plots. Proteins belonging to consensus pathways were *Z*-scored by row and visualized as hierarchically clustered heatmaps.

### Statistics.

The data are presented as means ± SD. Differences between the 2 groups were analyzed using an unpaired 2-tailed *t* test or the Mann-Whitney *U* test. For comparisons involving more than 2 groups, 1-way ANOVA with Tukey’s post hoc test for multiple comparisons was performed. Statistical analyses were performed with Prism 10 software (GraphPad Software). Values with *P* < 0.05 were considered statistically significant.

### Study approval.

All animal experiments were approved by the local ethics committee (G165/20, Regierungspräsidium Karlsruhe). The care and experimental use of all animals in the study were conducted following institutional guidelines and the Association for Research in Vision and Ophthalmology statement.

### Data availability.

The mass spectrometry proteomics data have been deposited to the ProteomeXchange Consortium via the PRIDE partner repository under accession number PXD072047. Supplemental proteomics values are provided in Supplemental Table 2. All original values for statistical analysis are shown in the Supporting Data Values file.

## Author contributions

YF, TW, HPH, and GD were involved in designing and guiding the studies. HH and RE performed retina digestion, albumin, and VE-cadherin immunoblotting in NDPKB^–/–^ mice. YW and HH performed and analyzed the in vitro experiments in ECs and pericytes. FS and YW investigated the morphology and phenotype alternations in VEC Y685F^ki/ki^ animal models. MQ and YW performed the vascular permeability assay with dextran-FITC. NK, YR, and GD conducted proteomic analysis in diabetic WT and VEC Y685F^ki/ki^ animal models. The manuscript was written by YW, HH, FS, MQ, NK, and YF. All authors have reviewed, edited, and approved the final version of the manuscript.

The order of co–first authors was determined by the duration of their contributions to the project, with YW mainly performing the in vitro experiments and finalizing the study, HH conducting the early exploratory work that defined the project subject, FS carrying out the animal studies, and all three contributing to the writing.

## Conflict of interest

The authors have declared that no conflict of interest exists.

## Funding support

- Deutsche Forschungsgemeinschaft (DFG; German Research Foundation, project 511337830 for YF).

## Supplementary Material

Supplemental data

Unedited blot and gel images

Supplemental table 2

Supporting data values

## Version 1 05/15/2026

Electronic publication
