# Supplementary material for: Disrupting VE-cadherin Y685 phosphorylation inhibits development of experimental diabetic and prediabetic retinopathy
Source: J Clin Invest. 2026 May 15;136(10):e195048. doi: 10.1172/JCI195048 (PMC13178665; doi:10.1172/JCI195048)
Supplement: Supplemental data [file jci-136-195048-s021.pdf]

Supplementary Methods

Isolation and culture of ECs

The use of HUVECs was approved by the local medical ethics committee (Medical Faculty Mannheim, Heidelberg University, Germany). HUVECs were isolated from the umbilical cords of healthy newborn babies with the mothers' informed consent. HUVECs were obtained by digestion with 1mg/mL dispase (Sigma-Aldrich, 4942078001, Taufkirchen, Germany), and suspended in 10% FCS (Promocell, C-37350, Heidelberg, Germany) to stop the enzyme activity. Cell suspensions were seeded in endothelial cell basal medium (ECBM, Promocell, C-22210, Heidelberg, Germany) with 2% FCS and grown on 1% gelatin-coated T25 culture flasks in a humidified incubator at 37°C and 5% CO_2. The culture medium was changed after 2 h, and cells were split after reaching confluence and reseeded in 1% gelatin-coated T75 culture flasks in ECBM with 10% FCS. Cells up to passage 3 were used for experiments and seeded on the 1% gelatin-coated 6-well plates. For stimulation, after an overnight starvation period with 0.5% FCS, the cells were stimulated with freshly made HG (30 mM) for either 30 min or 24 h, with or without TMG (10 µM) or OSMI-4 (5 µM).

MBMECs were isolated from 10-15-week-old female mice, using three to four brains for each isolation. MBMECs were isolated as described previously (1). Isolated MBMECs were suspended in ECBM supplemented with 20% FCS, seeded on a 1mg/ml collagen-coated plate, and maintained at 37°C in a humidified incubator. Endothelial cell selection was carried out on the following day by incubating the cells for 3 days in ECBM with 4 µg/mL puromycin and 20% FCS.

Immunoblotting

HUVEC and retinal proteins were lysed in RIPA buffer (50 mM Tris-HCl, pH7.4, 150 mM NaCl, 1 mM dithiothreitol, 1% Triton X-100, 1% sodium deoxycholate) supplemented with fresh protease inhibitor cocktail and phosphatase inhibitor cocktail. After incubation at 4°C for 15 min, proteins

were obtained by centrifugation. The samples were then heated for protein denaturation at 95°C after adding 4 × Laemmli buffer. After separation by SDS-PAGE, proteins were transferred onto nitrocellulose membranes. Following blocking with Roti-block, the blots were incubated overnight at 4°C with primary antibodies. Subsequently, after washing with Tris-buffered saline with Tween 20, the blots were incubated with the corresponding secondary antibodies for 1 h at room temperature. Protein visualization was achieved by using chemiluminescent peroxidase substrates (Lumi light, 2015200, Roche, Mannheim, Germany; or Femto, 34095, Thermo Scientific, Rockford, USA). Quantification was performed using Image J (NIH, USA). Antibodies utilized can be found in Supplementary Table 1. For retinal albumin immunoblotting to assess vascular permeability, mice were perfused via the left ventricle with PBS to remove intravascular blood and circulating albumin. Eyes were then collected immediately, snap-frozen in liquid nitrogen, and stored at −80°C until lysed.

Immunofluorescence

HUVECs were seeded on 1% gelatin-coated 24-well plates with round coverslips inside. After all treatments, cells were fixed in 4% PFA for 10 min, followed by washing with PBS. Subsequently, cells were blocked with 1% BSA and permeabilized with 0.1% Triton X-100. After PBS washing, cells underwent staining for membranous VE-cadherin by incubating with goat-anti-VE-cadherin antibody (dilution 1:200) diluted in 1% BSA containing 0.1% Triton X-100 overnight at 4°C. Following three washes with 0.1% Triton X-100 in PBS, cells were incubated with corresponding secondary antibodies rabbit anti-goat IgG (H+L) conjugated with Alexa Fluor 488 (dilution 1:200) at room temperature for 1 h. Nuclei were stained with DAPI. Images were acquired using Leica SP8 (Leica, Germany). Quantification and analysis of membranous VE-cadherin was performed by measuring mean fluorescent intensity with Image J (NIH, USA) as described previously.

Immunofluorescence staining of paraffin section

Paraffin-embedded tissue sections were baked at 60 °C for 70 min and deparaffinized in Roti Histol. Sections were rehydrated through a graded ethanol series. Heat-induced antigen retrieval was performed in a microwave at 500 W for 10 min, followed by 270 W for 10 min. After cooling, tissue boundaries were circumscribed, permeabilized, and blocked in blocking buffer (1% BSA, 0.25% Triton X-100 in PBS) for 15 min at room temperature. Rabbit-anti-GFAP diluted in PBS (1:500) was applied and incubated overnight at 4 °C. The next day, the slides were incubated with the secondary antibody goat anti-rabbit Cy3 (dilution 1:200) at room temperature for 1 h. Following washes, sections were mounted in Roti-Mount FluorCare and imaged using a Leica SP8 confocal microscope.

Immunoprecipitation

HUVECs were seeded in 10 cm dishes and transfected with VE-cadherin Y685 wt or VE-cadherin Y685F mutant adenovirus. Cells from each 10 cm dish were lysed on ice in 500 µL RIPA-WB buffer supplemented with protease inhibitors and PhosSTOP. Lysates were incubated on ice for 30 min, then clarified by centrifugation, followed by protein concentration measurement. For each sample, 400 µL of lysate at 1 µg/µL was used. Eight µg of VE-cadherin antibody D87F2 was added to each lysate and incubated for 2 h at 4 °C with rotation. Protein A/G beads were prepared by aliquoting 50 µL per sample, washing with RIPA buffer, and resuspending in RIPA buffer to a final volume of 100 µL per sample. The bead suspension was added to the antibody-lysate mixtures and incubated overnight at 4 °C. The next day, immune complexes were washed. Beads were then mixed with 60 µL RIPA-WB per sample, followed by the addition of 4× SDS loading buffer to a final volume of 80 µL. Samples were boiled at 95 °C for 5 min, and eluates were analyzed by SDS-PAGE and immunoblotting.

HUVEC-pericyte direct co-culture

For the co-culture experiments with Y685F mutation in HUVECs, ECs were transfected with or without siNDPKB and infected by Y685F mutation adenovirus. The next day, HUVECs were counted and seeded into 1% gelatin-coated 24-well plates. After 24 h, pericytes were stained with Cell-Tracker^{TM} Red dye (2 μM) in a serum-free medium at 37 °C for 30 min and seeded onto the HUVEC monolayer, at a ratio of 1:3 and co-cultured for an additional 24 h in mixed medium containing 50% HUVEC medium and 50% pericyte medium, using mixed medium with or without HG treatment (2). For quantifying non-attached or detached pericytes, cells were collected from the supernatants after 2, 4, 6, 8, 10, and 24 h of co-culture and counted.

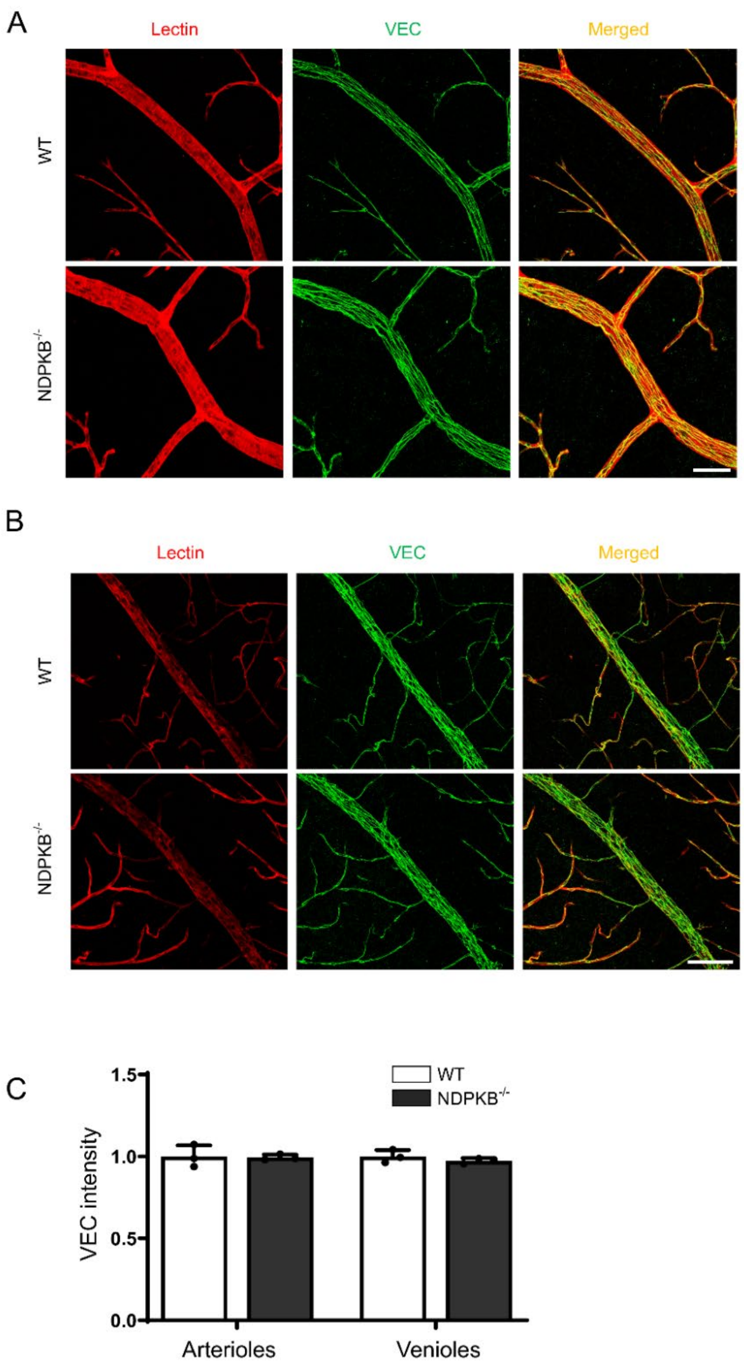

Supplementary Fig. 1: VE-cadherin is not altered in the retinal arterioles and venules in NDPKB$^{-/-}$ retinas

Immunofluorescence staining (A and B) and quantification (C) of VE-cadherin in the retinal arterioles (A) and venules (B) visualized with vascular marker lectin (red) and VE-cadherin (green). n = 3. WT: wild-type; NDPKB$^{-/-}$: NDPKB homozygous. Statistical analysis was performed by unpaired two-tailed t-test. Scale bar: 50 μm.

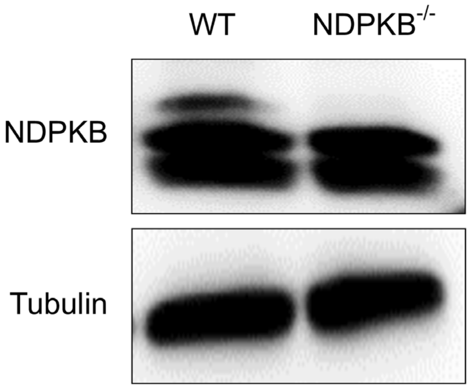

Supplementary Fig. 2: NDPKB is absent in the NDPKB$^{-/-}$ retinas

Immunoblotting of NDPKB in NDPKB$^{-/-}$ retinas. WT: wild-type; NDPKB$^{-/-}$: NDPKB homozygous.

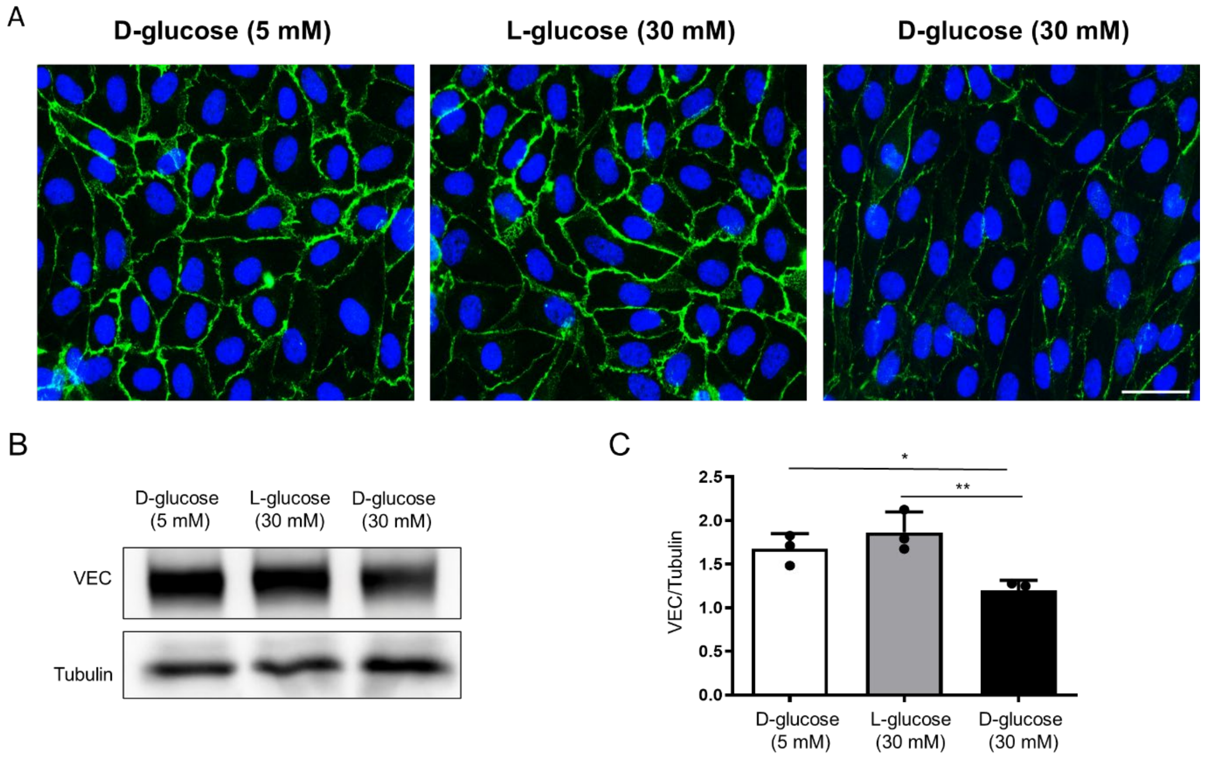

Supplementary Fig. 3: L-glucose does not affect expression of VE-cadherin in ECs

(A) Immunofluorescence of VE-cadherin expression in L-glucose- and D-glucose-treated HUVECs. Immunoblotting (B) and statistical analysis (C) of VE-cadherin expression in L-glucose- and D-glucose-treated HUVECs. n = 3. Overall P = 0.0104. L-G: L-glucose; D-G: D-glucose. VEC: VE-cadherin. * $P < 0.05$, ** $P < 0.01$. Statistical significance was determined by one-way ANOVA with Tukey's post hoc test for multiple comparisons. Scale bar: 50 μm.

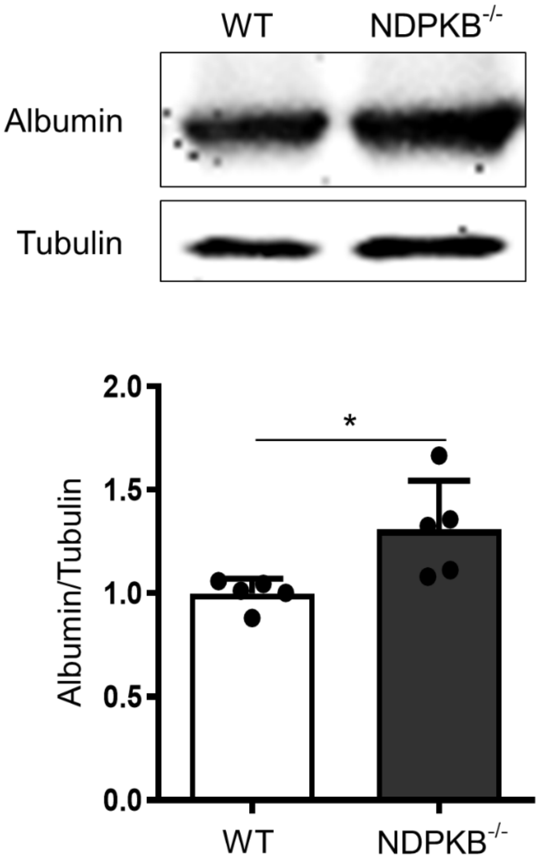

Supplementary Fig. 4: Vascular hyperpermeability was observed in 5-month-old NDPKB$^{-/-}$ retinas using albumin

Immunoblotting and quantification of retinal albumin levels to evaluate retinal vascular permeability. n = 5. WT: wild-type; NDPKB$^{-/-}$: NDPKB homozygous. *P < 0.05. Statistical analysis was performed by unpaired two-tailed t-test.

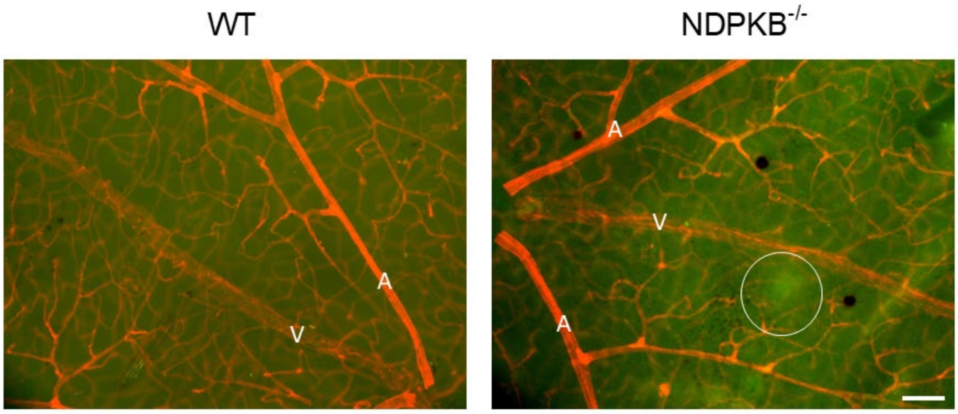

Supplementary Fig. 5: Vascular hyperpermeability in NDPKB-deficient retinas

Representative whole-mount retinal images from WT and NDPKB$^{-/-}$ mice following tail-vein injection of dextran-FITC and subsequent perfusion. Retinal vessels were counterstained with lectin-TRITC to visualize the retinal vascular network. The white circle marks a focal area of pronounced extravascular dextran-FITC accumulation, indicating vascular leakage. NDPKB$^{-/-}$: NDPKB-deficient mice; A: arterioles; V: venules. Scale bar: 50 µm.

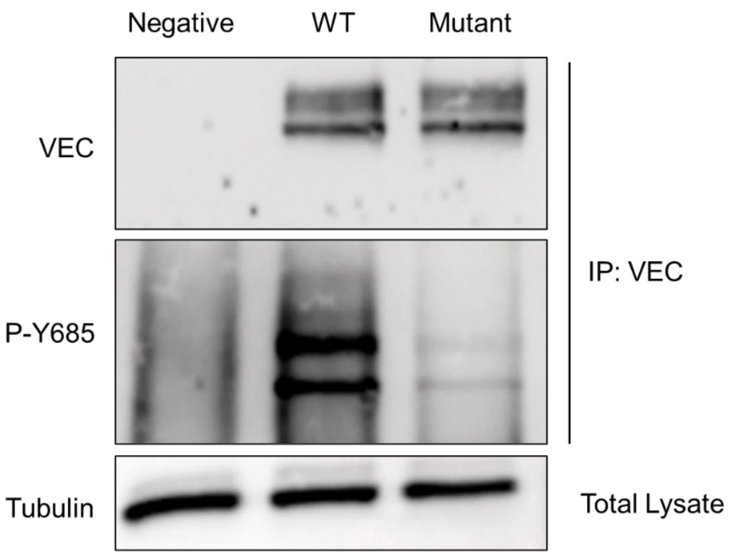

Supplementary Fig. 6: Test of an appropriate VE-cadherin pY685 antibody ab119785 for its semi-quantitation in ECs

HUVECs were transduced with adenovirus encoding WT VE-cadherin or the Y685F mutant VE-cadherin. VE-cadherin was immunoprecipitated and blotted with anti-pY685 and VE-cadherin. WT: VE-cadherin Y685; Mut: VE-cadherin Y685F mutation.

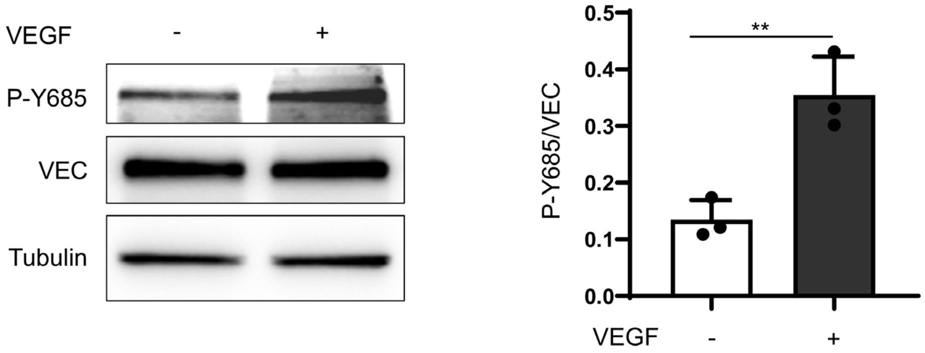

Supplementary Fig. 7: VE-cadherin Y685 is increased in HUVECs after treatment with VEGF

Immunoblots and quantification of VE-cadherin Y685 in HUVECs treated with VEGF. n = 3. VEC: VE-cadherin; VEGF: vascular endothelial growth factor. **P < 0.01. Statistical analysis was performed by unpaired two-tailed t-test.

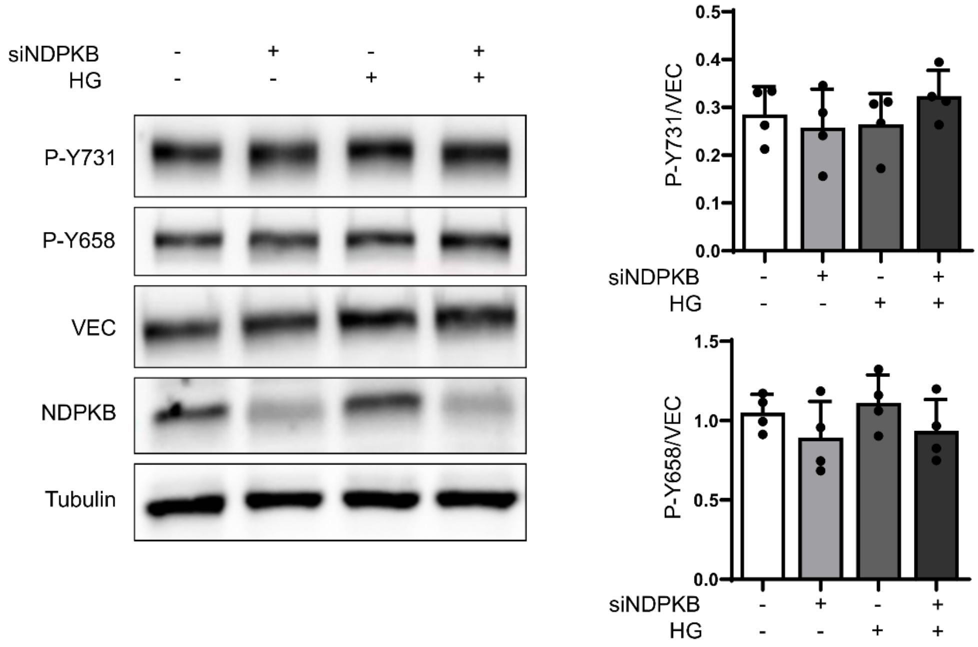

Supplementary Fig. 8: Phosphorylation of VE-cadherin at Y658 and Y731 remained unaltered under HG and NDPKB deficiency

Immunoblots and quantification of phosphorylation of VE-cadherin at Y658 (overall P = 0.3504) and Y731 (overall P = 0.5047) under both HG and NDPKB-deficient conditions. n = 4. HG: high glucose; VEC: VE-cadherin; siNDPKB: NDPKB siRNA. Statistical analysis was performed by one-way ANOVA with Tukey’s post hoc test for multiple comparisons.

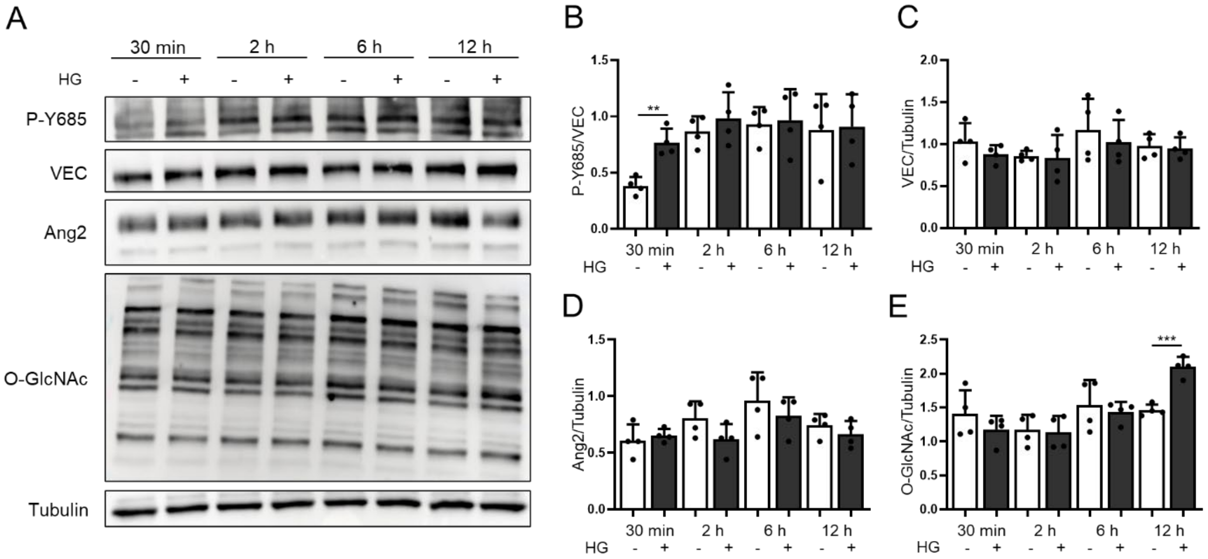

Supplementary Fig. 9: HG stimulation induces a rapid increase in VE-cadherin Y685 phosphorylation at 30 min and a significant elevation in global O-GlcNAcylation at 12 h in ECs

(A) Representative immunoblotting analysis of P-Y685, VE-cadherin, Ang2 and O-GlcNAc in HG-treated HUVECs for the indicated times (30 min, 2 h, 6 h, 12 h). Quantification of P-Y685 (B), VE-cadherin (C), Ang2 (D) and O-GlcNAc (E) are shown. n = 4. VEC: VE-cadherin; HG: high glucose. **$P < 0.01$, ***$P < 0.001$. Statistical analysis was performed by unpaired two-tailed t-test.

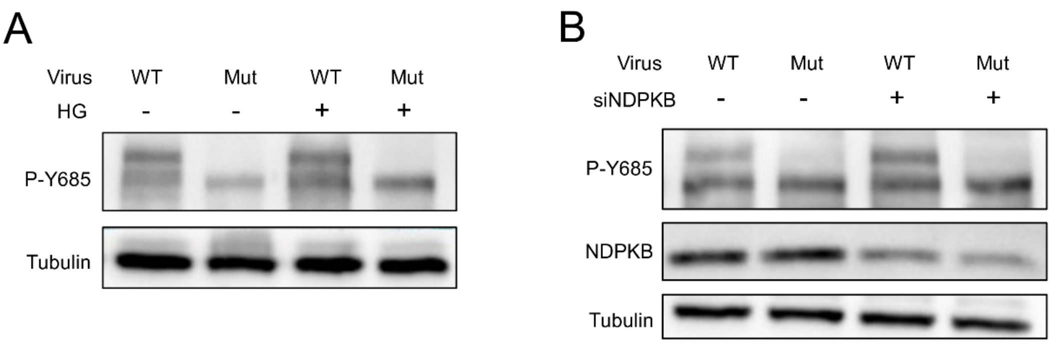

Supplementary Fig. 10: Adenoviral Y685F mutant VE-cadherin prompts suppression of Y685 phosphorylation in HUVECs

(A) Representative immunoblotting analysis of VE-cadherin in HG-treated HUVECs infected with adenovirus VE-cadherin Y685 (WT), or VE-cadherin Y685F mutant (Mut). (B) Representative immunoblotting analysis of Y685 in NDPKB-depleted HUVECs infected with WT, or Mut adenovirus. WT: VE-cadherin Y685; Mut: VE-cadherin Y685F mutation; HG: high glucose; siNDPKB: NDPKB siRNA.

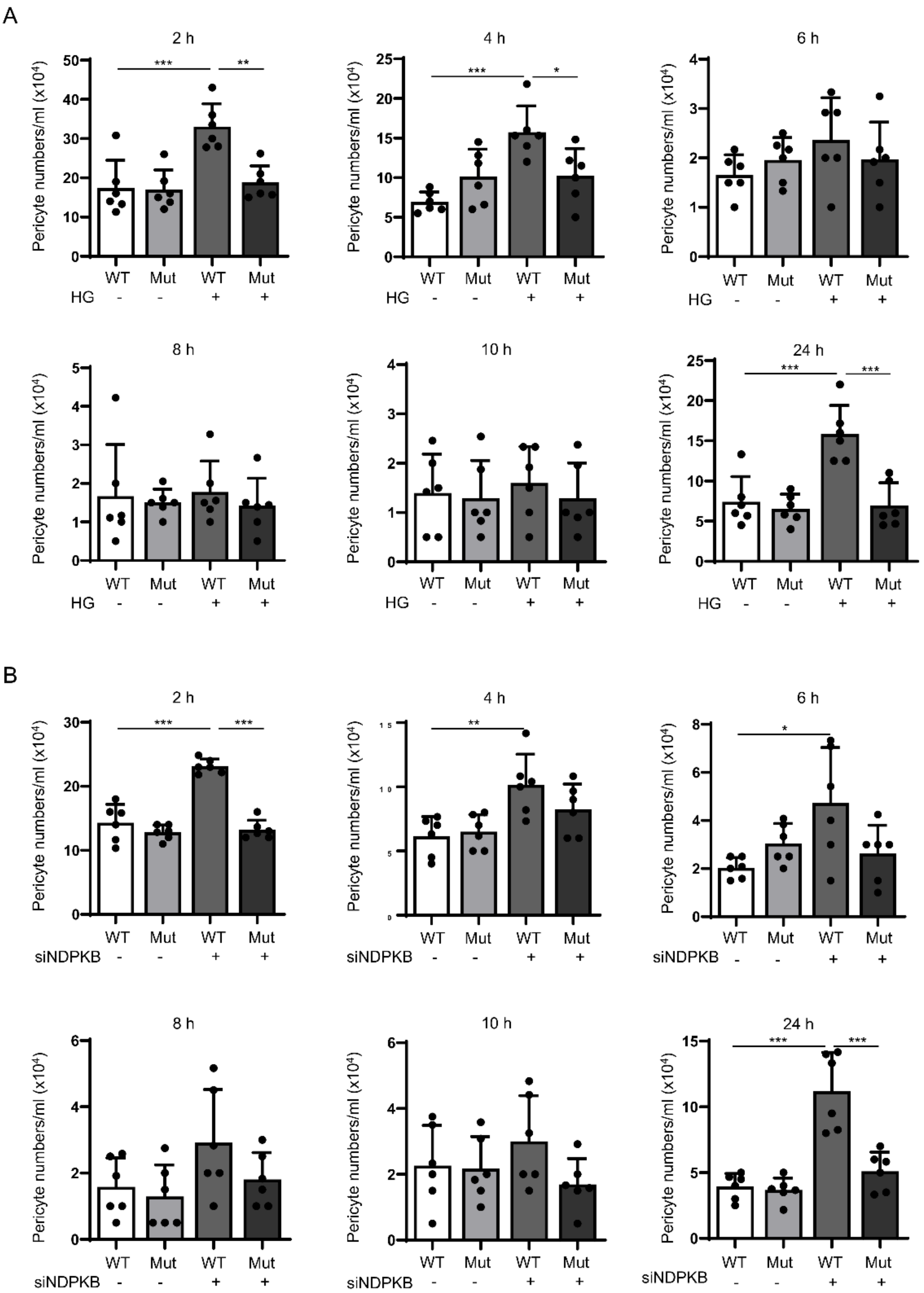
A
2 h
4 h
6 h
8 h
10 h
24 h
Pericyte numbers/ml (x10⁴)
WT
Mut
WT
Mut
HG - - + +
***
**
*
B
2 h
4 h
6 h
8 h
10 h
24 h
Pericyte numbers/ml (x10⁴)
WT
Mut
WT
Mut
siNDPKB - - + +

Supplementary Fig. 11: Pericyte attachment in direct pericyte-EC co-culture is protected by Y685F mutation in HG and NDPKB deficiency

Analysis of the non-attached and detached pericytes labeled with Tracker™ in co-culture with HG treatment (2 h overall P < 0.001; 4 h overall P < 0.001; 6 h overall P = 0.3332; 8 h overall P = 0.894; 10 h overall P = 0.8803; 24 h overall P < 0.001) (A) and NDPKB-depleted HUVECs (B) (2 h overall P < 0.001; 4 h overall P = 0.0045; 6 h overall P = 0.0184; 8 h overall P = 0.0923; 10 h overall P = 0.273; 24 h overall P < 0.001) infected with adenovirus WT or Y685F Mut for 2, 4, 6,8, 10 and 24 h co-culture. n = 6. WT: VE-cadherin Y685; Mut: VE-cadherin Y685F mutation; HG: high glucose; siNDPKB: NDPKB siRNA. *P < 0.05, **P < 0.01, ***P < 0.001. Significance was determined by one-way ANOVA with Tukey’s post hoc test for multiple comparisons.

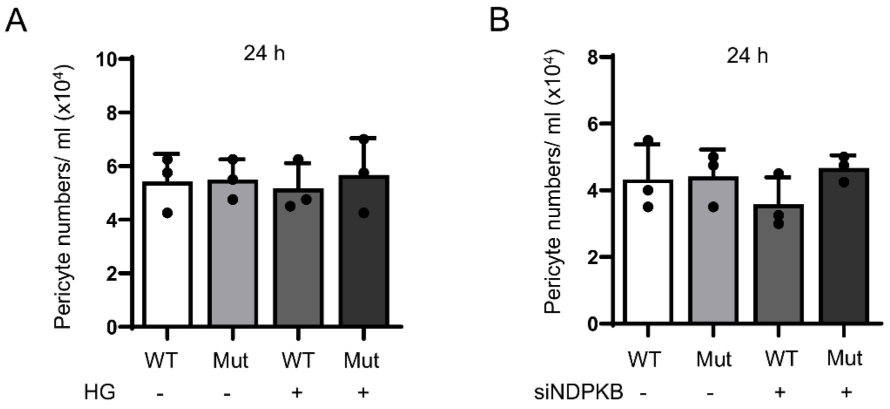

Supplementary Fig. 12: Pericyte detachment is comparable in Transwell contacting co-culture at 24 h

Analysis of the detached pericytes labeled with Tracker™ in supernatants with HUVECs infected with adenovirus WT or Mut for 24 h co-culture under HG conditions (overall P = 0.9474) (A) and NDPKB deficiency (overall P = 0.4273) (B). n = 3. WT: VE-cadherin Y685; Mut: VE-cadherin Y685F mutation; HG: high glucose; siNDPKB: NDPKB siRNA. Statistical analysis was performed by one-way ANOVA with Tukey’s post hoc test for multiple comparisons.

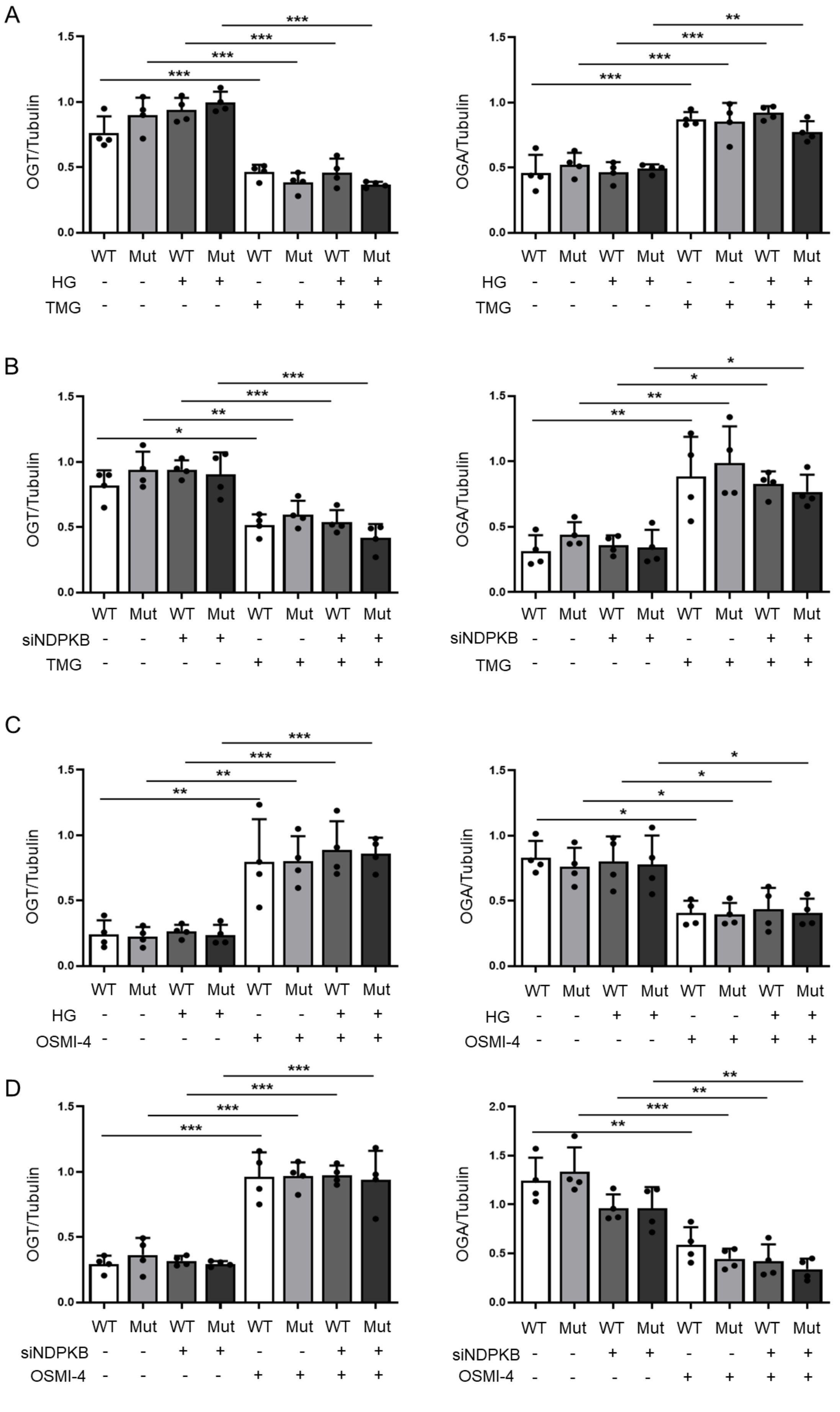
A
B
C
D
OGT/Tubulin
OGA/Tubulin
0.0
0.5
1.0
1.5
2.0
WT Mut WT Mut WT Mut WT Mut
HG - - + + - - + +
TMG - - - - + + + +
siNDPKB - - + + - - + +
OSMI-4 - - - - + + + +
*
**
***

Supplementary Fig. 13: TMG and OSMI-4 bidirectionally modulate OGT and OGA in ECs

Representative quantification of OGT and OGA expression in HG-treated (A) and NDPKB-depleted HUVECs (B) infected with adenovirus WT or Mut, combined with TMG treatment. Representative quantification of OGT and OGA expression in HG-treated HUVECs (C) and NDPKB-depleted HUVECs (D) infected with adenovirus WT or Mut, combined with OSMI-4 treatment. n = 4. WT: VE-cadherin Y685; Mut: VE-cadherin Y685F mutation; HG: high glucose; siNDPKB: NDPKB siRNA; TMG: Thiamet-G; OSMI-4: O-GlcNAc Transferase Inhibitor 4. *P < 0.05, **P < 0.01, ***P < 0.001. Significance was determined by one-way ANOVA with Tukey's post hoc test for multiple comparisons. All overall P < 0.001.

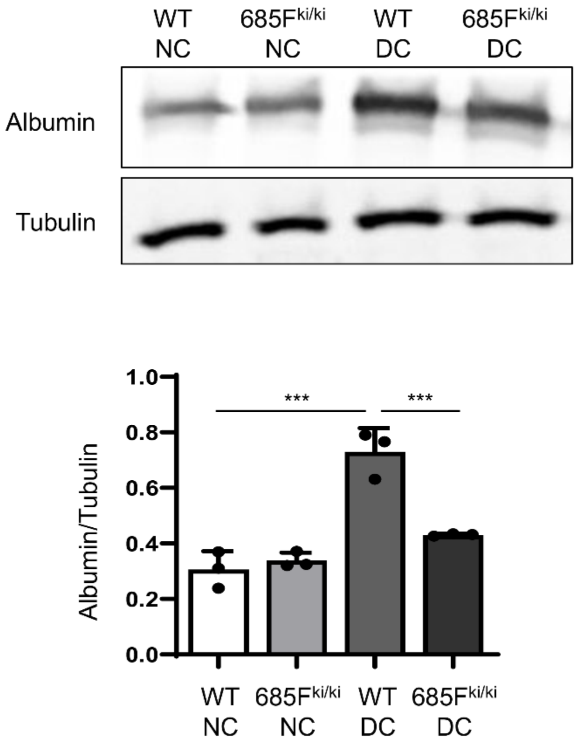

Supplementary Fig. 14: VE-cadherin Y685F$^{ki/ki}$ mitigates retinal vascular leakage in 6-month diabetic mice using albumin

Retinal vascular leakage was evaluated via immunoblotting and quantitation of immunocomplexes of albumin in retinal protein extracts from 6-month diabetic Y685F$^{ki/ki}$ retinas. n = 3. Overall $P < 0.001$. NC: nondiabetic; DC: diabetic; WT: wild-type; VEC Y685F$^{ki/ki}$: VE-cadherin Y685F knock-in. ***$P < 0.001$. Significance was determined by one-way ANOVA with Tukey's post hoc test for multiple comparisons.

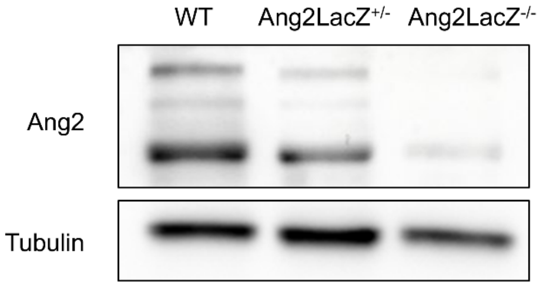

Supplementary Fig. 15: Test of an appropriate Ang2 antibody for its semi-quantitation in mouse retinas

Representative western blot of mouse retina using antibody PA5-27297 showed Ang2 in WT, AngLacZ$^{+/-}$ and Ang2LacZ$^{-/-}$ retinas. WT: wild-type Ang2LacZ mice; $^{+/-}$: heterozygous Ang2LacZ mice; $^{-/-}$: homozygous Ang2LacZ mice.

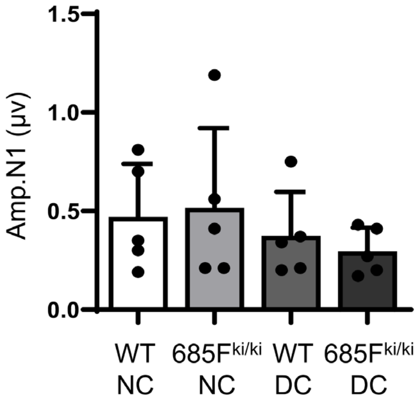

Supplementary Fig. 16: N1-wave amplitudes in ERG

Quantification of N1-wave in ERG of 3-month diabetic Y685F$^{ki/ki}$ retinas. n = 5. Overall P = 0.5972. NC: nondiabetic; DC: diabetic; WT: wild-type; VEC Y685F$^{ki/ki}$: VE-cadherin Y685F knock-in. Statistical analysis was performed by one-way ANOVA with Tukey's post hoc test for multiple comparisons.

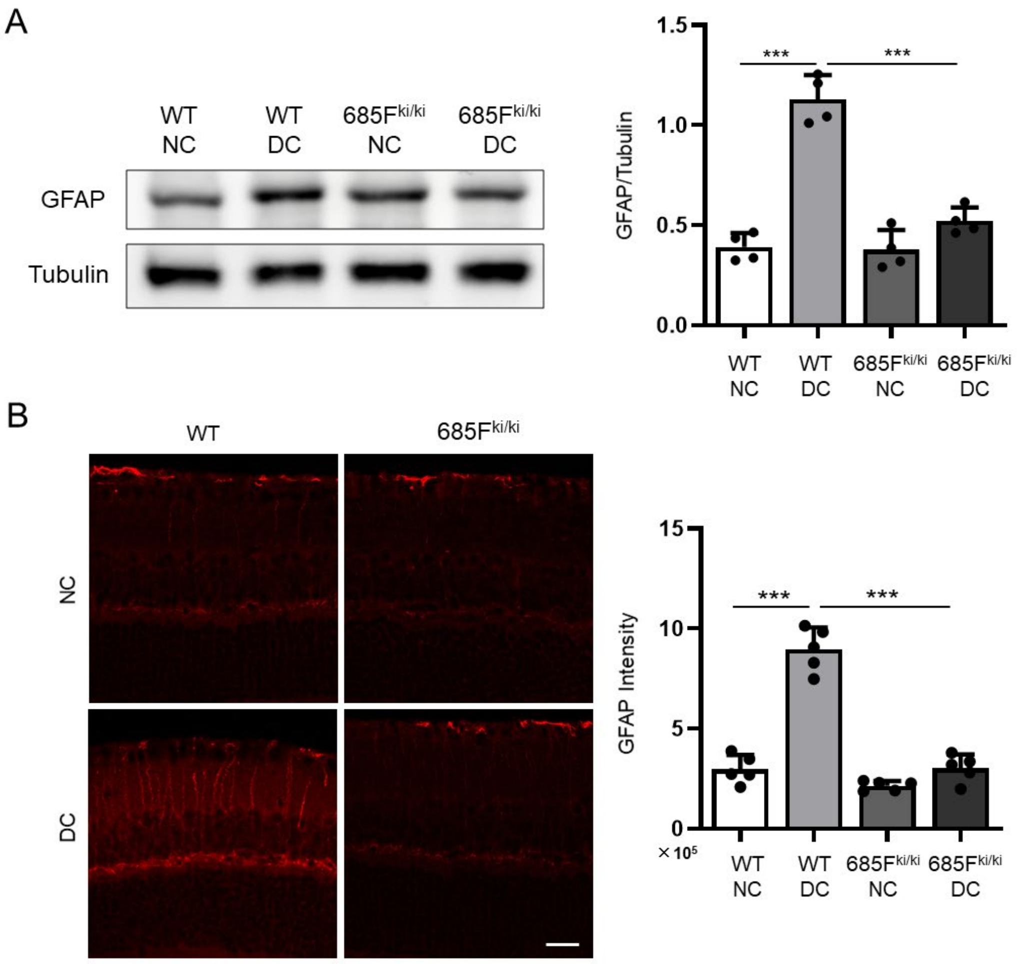

Supplementary Fig. 17: Diabetes-induced Müller cell activation is suppressed by Y685F mutation in the diabetic retina

GFAP immunoblotting and statistical analysis in retinal proteins (A). n=4. The GFAP expression and statistical analysis in retinal paraffin sections (B). n=5. NC: nondiabetic; DC: diabetic; WT: wild-type; $685F^{ki/ki}$: VE-cadherin Y685F knock-in. ***$P < 0.001$. Significance was determined by one-way ANOVA with Tukey's post hoc test for multiple comparisons, with all overall $P < 0.001$. Scale bar: 25 μm.

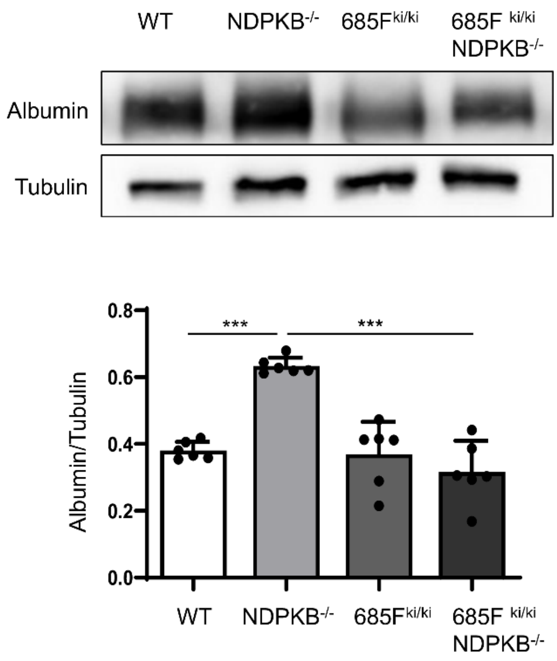

Supplementary Fig. 18: VE-cadherin Y685F mutation attenuates NDPKB deficiency-induced retinal hyperpermeability in 8-month-old mice

Immunoblotting and quantification of retinal permeability using albumin in 8-month-old VEC Y685F$^{ki/ki}$NDPKB$^{-/-}$ double transgenic mice. n = 6. Overall P < 0.001. WT: wild-type; 685F$^{ki/ki}$: VE-cadherin Y685F knock-in; NDPKB$^{-/-}$: NDPKB homozygous. 685F$^{ki/ki}$NDPKB$^{-/-}$: VEC Y685F$^{ki/ki}$NDPKB$^{-/-}$ double transgenic mice. ***P < 0.001. Significance was determined by one-way ANOVA with Tukey's post hoc test for multiple comparisons.

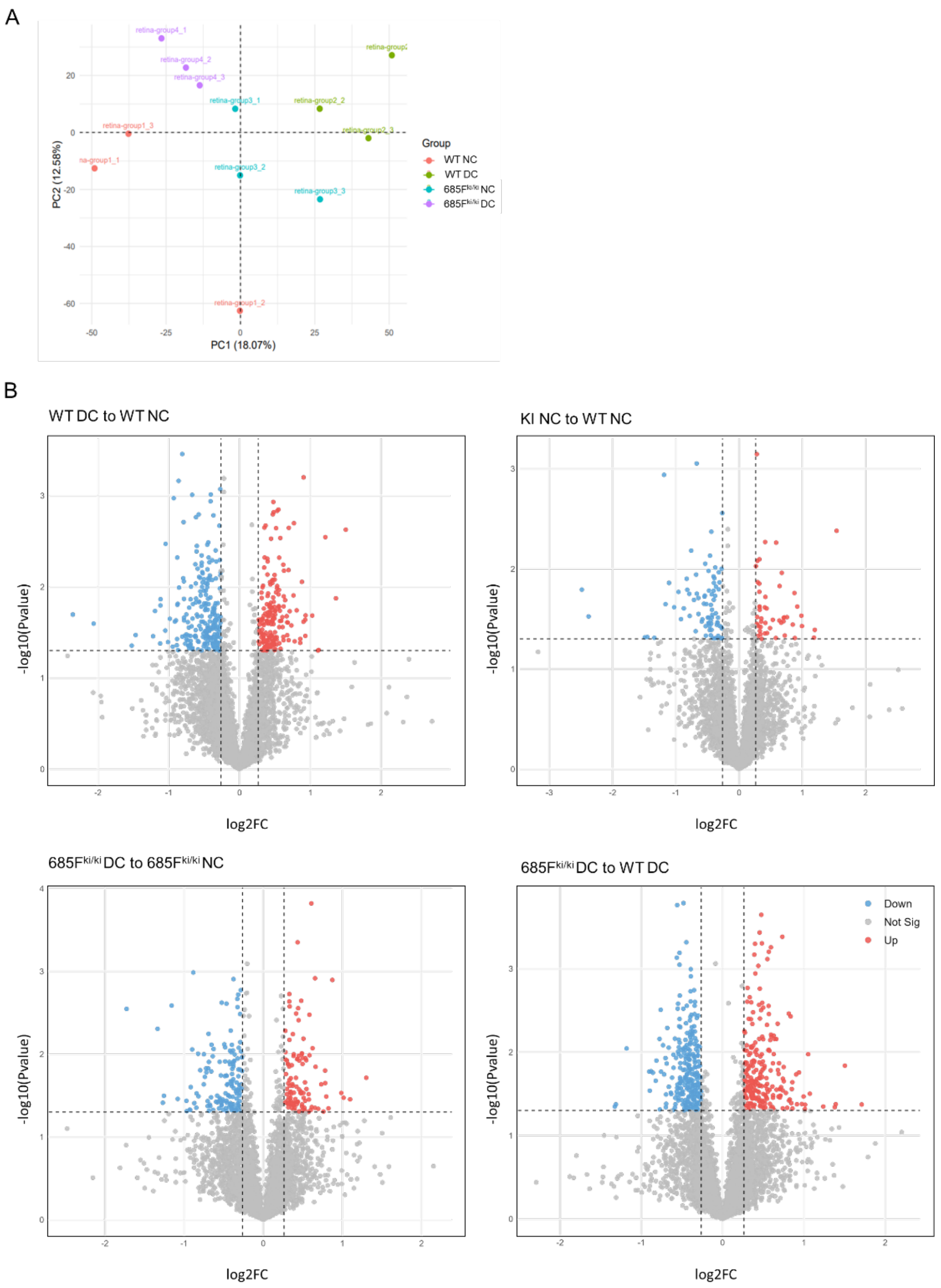

Supplementary Fig. 19: Proteomics analysis with PCA and volcano plots

(A) PCA of the O-GlcNAc-enriched retinal proteome from NC WT, DC WT, NC VEC Y685F$^{ki/ki}$ and DC VEC Y685F$^{ki/ki}$ retinas. (B) Volcano plots for four group comparisons with points colored by increased (red), decreased (blue), or non-significant (gray) O-GlcNAcylation ($P < 0.05$; fold change ≥ 1.2). NC: nondiabetic; DC: diabetic; WT: wild-type; 685F$^{ki/ki}$: VE-cadherin Y685F knock-in.

Supplementary Table 1: Antibodies, experimental models, and reagents used in the study

Reagent	Source	Identifier
Immunoblotting: primary antibody		
goat-anti-Ang-2	Santa Cruz	sc-7017
goat-anti-VE-cadherin	Santa Cruz	sc-6458
goat-anti-VE-cadherin	R&D Systems	AF1002
mouse-anti-GFP	Santa Cruz	sc-9996
mouse-anti-NDPKB	Kamiya Biomedical	MC-412
mouse-anti-O-GlcNAc	Abcam	ab2739
mouse-anti-tubulin	Sigma-Aldrich	T6557
mouse-anti-VE-cadherin	Santa Cruz	sc-9989
rabbit-anti-albumin	MP Bio	55442
rabbit-anti-Ang-2	Thermo Fisher Scientific	PA5-27297
rabbit-anti-Gβ	Santa Cruz	sc-378
rabbit-anti-MGEA5	Proteintech	14711-1-AP
rabbit-anti-OGT	Sigma-Aldrich	O6264
rabbit-anti-phospho-VEC (Tyr658)	Invitrogen	44-1144G
rabbit-anti-phospho-VEC (Tyr685)	Abcam	ab119785
rabbit-anti-phospho-VEC (Tyr731)	Invitrogen	44-1145G
Immunoblotting: secondary antibody		
goat-anti-rabbit IgG (H+L)-HRP	Thermo Fisher Scientific	31460
rabbit-anti-goat IgG-HRP	Sigma-Aldrich	AP106P
rabbit-anti-mouse IgG (H+L)-HRP	Thermo Fisher Scientific	61-6520
Immunoprecipitation		
rabbit-anti-VE-cadherin	Cell Signaling	D87F2
Immunofluorescence		
DAPI	Sigma-Aldrich	D9542
goat-anti-mouse Alexa Fluor 488	Invitrogen	A28175
goat-anti-mouse Alexa Fluor 555	Invitrogen	A32727
goat-anti-rabbit Cy3	Jackson Laboratories	111-165-144
goat-anti-VE-cadherin	R&D System	AF1002
Lectin-TRITC	Sigma-Aldrich	L5264
mouse-anti-VE-cadherin	Enzo Life Sciences	ALX-803-305
rabbit-anti-GFAP	Dako	Z0334
rabbit-anti-goat conjugated with Alexa Fluor 488	Thermo Fisher Scientific	A-11078
Chemical reagents		
Antibiotics	Sigma-Aldrich	P4333
CellTracker™ Red CMTPX Dye	Thermo Fisher	C34552
D-glucose	Sigma-Aldrich	G7021
Dextran-FITC	Sigma-Aldrich	FD70S
ECBM	Promocell	C-22210
ECL femto	Thermo Scientific	34095
ECL Lumi-light	Roche	SL100309
FCS	Promocell	C-37350
Ham's F-12 Nutrient Mix	Thermo Fisher	21765029
Lantus® 100 Einheiten	Sanofi	PZN / 05387771
L-glucose	Sigma-Aldrich	921-60-8
Lipofectamine RNAiMAX	Invitrogen	2463615
MCDB 131 medium	Thermo Scientific	10372019
OSMI-4	Targetmol	T12328

OptiPRO medium	Thermo Fisher	2026928
Phosphatase inhibitor	Roche	4906845001
Protease inhibitor	Roche	11697498001
Protein A/G PLUS-Agarose	Santa Cruz	sc-2003
Puromycin	Sigma-Aldrich	P7255
Recombinant Human VEGF	R&D System	293-VE
Roti-block	Carl Roth	A151.2
STZ	Merck	S0130
sWGA	Vector Laboratories	AL-1023S-2
Subcellular Protein Fractionation Kit	Thermo Fisher	78840
Thiamet G	Sigma-Aldrich	SML0244
Experimental models: Cell lines		
Human: brain vascular pericytes	ScienCell Research Laboratories	
Human: HUVECs	Medical Faculty Mannheim, University of Heidelberg, Germany	
Mouse: MBMECs	Medical Faculty Mannheim, University of Heidelberg, Germany	
Experimental models: Animals		
$Ins2^{Akita}$ mice	Jackson Laboratory	
$NDPKB^{-/-}$ mice	Helen L. and Martin S. Kimmel Center for Biology and Medicine at the Skirball Institute for Biomolecular Medicine in New York	
VEC $Y685F^{ki/ki}$ mice	From Prof. Dietmar Vestweber, Max-Planck-Institute for Molecular Biomedicine, Münster, Germany	
VEC $Y685F^{ki/ki}$ $NDPKB^{-/-}$ mice	Medical Faculty Mannheim, University of Heidelberg	
siRNA		
NDPK B siRNA (5’-AGGUAGUGUAAUCGCCUUG-3’)	Eurofins	
Scrambled siRNA (5’-AAC UGG UUG ACU ACA AGU CUU-3’)	Eurofins	
Software and device		
AnalysisPro software	Olympus Opticals	
GraphPad Prism 10	GraphPad Software	
ImageJ	National Institutes Of Health (NIH) software	
Leica DMRE	Leica	
Leica SP8	Leica	
Olympus fluorescence microscope	Olympus	
RETImap	Roland Consult	
ViLBER FUSION FX	ViLBER	
100-diopter contact lens	Roland Consult	

Supplementary Table 2: Differentially O-GlcNAcylated proteins identified by comparative proteomic analysis (see data sheet in online proteomics supplemental table)

This supplemental Excel table contains the results of comparative proteomic analysis of retinal O-GlcNAcylated proteins between DC WT to NC WT (A), DC VEC Y685F$^{ki/ki}$ to DC WT (B), NC VEC Y685F$^{ki/ki}$ to NC WT (C) and DC VEC Y685F$^{ki/ki}$ to NC VEC Y685F$^{ki/ki}$ (D). Fold changes were calculated as the ratio of group means (log2 scale), and proteins were considered differentially enriched at nominal $p < 0.05$ and absolute fold change $|\log2FC| \geq 0.263$. Proteins that were increased in DC WT compared with NC WT but decreased in DC VEC Y685F$^{ki/ki}$ compared with DC WT, together with their associated enriched pathways, are listed in (E). Proteins showing the opposite pattern are listed in (F). NC: nondiabetic; DC: diabetic; WT: wild-type; VEC Y685F$^{ki/ki}$: VE-cadherin Y685F knock-in.

1. Gross S, Devraj K, Feng Y, Macas J, Liebner S, and Wieland T. Nucleoside diphosphate kinase B regulates angiogenic responses in the endothelium via caveolae formation and c-Src-mediated caveolin-1 phosphorylation. *J Cereb Blood Flow Metab.* 2017;37(7):2471-84.
2. Hu J, Dziumbla S, Lin J, Bibli SI, Zukunft S, de Mos J, et al. Inhibition of soluble epoxide hydrolase prevents diabetic retinopathy. *Nature.* 2017;552(7684):248-52.
