# Supplementary material for: Disrupting VE-cadherin Y685 phosphorylation inhibits development of experimental diabetic and prediabetic retinopathy
Source: J Clin Invest. 2026 May 15;136(10):e195048. doi: 10.1172/JCI195048 (PMC13178665; doi:10.1172/JCI195048)

Unprocessed Western blots of Figure 1A

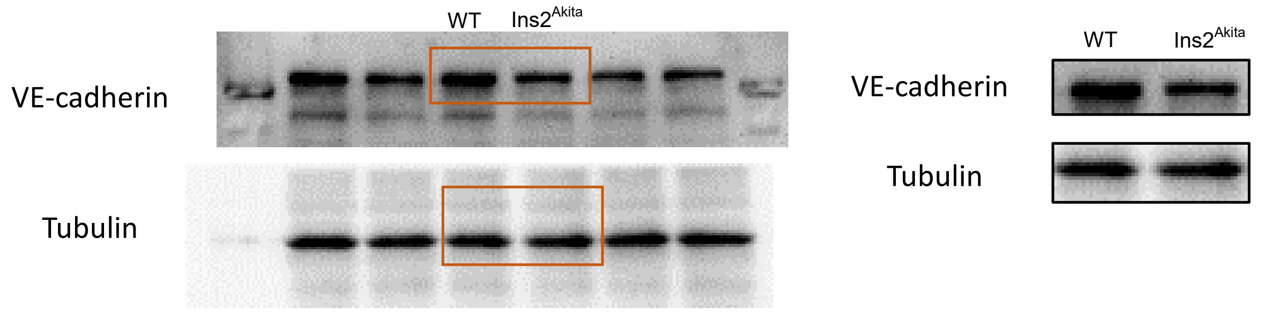

Unprocessed Western blots of Figure 1B

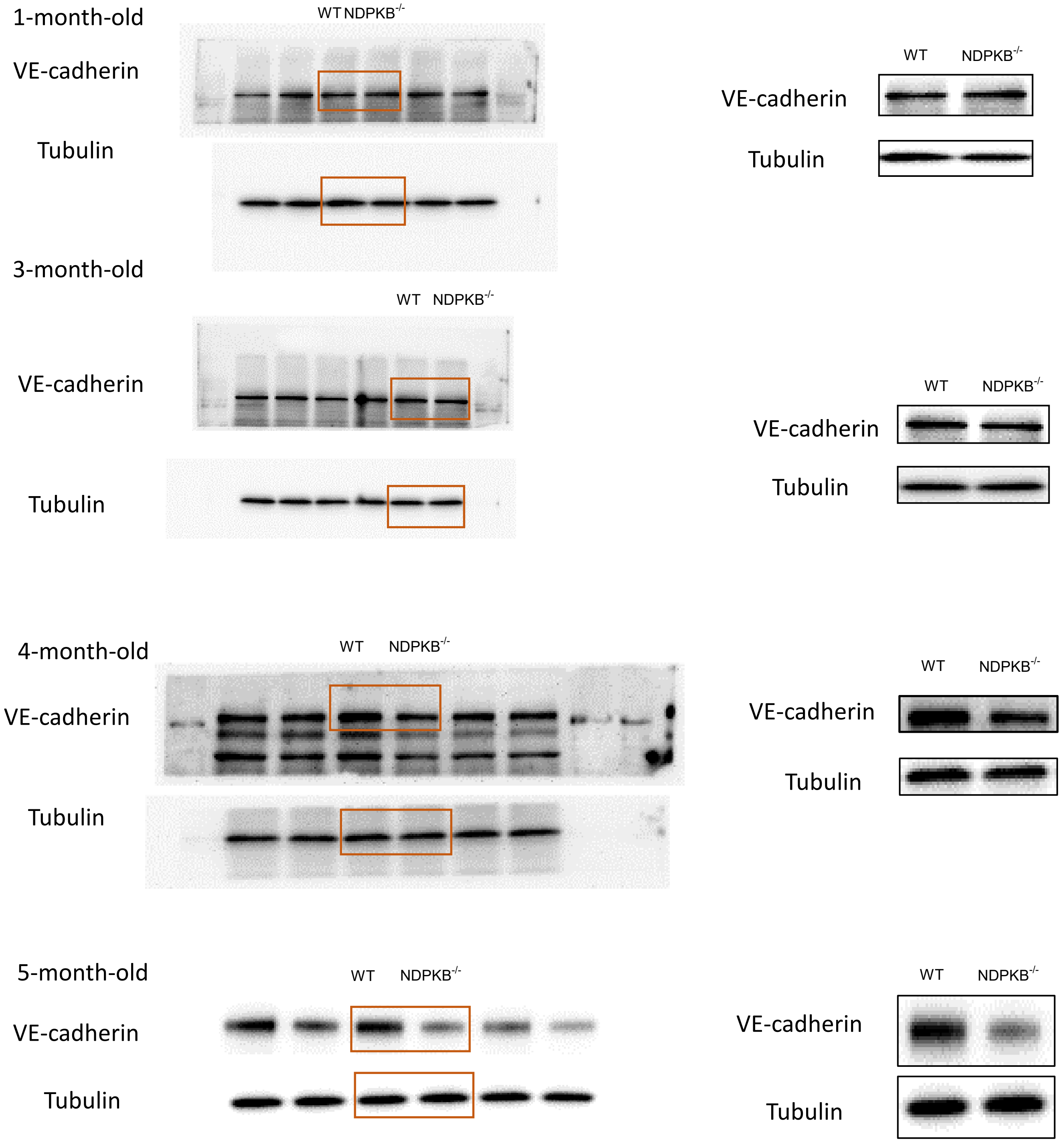

Unprocessed Western blots of Figure 1E

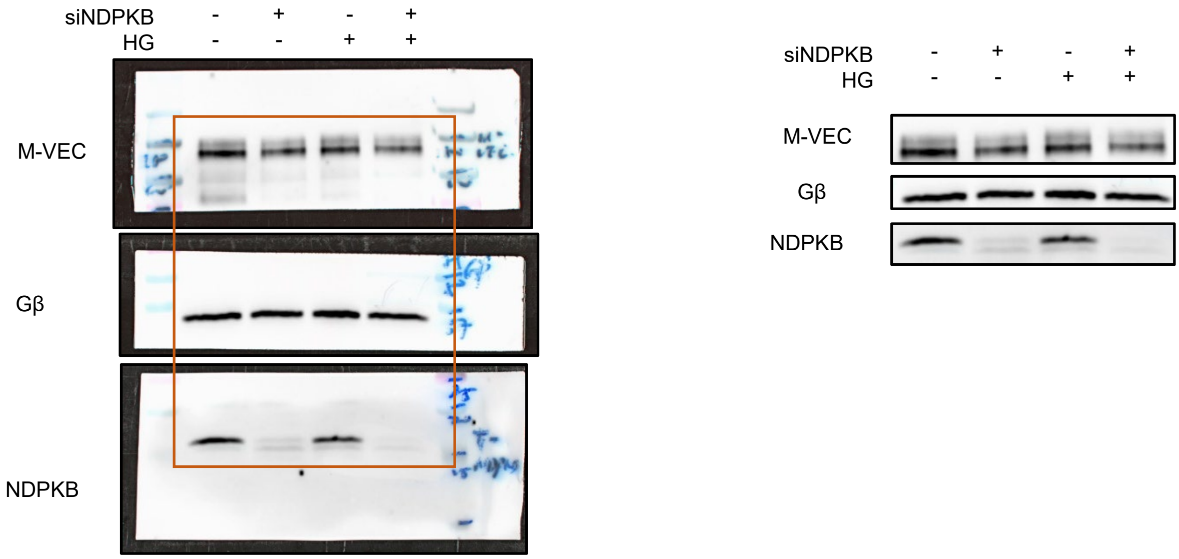

Unprocessed Western blots of Figure 1G

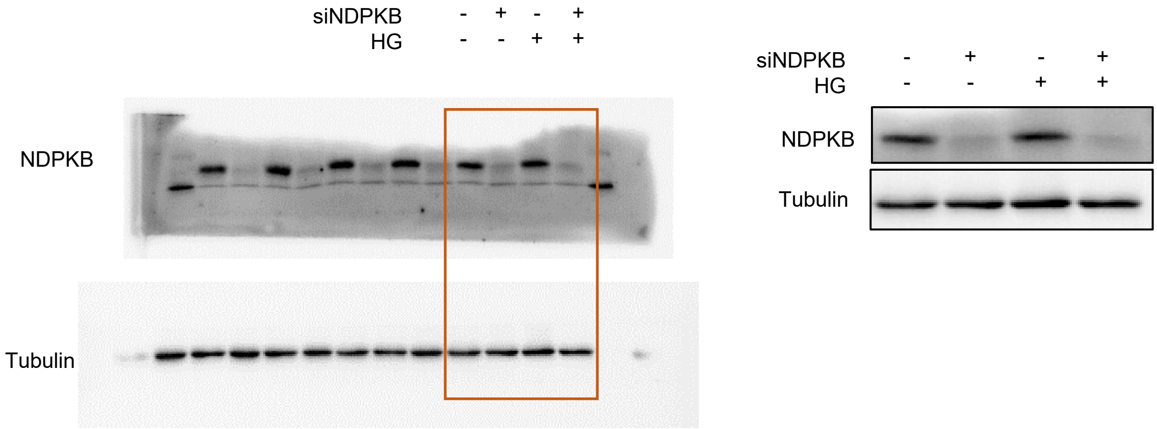

Unprocessed Western blots of Figure 3A

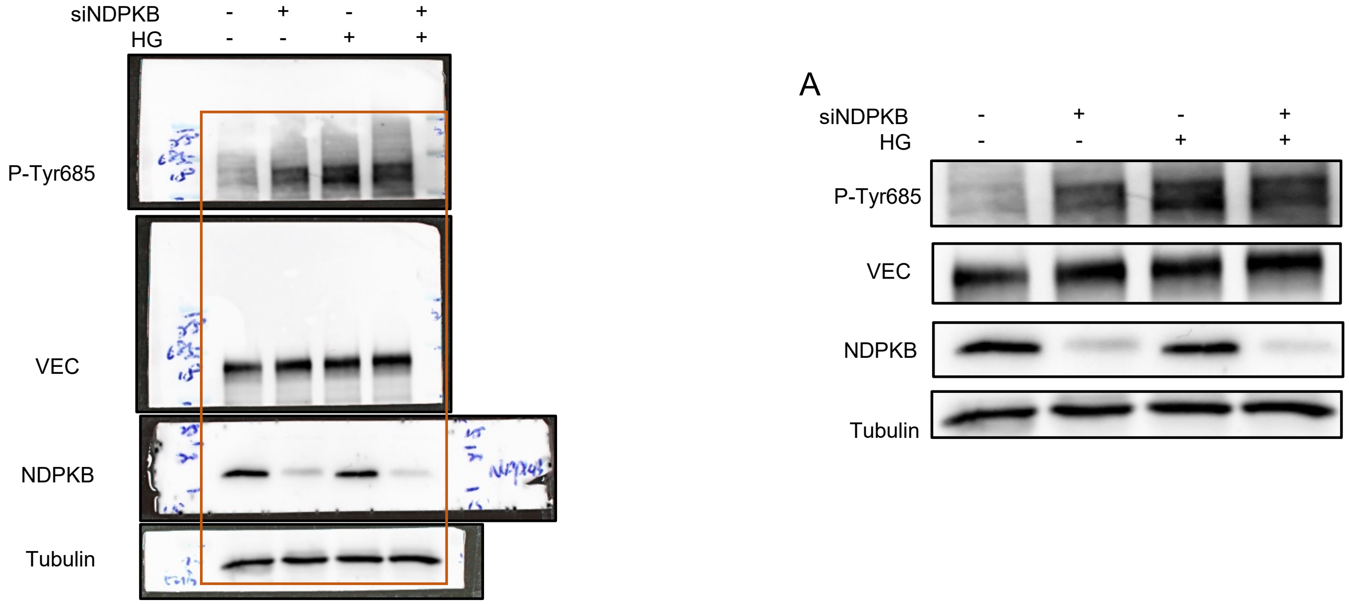

Unprocessed Western blots of Figure 3B

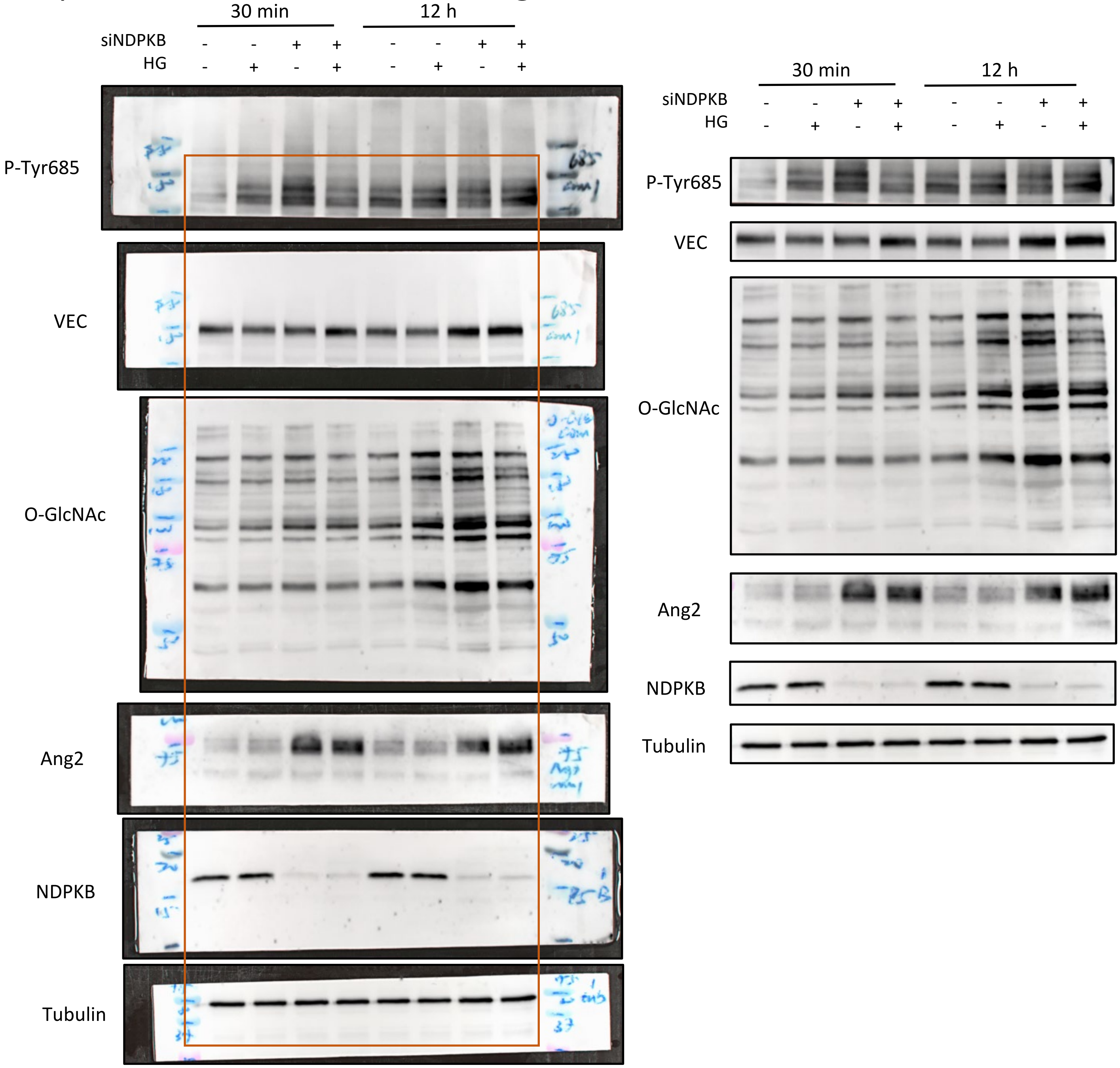

Unprocessed Western blots of Figure 4G

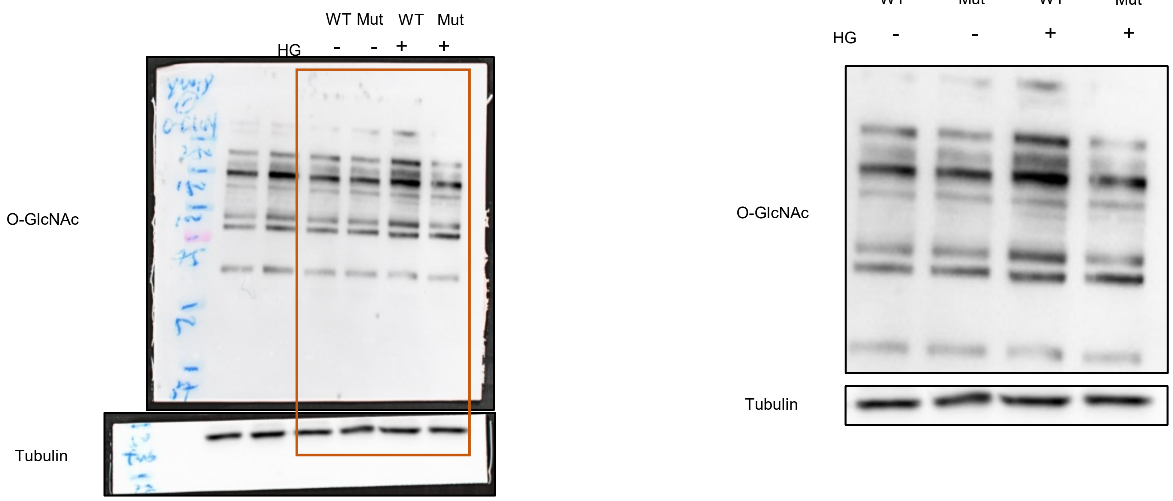

Unprocessed Western blots of Figure 4H

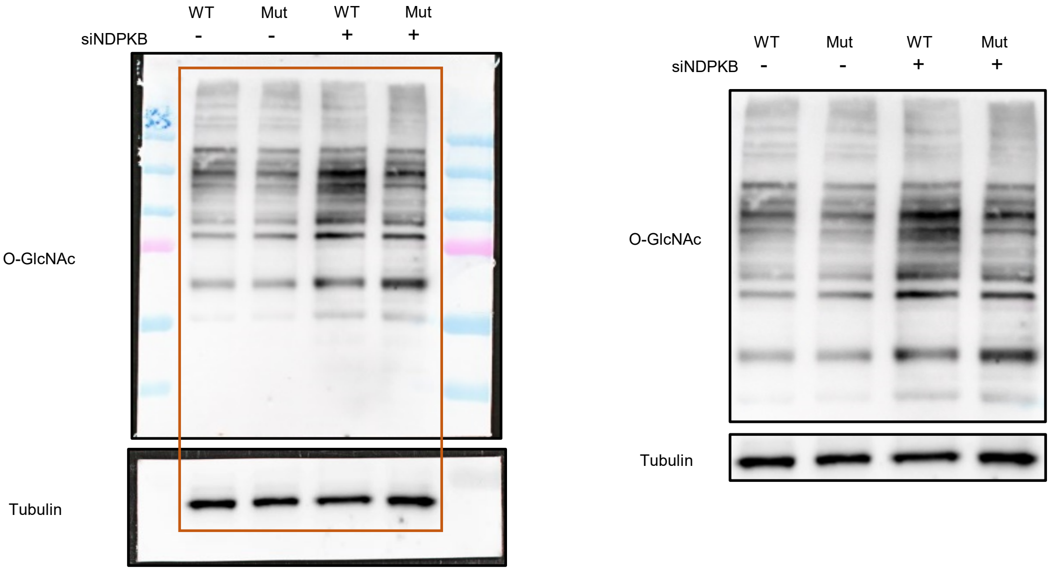

Unprocessed Western blots of Figure 4I

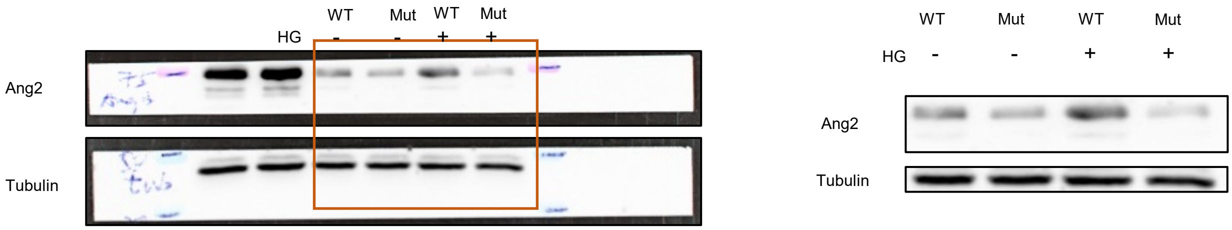

Unprocessed Western blots of Figure 4J

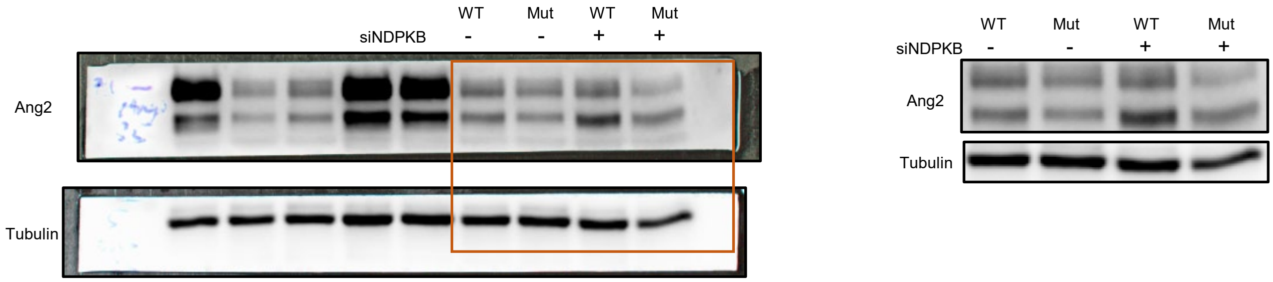

Unprocessed Western blots of Figure 5A

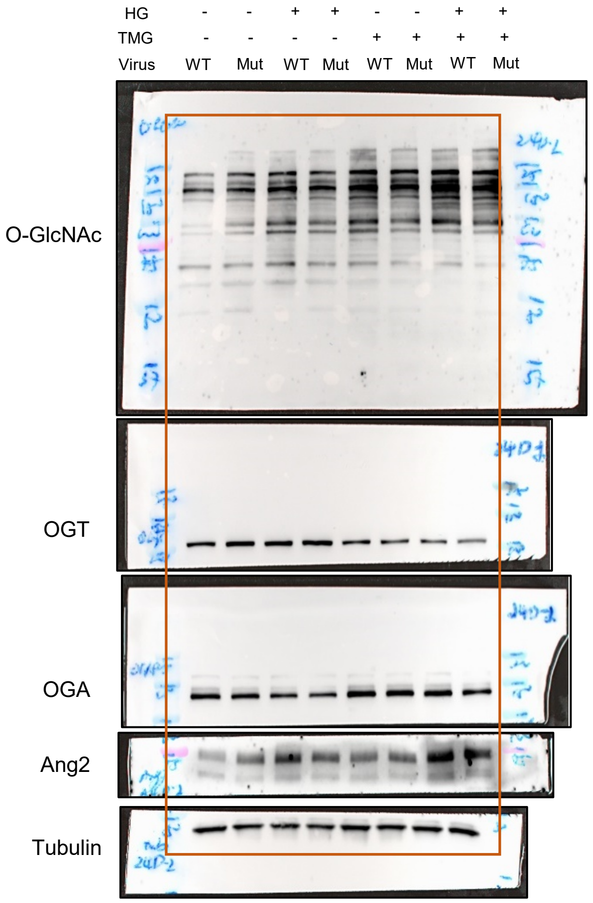

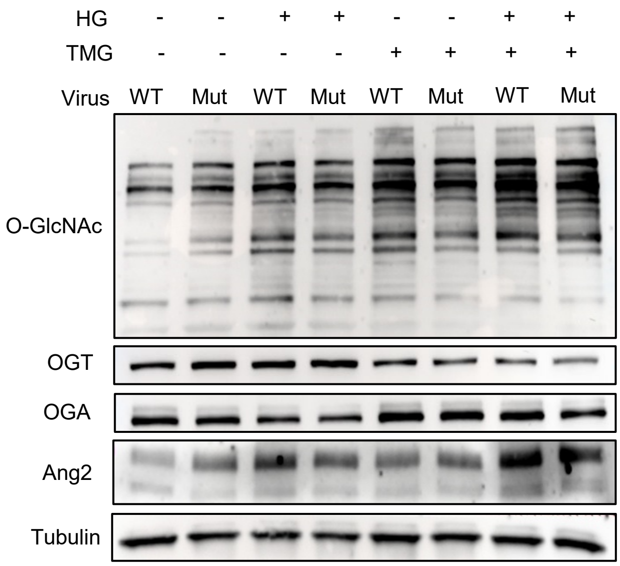

Unprocessed Western blots of Figure 5C

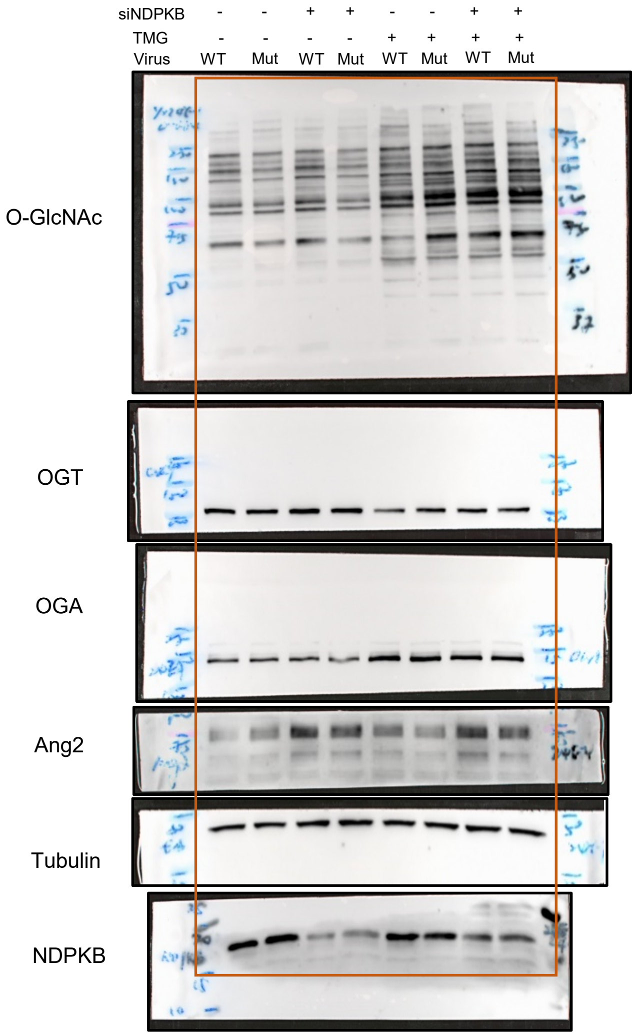

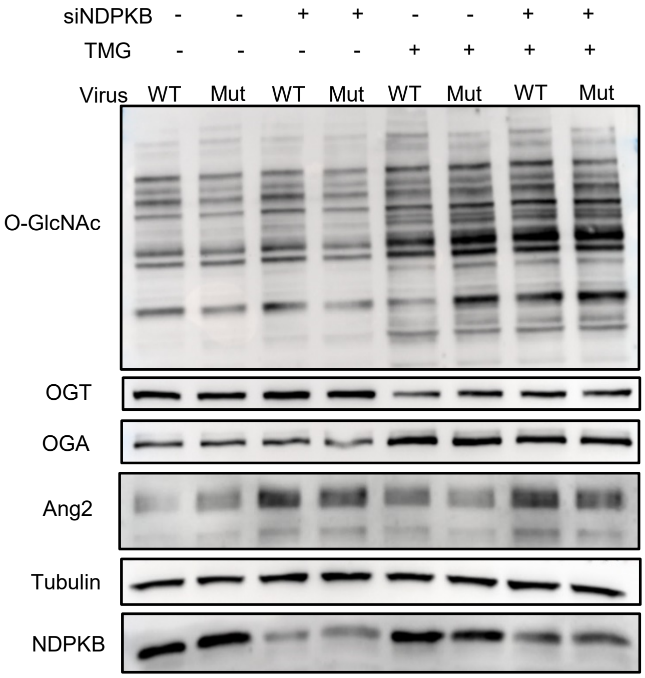

Unprocessed Western blots of Figure 5E

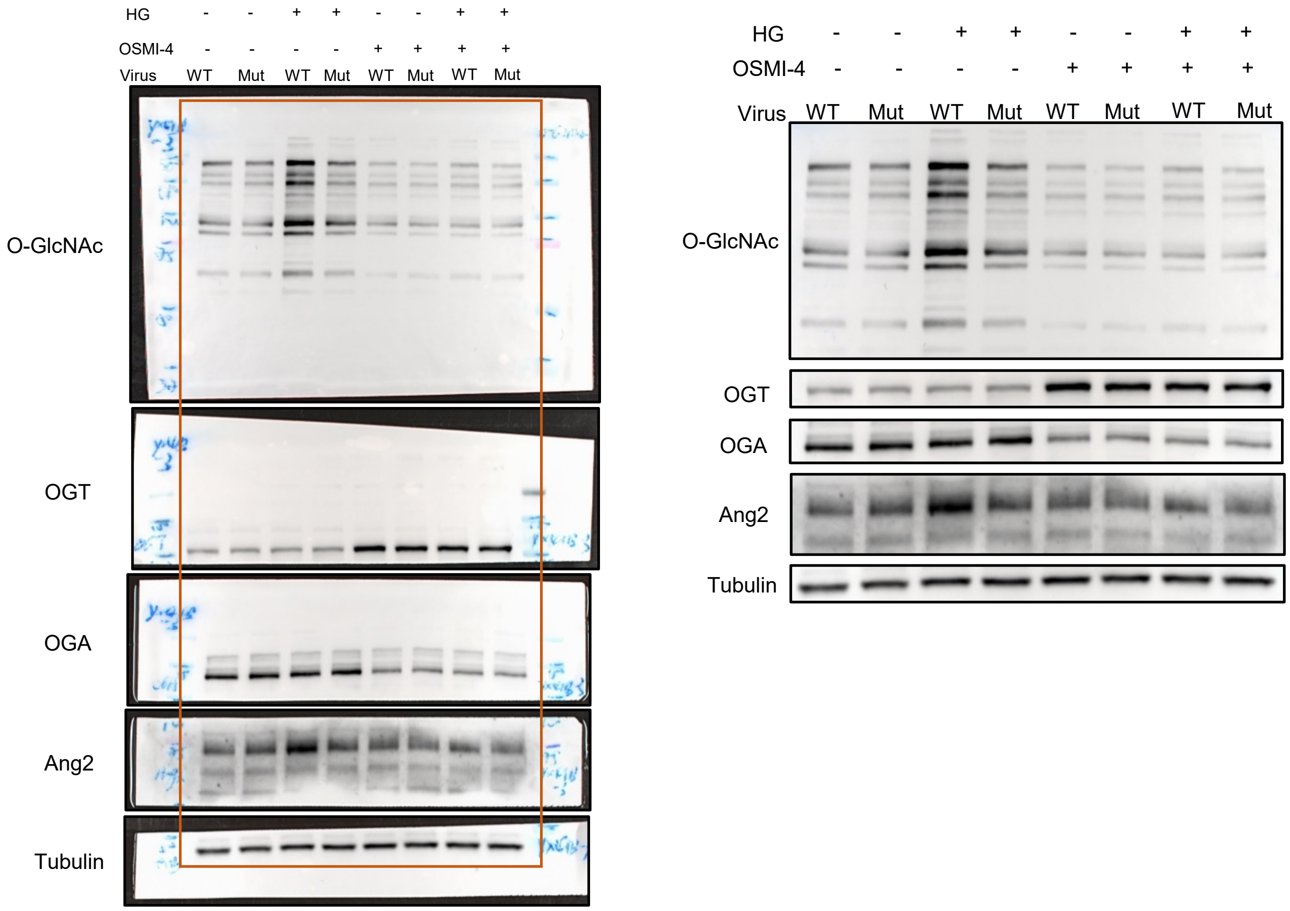

Unprocessed Western blots of Figure 5G

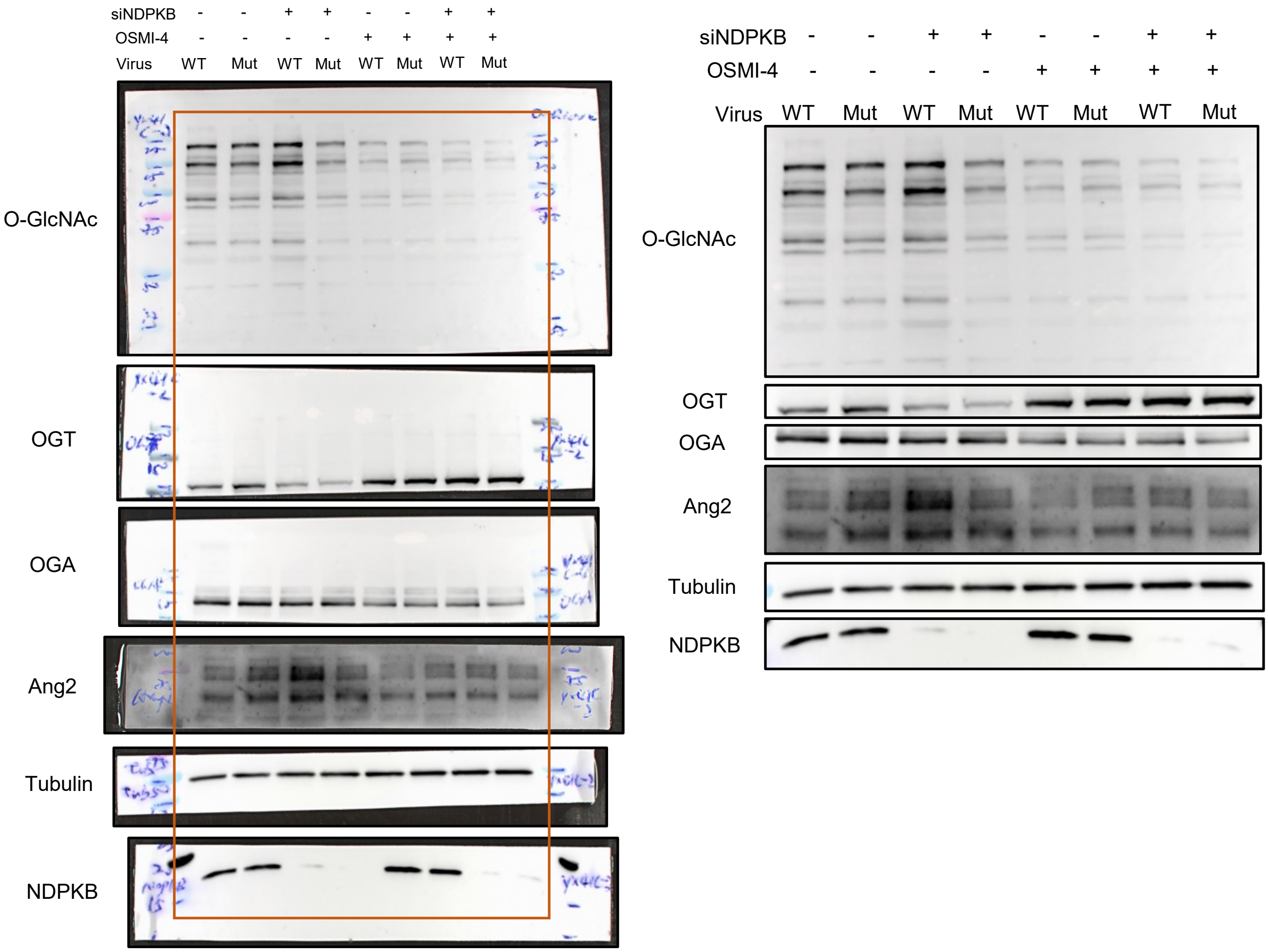

Unprocessed Western blots of Figure 6C

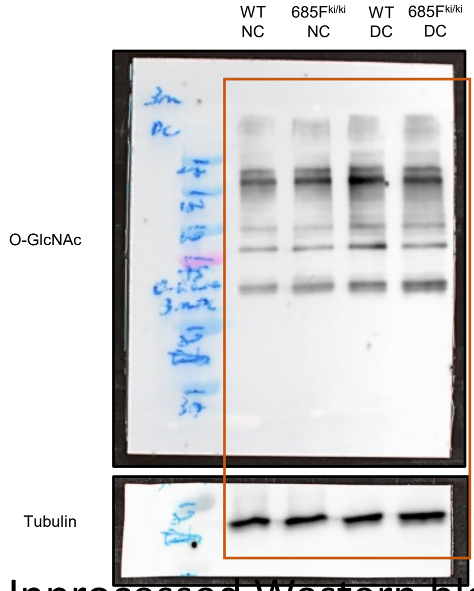

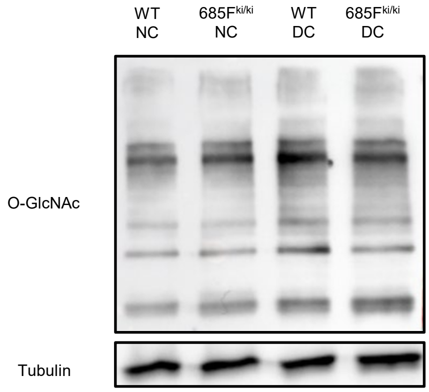

Unprocessed Western blots of Figure 6D

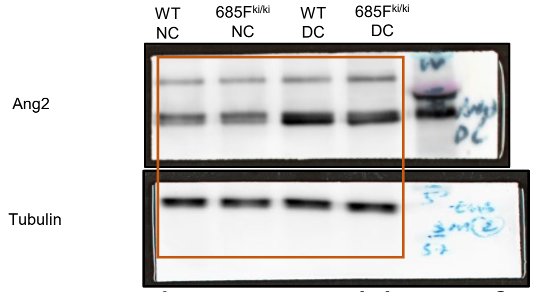

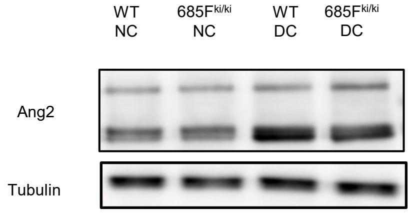

Unprocessed Western blots of Figure 6E

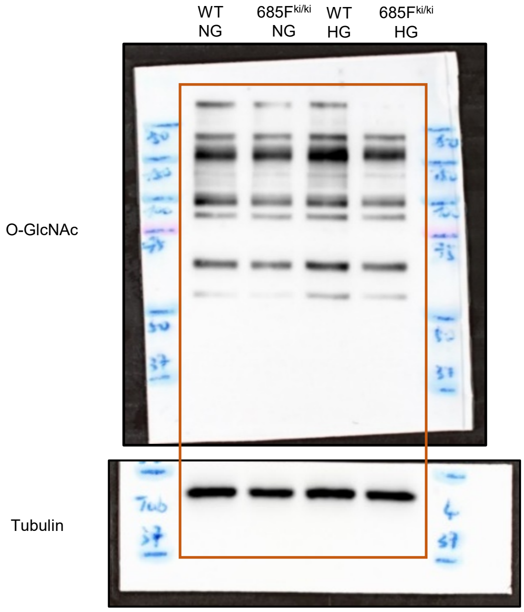

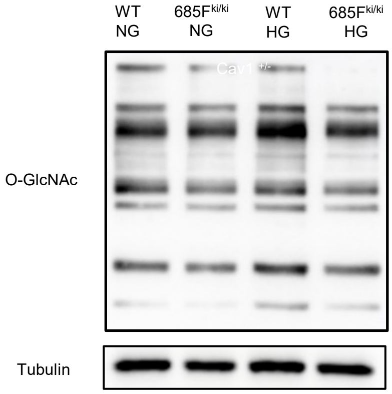

Unprocessed Western blots of Figure 6F

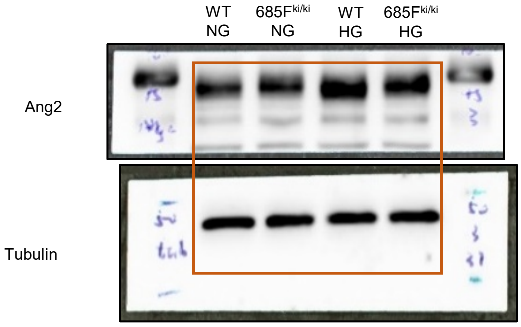

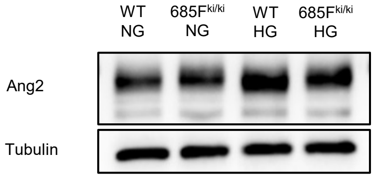

Unprocessed Western blots of Figure 7B

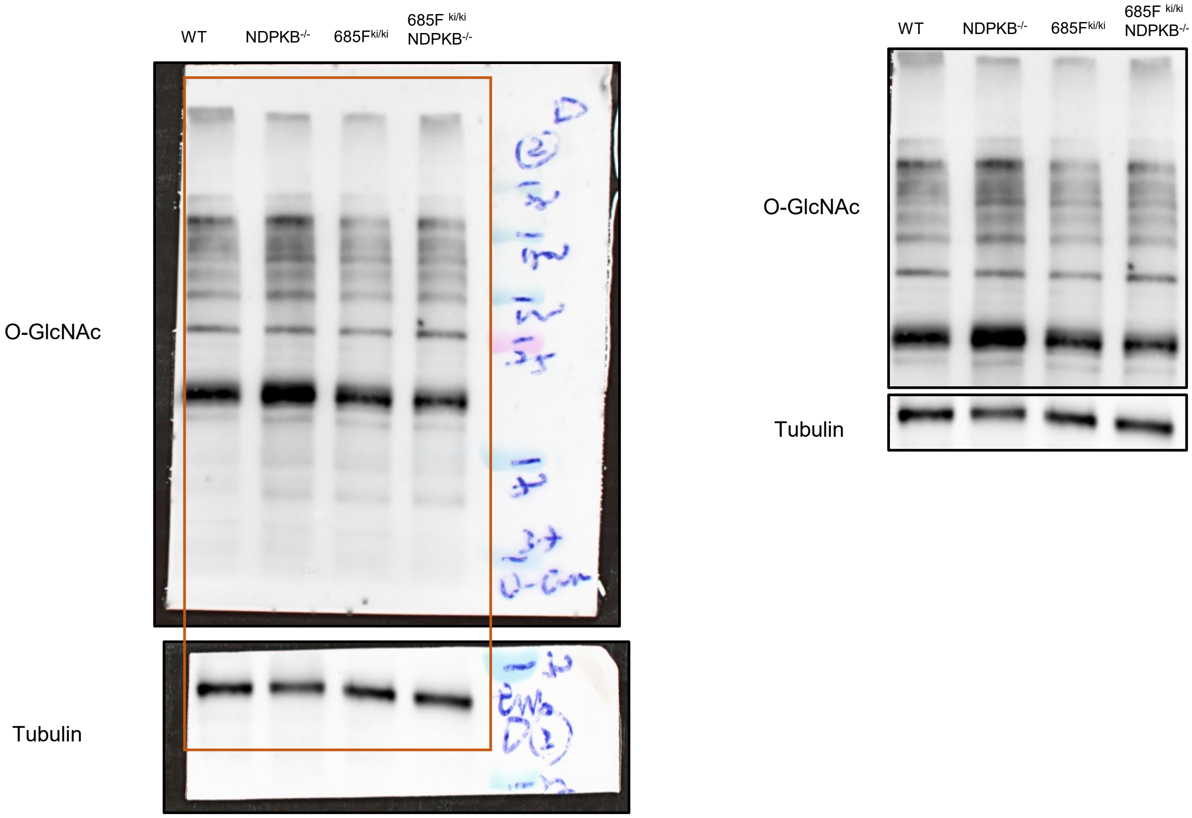

Unprocessed Western blots of Figure 7C

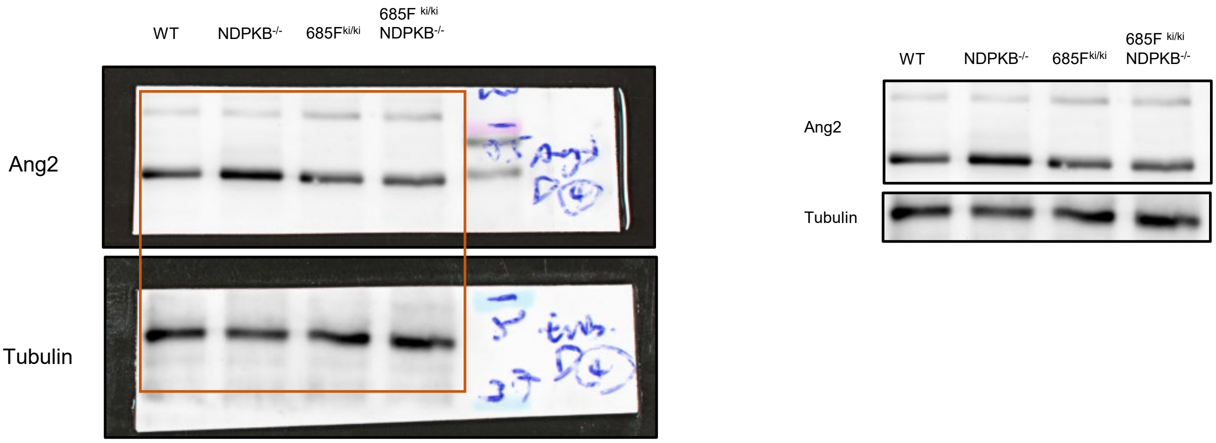

Unprocessed Western blots of Supplementary Figure 2

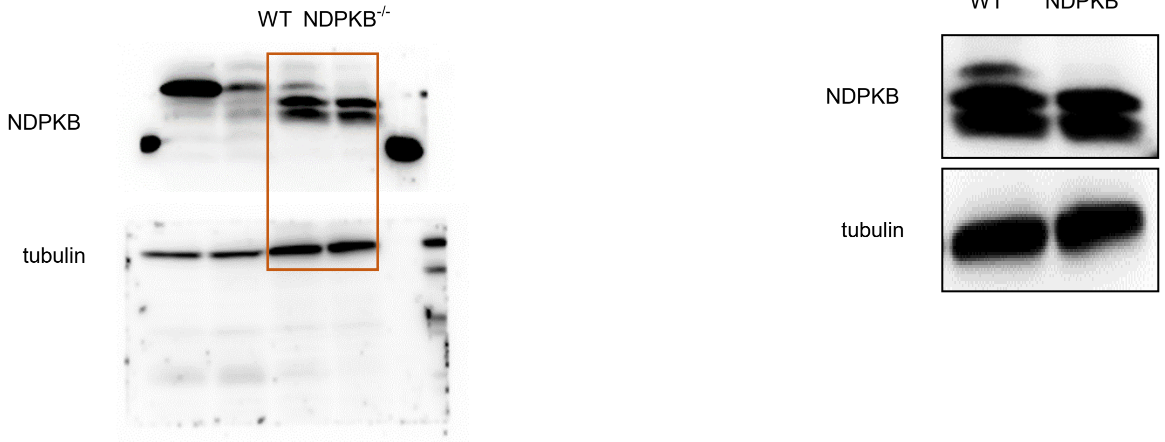

Unprocessed Western blots of Supplementary Figure 3

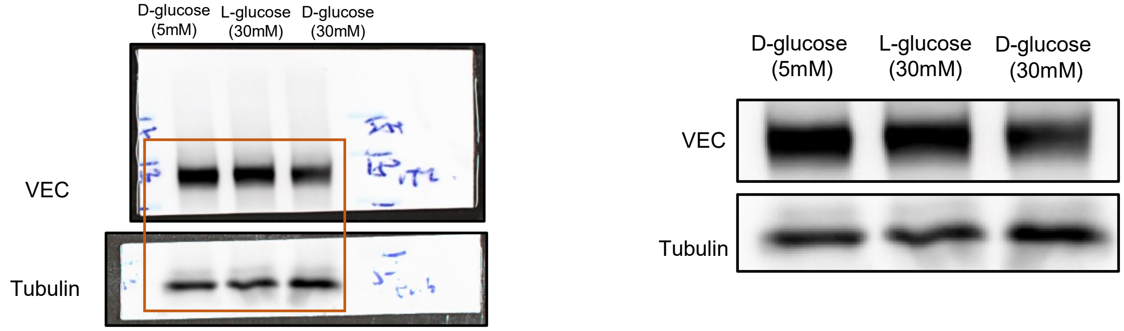

Unprocessed Western blots of Supplementary Figure 4

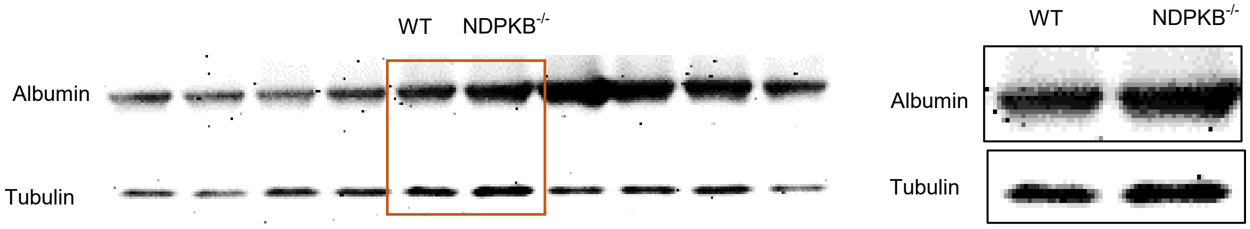

Unprocessed Western blots of Supplementary Figure 6

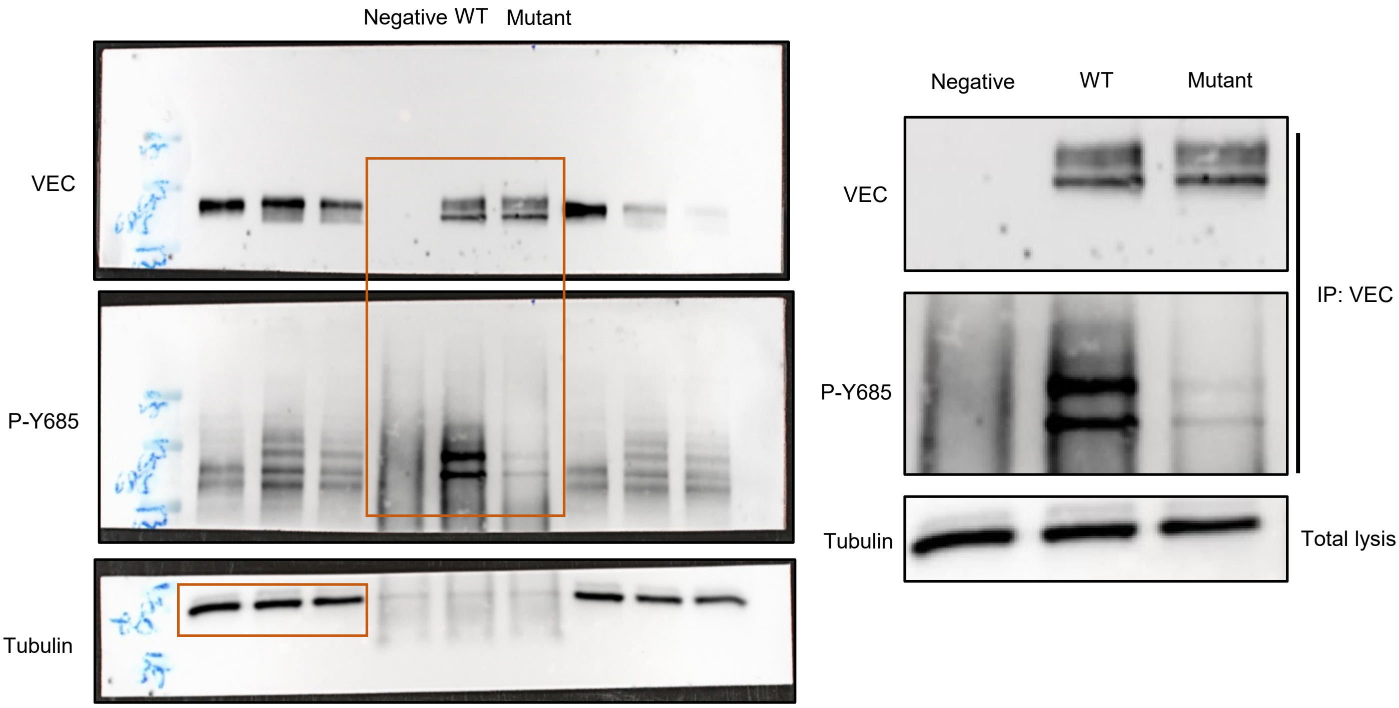

Unprocessed Western blots of Supplementary Figure 7

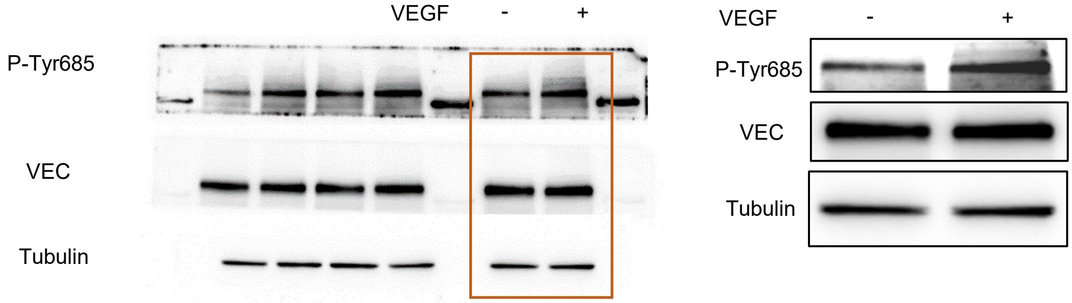

Unprocessed Western blots of Supplementary Figure 8

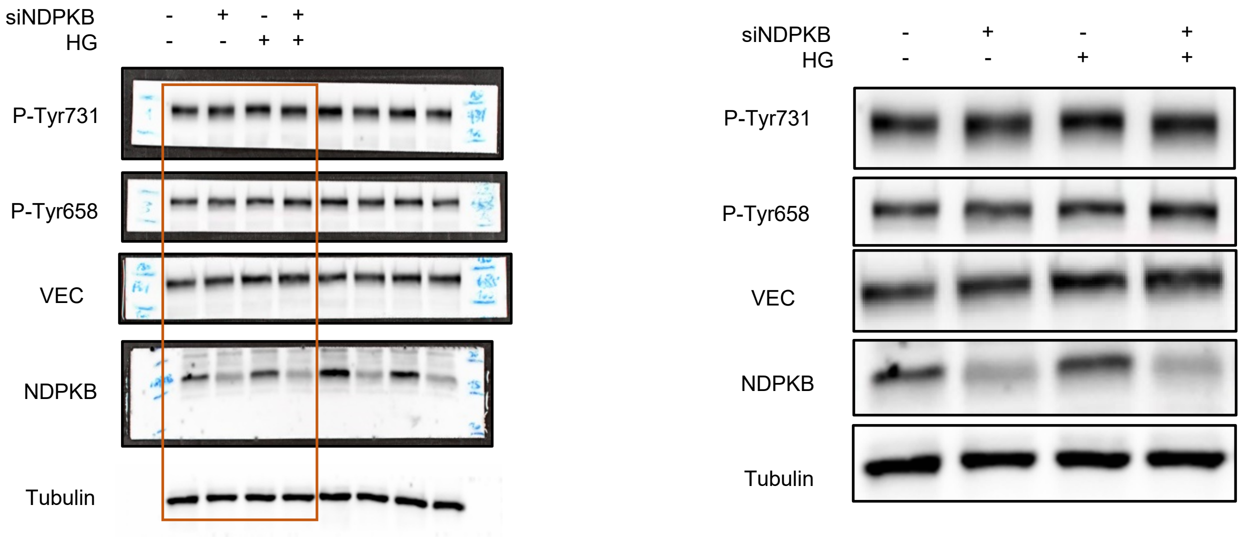

Unprocessed Western blots of Supplementary Figure 9

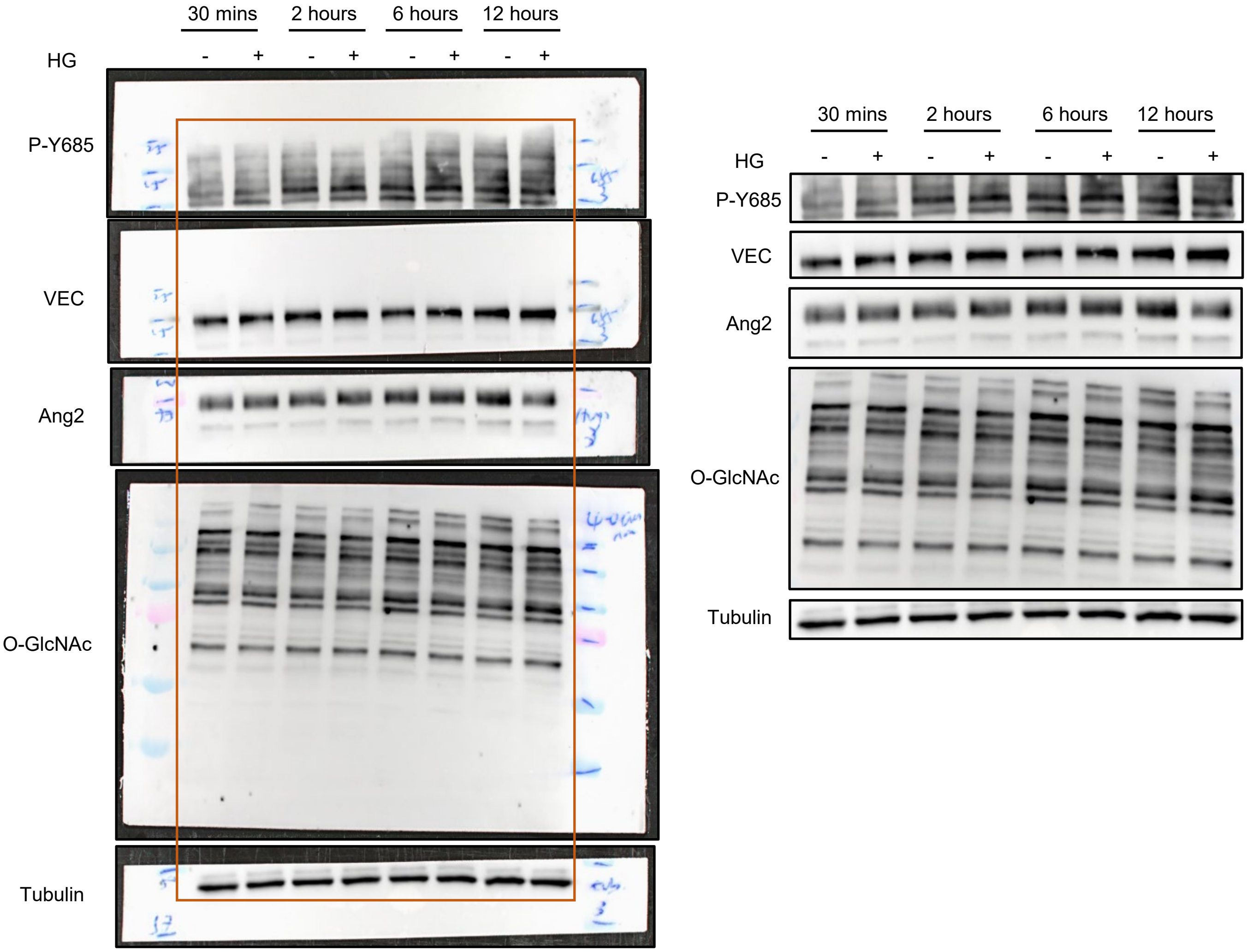

Unprocessed Western blots of Supplementary Figure 10A

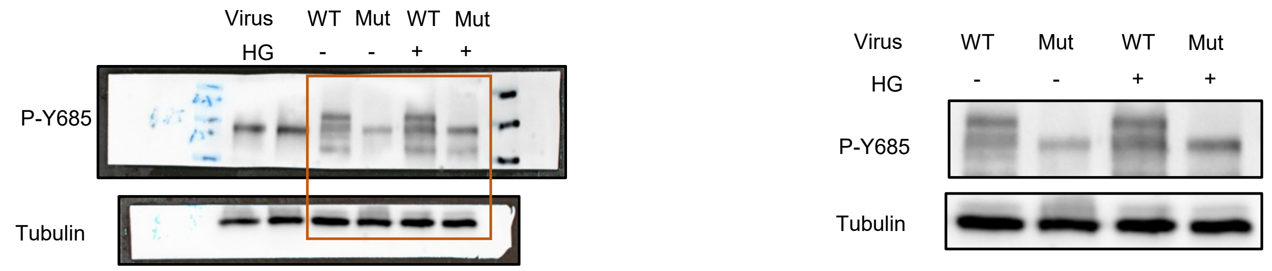

Unprocessed Western blots of Supplementary Figure 10B

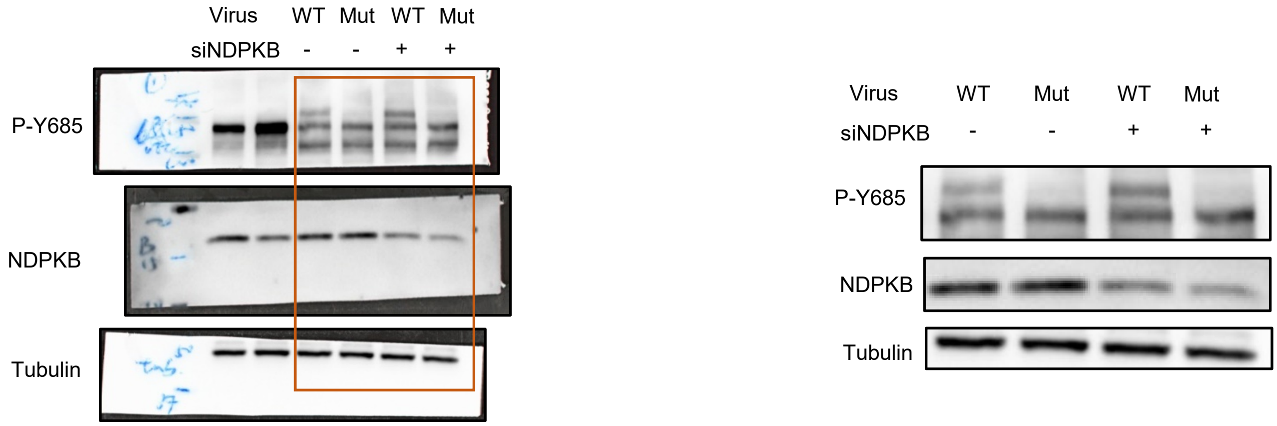

Unprocessed Western blots of Figure 14

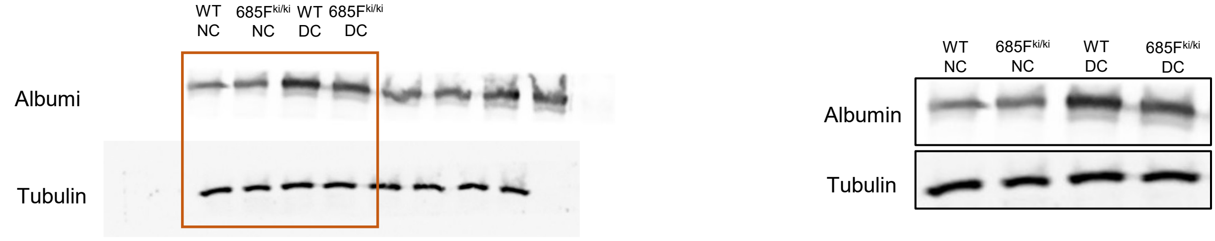

Unprocessed Western blots of Figure 15

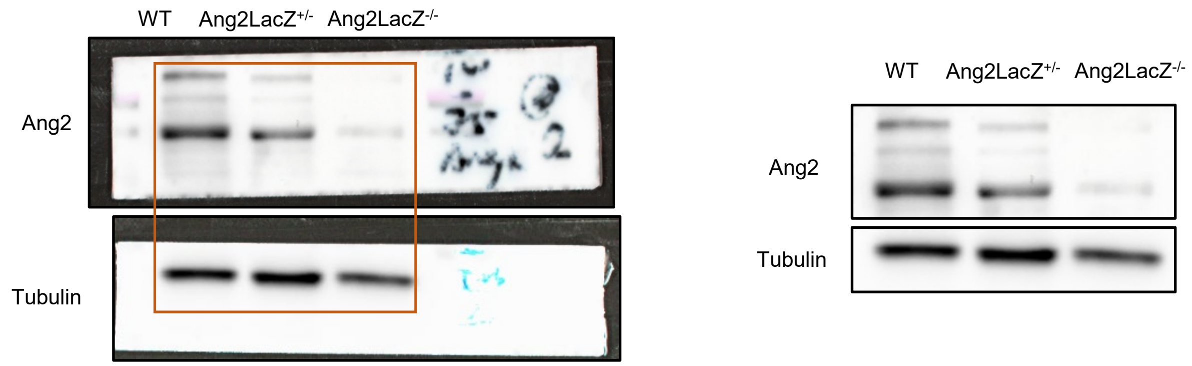

Unprocessed Western blots of Figure 17

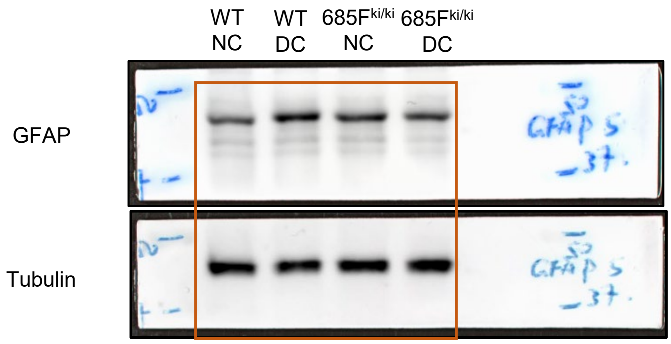

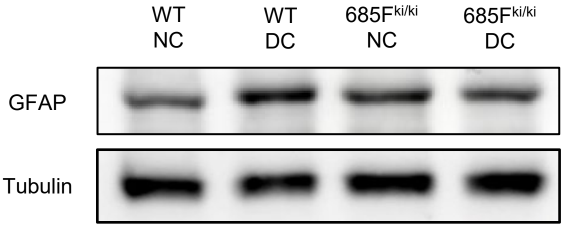

Unprocessed Western blots of Figure 18

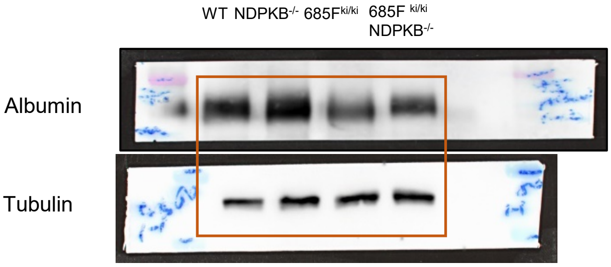

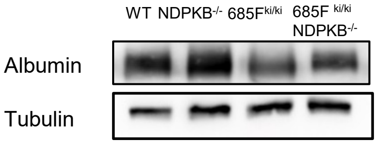

Supplement: Unedited blot and gel images [file jci-136-195048-s022.pdf]
